# Comparison of the secretory murine DNase1 family members expressed in *Pichia pastoris*

**DOI:** 10.1371/journal.pone.0253476

**Published:** 2021-07-30

**Authors:** Lukas Verhülsdonk, Hans Georg Mannherz, Markus Napirei

**Affiliations:** 1 Department of Anatomy and Molecular Embryology, Medical Faculty, Ruhr-University Bochum, Bochum, Germany; 2 Molecular and Experimental Cardiology, St. Josef-Hospital, Clinics of the Ruhr University Bochum, Bochum, Germany; Weizmann Institute of Science, ISRAEL

## Abstract

Soluble nucleases of the deoxyribonuclease 1 (DNase1) family facilitate DNA and chromatin disposal (chromatinolysis) during certain forms of cell differentiation and death and participate in the suppression of anti-nuclear autoimmunity as well as thrombotic microangiopathies caused by aggregated neutrophil extracellular traps. Since a systematic and direct comparison of the specific activities and properties of the secretory DNase1 family members is still missing, we expressed and purified recombinant murine DNase1 (rmDNase1), DNase1-like 2 (rmDNase1L2) and DNase1-like 3 (rmDNase1L3) using *Pichia pastoris*. Employing different strategies for optimizing culture and purification conditions, we achieved yields of pure protein between ~3 mg/l (rmDNase1L2 and rmDNase1L3) and ~9 mg/l (rmDNase1) expression medium. Furthermore, we established a procedure for post-expressional maturation of pre-mature DNase still bound to an unprocessed tri-N-glycosylated pro-peptide of the yeast α-mating factor. We analyzed glycosylation profiles and determined specific DNase activities by the hyperchromicity assay. Additionally, we evaluated substrate specificities under various conditions at equimolar DNase isoform concentrations by lambda DNA and chromatin digestion assays in the presence and absence of heparin and monomeric skeletal muscle α-actin. Our results suggest that due to its biochemical properties mDNase1L2 can be regarded as an evolutionary intermediate isoform of mDNase1 and mDNase1L3. Consequently, our data show that the secretory DNase1 family members complement each other to achieve optimal DNA degradation and chromatinolysis under a broad spectrum of biological conditions.

## Introduction

**Fig 1:**
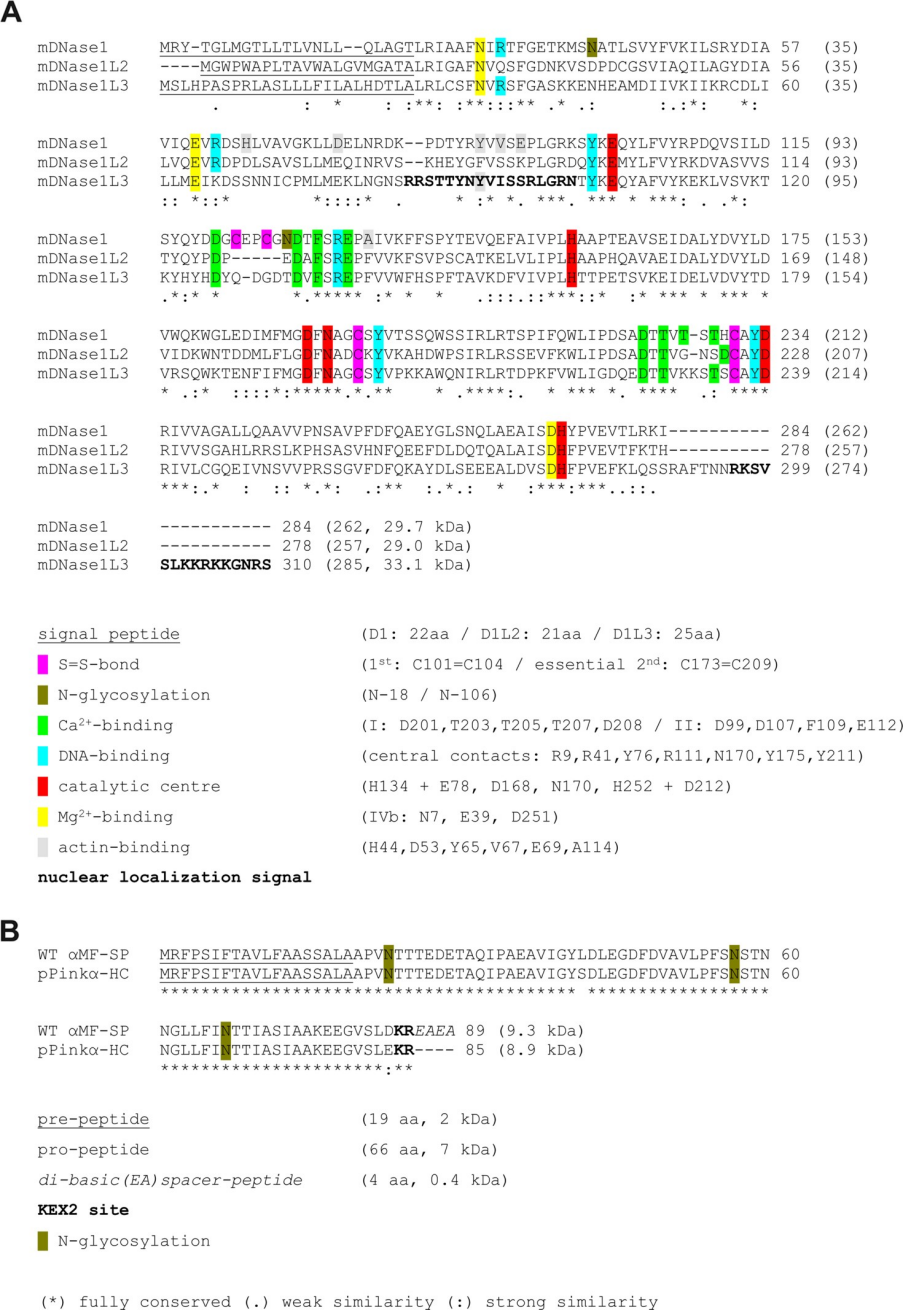
Protein alignment and illustration of functional domains and amino acids. (A) Alignment of the secretory mDNase1 family members by Clustal Omega: the multiple sequence alignment tool of EMBL-EBI. The numbering of essential amino acids refers to the mature mDNase1 protein, most of which were discovered for bovine and human DNase1. The length of the pre-mature and mature sequence is indicated in brackets. References: DNase1 disulfide bridges and Ca^2+^-binding [8, 9], DNase1 N-glycosylation [10], DNase1 DNA binding [11, 12], DNase1 catalytic center and Mg^2+^ binding [9, 11, 13, 14], DNase1 actin binding [15], DNase1L3 nuclear localization signals [16], (B) Wild-type *S*. *cerevisiae* α-mating factor signal prepro-peptide (αMF-SP) in comparison to that in pPinkα-HC of the *PichiaPink*^*TM*^ *pastoris* expression system [17].

Deoxyriboendonucleases play an important role in the intra- and extracellular clearance of DNA and chromatin during physiological processes such as cell differentiation, programmed cell death, necrosis and NETosis. The DNase1 family consists of DNase1 (also called DNase I) and its three relatives DNase1-like 1–3 which are involved in specific situations of the aforementioned processes [1]. Identified in 1946 and characterized subsequently, DNase1 is regarded as the first known member of the DNase1 family, while its highly homologous relatives were discovered only later [2]. They share common properties with DNase1 such as the N-terminal signal peptide (SP) for translation into the rough endoplasmic reticulum (rER), the divalent metal cation dependency and the endonucleolytic DNA cleavage activity generating 3´-hydroxyl and 5´-phosphate ends (Fig 1A) [3–7].

The different DNase1-like proteins display specific and partially overlapping expression profiles. Whereas DNase1L1 (also called DNL1L, Xib, DNase X [1]) is a muscle specific and membrane-bound protein, which supposedly builds a barrier against the endocytosis of foreign DNA [18–20], the other three nucleases are soluble secretory proteins, here referred to as the secretory DNase1 family members [7]. The expression pattern of DNase1L2 is restricted to keratinocytes, where it facilitates the removal of genomic DNA during their differentiation to hairs and nails and participates in the cornification process within the epidermis [21, 22]. The broadest expression profile displays DNase1, which occurs in almost all mammalian body fluids and secretions such as tear fluid, saliva, blood, urine, and seminal fluid [23, 24]. The majority of published studies indicate a suppressive function of DNase1 on anti-nuclear autoimmunity, which is typical for Systemic Lupus Erythematosus (SLE) and Lupus-nephritis, the most severe complication of SLE. Indeed, reduced levels of serum and renal DNase1, increased levels of DNase1 inhibitors and *DNASE1*/*Dnase1* gene mutations were described for humans and mice suffering from SLE [25–30]. It was noted, however, that the SLE phenotype found in the first described *Dnase1* KO mice of mixed 129xBL/6J F2 genetic background depended on additional pre-disposing genes and/or environmental factors, since it does not occur in pure 129 or C57BL/6J mice under specific pathogen-free conditions [31, 32]. Furthermore, the primary *Dnase1* KO mouse model harbored an aberrant knockdown mutation in the overlapping *Trap1* gene, which encodes for the mitochondrial chaperone Trap1/Hsp75 leading to a lack of detectable Trap1 protein [33, 34]. The influence of Trap1 deficiency on SLE is not clarified so far. On the one hand, newly generated *Dnase1* KO mice without *Trap1* impairment exhibit mild SLE-symptoms spontaneously in C57BL/6 mice [35]. This is in contradiction to the findings described for *Dnase1*^*-/-*^*/Trap1*^*m/m*^ mice of the same genetic background [32]. On the other hand, Trap1 deficiency in *Dnase1*^*-/-*^*/Trap1*^*m/m*^ mice leads to an increased stimulation of immune cells in the same inbred strain [34]. Thus, it remains unclear whether and which additional supporting factors are necessary for the induction of the SLE disease in DNase1 deficient mice, because the overall knowledge points to a multi-factorial origin of SLE [31]. In summary, most of the findings suggest that impaired DNase1 activity modifies anti-DNA autoimmunity albeit it might not be the sole cause. This view is supported by the fact that DNase1L3 (also called DNase γ, DNase Y, LS-DNase, nh-DNase [1]), which is expressed by macrophages as well as myeloid dendritic cells and occurs in the blood stream in parallel to DNase1 [36–38], is also regarded as a suppressor of anti-nuclear autoimmunity [38–40]. Both serum nucleases share complementary substrate specificities and act collectively in the dissolution of neutrophil extracellular traps (NETs) thereby preventing acute thrombotic microangiopathy during situations of neutrophilia [37, 41, 42]. Additionally, they dissolve genomic DNA within and expelled from primary and secondary necrotic cells [43–47]. Beside their role in extracellular and necrotic chromatin disposal, they were originally regarded as intracellular apoptotic nucleases [48, 49]. Indeed, overexpression of DNase1 in cell lines induces apoptosis accompanied by the typical internucleosomal DNA degradation [50]. However, the inhibitory effect of monomeric actin and the random chromatin cleavage pattern induced by DNase1 in the absence of proteases appear to make a sole involvement of DNase1 in apoptosis unlikely [51, 52]. These contradictory results might be explained by a recent finding demonstrating that overexpression of DNase1 induces a network of apoptotic endonucleases in transfected cells [53]. One of these endonucleases is DNase1L3, which possesses two nuclear localization signals (NLS, Fig 1A) and intrinsic internucleosomal cleavage capacity [16, 54].

So far, extensive research has been conducted to unravel the physiologic functions of single members of the DNase1 variants, however, a comparative quantitative analysis of their biochemical properties is still missing [1]. Only one study compared all four human DNase1 proteins after expression with His- and Myc-tags, which might, however, interfere with their natural nuclease properties [7]. Furthermore, due to the low amount of recombinant protein produced by transiently transformed eukaryotic cell lines, the specific activities and the DNA substrate cleavage efficiencies of the nucleases were not estimated [7].

In order to address this missing information, i.e. to characterize and compare the nucleases in a quantitative analysis, we expressed the secretory members of the mDNase1 family using the *PichiaPink™ pastoris* expression system [17]. In this system, the secretion of the recombinant protein is regulated by an expression cassette within vector pPinkα-HC, which consists of three constituents: the glycerol repressible and methanol inducible *AOX1* promoter, the N-terminal yeast α-mating factor signal prepro-peptide (αMF-SP) and the *CYC1* transcription terminator [55, 56]. The natural αMF-SP consists of a pre-peptide cleaved by a signal peptidase of the rER, a tri-N-glycosylated pro-peptide cleaved by kexin 2 (KEX2), a membrane-bound serine-type endopeptidase of the Golgi apparatus and a di-basic EA-spacer-peptide cleaved by the vacuolar dipeptidyl-aminopeptidase A Ste13. In contrast, the αMF-SP within pPinkα-HC lacks the spacer-peptide (Fig 1B). Advantages of this system are an easy and stable transformation with *Ade2* complementation for selection, a high cell density in the expression culture, which should lead to a high expression level, a eukaryotic protein processing including glycosylation allowing the purification of the recombinant protein from the expression culture supernatant (SN) in the absence of excessive foreign protein.

In this manuscript, we describe a systematic optimization of the expression of rmDNase1, rmDNase1L2 and rmDNase1L3 in *P*. *pastoris* by adapting the culture parameters and conditions. The adjustments ultimately led to an optimized processing of pre-mature αMF-DNases to the mature DNases. Purification procedures for the recombinant nucleases were established and the specific properties and activities under different ionic, pH-value and substrate conditions were quantified and compared.

## Materials and methods

### Accession numbers

Nucleotide sequence of the *Mus musculus Dnase1* (GenBank No. **NM_010061**), *Dnase12* (GenBank No. **NM_025718.3**) and *Dnase1l3* mRNA (GenBank No. **NM_007870.3**). Protein sequence of *Mus musculus* DNase1 (UniProtKB—**P49183**), DNase1L2 (UniProtKB–**Q9D1G0**), DNase1L3 (UniProtKB–**O55070**), and of *Saccharomyces cerevisiae* Mating factor alpha-1 (UniProtKB–**P01149**).

### Vector constructions for mDNase1

The cDNA encoding pre-mature renal mDNase1 was cloned as done and described in a previous study [37]. For expression in *P*. *pastoris*, the cDNA encoding mature mDNase1 was amplified using the primer pair Pich-D1-for/rev (Table 1) and cloned between the *Stu*I-*Kpn*I sites of pPinkα-HC (Invitrogen) in fusion to the N-terminal αMF-SP.

**Table 1:** Primers.

Cloning primers (Eurofins)		Cloned vector
		Cloning sites
D1L2-for2	5’-cagGAATTCtgcttttgtcATGGGTTGG-3’	pJET1.2/mDNase1L2
		blunt-end
D1L2-rev2	5’-CTGTGAacacatttaGCGGCCGCctta-3’	pDs/mDNase1L2
		EcoRI / NotI
Pich-D1L2-for	5’-CTGCGGATTGGAGCCTTCAATGTTCAGAG-3’	pPinkα-HC/mDNase1L2
Pich-D1L2-rev	5’-ATATCCTGAGGCCTGGTACC**TCA**GTGAG-3’	StuI / KpnI
AOX1-for2	5’-CCCCCTCGACGTCTAACATCCAAAGACGAAAGG-3’	pPinkα-HC/mDNase1L2-2x
		pPinkα-HC/mDNase1L2-KEX2
CYC1-rev2	5’-CCGCGGTGACGTCCGCAAATTAAAGCCTTCGAGCG-3’	pPinkα-HC/mDNase1L2-KEX2-dTM613
		pPinkα-HC/mDNase1L3-2x
		AatII
AOX1-for1	5’-GCCAGTGAATTGAGATCTAACATCCAAAG-3’	pPink-HC
AOX1-rev1	5’-GAAGGAAAAGGCCT**CGTTTCG**AATAATTAGTTG-3’	BglII / StuI
Pich-SP-D1L2-for	5’-CCT**ATG**GGTTGGCCCTGGGCCCCGCTGACAG-3’	pPink-HC/SP-mDNase1L2
Pich-SP-D1L2-rev	5’-ATATCCTGAGGCCTGGTACC**TCA**GTGAG-3’	StuI / KpnI
AOX1-for1’	5‘-GCCAGTGACTCGAGATCTAACATCCAAAG-3‘	pRS415/MeOH-KEX2
AOX1-rev1’	5‘-GAAGGAAACTGCAG**CGTTTCG**AATAATTAGTTG-3‘	XhoI / PstI
CYC1-for1	5‘-CCTTTGTGGATCCCATGTAATTAGTTATGTCAC-3‘	pRS415/MeOH-KEX2
CYC1-rev1	5‘-CGCGGATCGCGGCCGCAAATTAAAGCC-3‘	BamHI / NotI
KEX2-for2	5‘-CTAACCCATCTGCAG**ATG**AAAGTGAGG-3‘	pRS415/MeOH-KEX2-dUTR
KEX2-rev2	5‘-TTCTGTACGGATCCAA**TCA**CGATCGTCCGG-3‘	PstI / BamHI
KEX2-dTM613-for2	5‘-TTCGAAACGCTGCAG**ATG**aaagtgagg-3‘	pRS415/MeOH-KEX2-dTM613
KEX2-dTM613-rev2	5‘-TGGCCGGCCGGATCC**TCA**ACTTTC**TCA**ACG-3‘	PstI / BamHI
Pich-D1-for	5’-CTGAGAATTGCAGCCTTCAACATTCGG-3’	pPinkα-HC/mDNase1
Pich-D1-rev	5’-AACTGCTCAGGTACC**TCA**GATTTTTCTGAGTG-3’	StuI / KpnI
Pich-D1L3-for	5’-CTAAGGCTCTGCTCCTTCAATGTGAGG-3’	pPinkα-HC/mDNase1L3
Pich-D1L3-rev	5’-AAAGGCAGCGGGTACC**CTA**GGAGCG-3’	StuI / KpnI
sequencing primers (Eurofins)		
AOX1	5‘-GACTGGTTCCAATTGACAAGC-3‘	
CYC1	5‘-GCGTGAATGTAAGCGTGAC-3‘	

### Vector constructions for mDNase1L2 and KEX2

The cDNA encoding pre-mature mDNase1L2 was amplified by RT-PCR using the primer pair D1L2-for2/rev2 (Table 1) and cutaneous RNA of a C57BL/6 mouse. Having been cloned into pJET1.2-blunt (Fermentas), it was reamplified using the same primers and cloned between the *Eco*RI-*Not*I sites of pDsRed-N1 (Clontech). For expression of mDNase1L2 in *P*. *pastoris*, the cDNA encoding mature mDNase1L2 was amplified using the primer pair Pich-D1L2-for/rev (Table 1) and cloned between the *Stu*I-*Kpn*I sites of pPinkα-HC in fusion to the αMF-SP. For enhanced expression, the expression cassette of pPinkα-HC/mDNase1L2 was amplified using the primer pair AOX1-for2/CYC1-rev2 (Table 1) and cloned into the *Aat*II site of the same vector to generate pPinkα-HC/mDNase1L2-2x. In order to evaluate the expression of mDNase1L2 when tagged to its natural SP, the cDNA encoding pre-mature mDNase1L2 was amplified from pJET1.2/mDNase1L2 using the primer pair Pich-SP-D1L2-for/rev (Table 1) and cloned between the *Stu*I-*Kpn*I sites of pPink-HC. In order to generate pPink-HC, the αMF-SP of pPinkα-HC was eliminated by *Bgl*II/*Stu*I cleavage and the 5’-part of the expression cassette was replaced by an amplicon consisting of only the *AOX1* promoter, Kozak sequence and *Stu*I site using the primer pair AOX-for1/rev1 (Table 1). For co-expression of αMF-DNase1L2 and KEX2, we first modified pRS415/KEX2 (Addgene, #10996) by generating an expression cassette with *AOX1* promoter and *CYC1* terminator. First, the *AOX1* promoter was amplified from pPinkα-HC with primers AOX1-for1’/rev1’ (Table 1) and cloned between the *Xho*I-*Pst*I sites and secondly, the *CYC1* terminator was amplified with primers CYC1-for1/rev1 (Table 1) and cloned between the *Bam*HI-*Not*I sites of pRS415/KEX2 to reach of pRS415/MeOH-KEX2. Next, the 5’- and 3’-UTR of *KEX2* in vector pRS415/MeOH-KEX2 were eliminated by amplifying *KEX2-dUTR* with primers KEX2-for2/rev2 (Table 1) and cloning it between the *Pst*I-*Bam*HI sites of the same vector. For expression of secretory KEX2 lacking the transmembrane domain [57], a KEX2 variant coding for the first 613 aa was amplified using the primers KEX2-ΔTM613-for2/rev2 (Table 1) and cloned into the *Pst*I-*Bam*HI sites of vector pRS415/MeOH-KEX2 thereby replacing the full-length *KEX2* cDNA and generating pRS415/MeOH-KEX2-ΔTM613. In order to achieve co-expression of αMF-DNase1L2 and KEX2 or KEX2-ΔTM613, the expression cassettes of the KEX2 variants were amplified with primers AOX1-for2/CYC1-rev2 (Table 1) from the pRS415 vectors and cloned into the *Aat*II site of pPinkα-HC/mDNase1L2.

### Vector constructions for mDNase1L3

The cDNA encoding pre-mature splenic mDNase1L3 was cloned as done and described in a previous study except that the reverse primer was inadvertently specified incorrectly [37]. The reverse primer used in the previous study had the sequence 5’-GCGTGATACCTAGGAGCGATTG-3’. In order to express mDNase1L3 in *P*. *pastoris*, the cDNA encoding mature mDNase1L3 was amplified using the primer pair Pich-DN1L3-for/rev (Table 1) and cloned between the *Stu*I-*Kpn*I sites of pPinkα-HC in fusion to the N-terminal αMF-SP. For enhanced expression, the expression cassette of pPinkα-HC/mDNase1L3 was amplified using the primer pair AOX1-for2/CYC1-rev2 (Table 1) and cloned into the *Aat*II site of the same vector to generate pPinkα-HC/mDNase1L3-2x.

During revision of the manuscript, we noted retrospectively that the previous *mDnase1l3* cDNA preparation [37] contained two mutations each present in a separate cDNA molecule. First, an A to G substitution of nt-298 and secondly, an A to G substitution of nt-914 (CDS of NM_007870.3 encoding pre-mature mDNase1L3). Mutation nt-298 led to an exchange of Arg(R) to Gly(G)100 in the first NLS of natural pre-mature mDNase1L3 (position 75 without SP, Fig 1A) and was included in the previous study [37]. Mutation nt-914 was transferred into the present study during cloning the pPinkα-HC expression vector and causes an exchange of Lys(K) to Arg(R)305 (position 280 without SP) in the second NLS, which is located in the basic C-terminal tail of mDNase1L3 (Fig 1A). The mutation is chemically conservative (basic) and outside the regions that are responsible for catalytic activity and DNA or actin binding. In addition, also human DNase1L3 shows phylogenetic changes between K and R in the basic C-terminus [37]. Thus, the K305R exchange is highly likely to be devoid of significance for the results obtained in the present study.

### Transformation of electrocompetent *PichiaPink*^*TM*^ *pastoris*

For transformation, a glycerol stock of strain 4 of *PichiaPink*^*TM*^ *pastoris* (Invitrogen) was streaked on an YPD-plate (1% (w/v) yeast extract (Gibco-BRL), 2% (w/v) peptone (Foremedium), 2% (w/v) dextrose, 2.4% (w/v) agar (Carl Roth), and 50 μg/ml ampicillin (Sigma-Aldrich) and grown for 3–5 days at 28°C. As a starter culture, 10 ml YPD in a 100 ml Erlenmeyer baffled flask were inoculated with a colony, cultured at 28°C and 250 rpm for 1–2 days to OD_600_ 6. To generate electrocompetent cells, 100 ml YPD were inoculated to OD_600_ 0.2 with the starter culture and grown for 1–2 days at 28°C and 250 rpm in a 1 l Erlenmeyer baffled flask to OD_600_ 1.3–1.5. After each sedimentation step at 1,500g for 5 min at 4°C, the cells were washed twice in 250 and 50 ml of ice-cold water and thereafter resuspended in 10 and 0.3 ml of 1 M sorbitol, respectively.

For transformation 80 μl of fresh electrocompetent cells in 1 M sorbitol were mixed with 10 μg vector linearized with *Afl*II. The cells were transferred to an ice-cold 0.2 cm electroporation cuvette and electroporated at 1,500 V using the Electroporator 2510 (Eppendorf). After pulsing, 1 ml ice-cold YPDS (YPD with 1 M sorbitol) was added, the cells were mixed and incubated for 2 hours at 28°C without shaking. Aliquots of 200 μl were spread on Pichia adenine drop-out plates (PAD: 100 mM potassium phosphate buffer (PPB), pH 6.0, 0.2% (w/v) Kaiser’s SC-Ade mixture (Foremedium), 1.34% (w/v) yeast nitrogen base (YNB, Foremedium), 2% (w/v) dextrose, 2.4% (w/v) agar, and 50 μg/ml ampicillin) and incubated at 28°C until white colonies were formed. Single colonies were re-streaked on fresh plates. Starter cultures of colonies were prepared in 10 ml Synthetic Complete Dextrose adenine drop-out medium (SCD-Ade: 100 mM PPB, pH 6.0, 0.2% (w/v) Kaiser’s SC-Ade mixture, 2% (v/v) dextrose, 1.34% (w/v) YNB, 0.0004% (w/v) biotin (Sigma-Aldrich), and 50 μg/ml ampicillin) in a 100 ml Erlenmeyer baffled flask and cultured at 28°C and routinely 250 rpm to OD_600_ 6. For detection of transformed clones, 1 μl of a 1:10 starter culture dilution was used in a PCR specific for the expression cassette of pPinkα-HC (primers AOX1 and CYC1, Table 1). An aliquot of the PCR reaction was analyzed by 1.5% (w/v) Tris-borate/EDTA agarose gel electrophoresis with subsequent EtBr-staining. As a marker, the GeneRuler 1 kb DNA Ladder was used (ThermoFisher Scientific).

### Expression of recombinant proteins

For biomass production of *P*. *pastoris*, 500 ml growth medium were inoculated with a starter culture to OD_600_ 0.05 in a 5 l Erlenmeyer baffled flask. The growth culture was incubated overnight at 28–30°C under constant shaking at 250–300 rpm and grown to OD_600_ 6. Either Synthetic Complete Glycerol adenine drop-out (SCG-Ade) medium (SCD-Ade with 1% (v/v) glycerol instead of dextrose) or Buffered Glycerol-complex Medium (BMGY: 100 mM PPB, pH 6.0, 1% (w/v) yeast extract, 2% (w/v) peptone, 1% (v/v) glycerol, 1.34% (w/v) YNB, 0.0004% (w/v) biotin, and 50 μg/ml ampicillin) was used. After the growth culture, the cells were sedimented at 1,500 g, washed with 500 ml PBS, pH 6.0, for 30 min at RT, sedimented again, suspended in 50 ml expression medium to OD_600_ 60, and transferred to a 500 ml Erlenmeyer non-baffled flask. Either Synthetic Complete Methanol adenine drop-out (SCM-Ade) medium (SCG-Ade with 0.5% (v/v) methanol and 100 mM MES-buffer, pH 6.0, instead of glycerol and PPB) or Peptone Methanol medium (PM: 100 mM MES-buffer, pH 6.0, 0.5% (w/v) peptone, 0.5% (v/v) methanol, 1.34% (w/v) YNB, 0.0004% (w/v) biotin, and 50 μg/ml ampicillin) was used. The expression culture was incubated for 24 hours at 28–30°C under constant rotary shaking at 175 rpm. Subsequently, the cells were sedimented at 1,500 g and the cell-free SN was collected, filtrated through a 0.45 μm filter unit (Millipore) and supplemented with 1 mM NaN_3_.

### Diethylaminoethyl-cellulose anion-exchange chromatography

For purification of recombinant protein from SN or dialysate, DEAE-cellulose anion-exchange chromatography was employed. The SN or the dialysate diluted 1:4 with chromatography (C)-buffer (25 mM MES, pH 6.0) was run with 1 ml/min through a glass column loaded with 2 ml of activated and equilibrated DE53-cellulose (Whatman Biosystems). Afterwards, the column was washed with 20 ml C-buffer and eluted with portions of 10 ml C-buffer containing increasing amounts of 50–500 mM NaCl. Subsequently, 2 mM CaCl_2_ was added to the elution fractions to stabilize the 3D-structure of the DNases and to protect them against proteolysis [58, 59]. For maturation of pre-mature αMF-DNase1 prior to DEAE-cellulose chromatography, the SN was first dialyzed against 25 mM MES, pH 6.0, containing 2 mM CaCl_2_, 2 mM MgCl_2_ and 1 mM NaN_3_ using 30K filter units (Pierce) and incubated for 48 hours at 37°C. After DEAE-cellulose chromatography and prior to gel-filtration of rmDNase1, elution fraction E150 was concentrated with a 3K filter unit (Amicon). For purification of rmDNase1L2 and rmDNase1L3 by Heparin-Sepharose affinity chromatography, elution fraction E200 (rmDNase1L2) diluted 1:2 with Heparin-Sepharose C-buffer or the flow through (FT, rmDNase1L3) of the DEAE-cellulose chromatography was used.

### Heparin-Sepharose affinity chromatography

For purification of recombinant protein by Heparin-Sepharose affinity chromatography, 1 ml of Heparin-Sepharose (GE Healthcare) was loaded in a glass column and equilibrated with 20 ml chromatography (C)-buffer (25 mM MES, pH 6.0, 100 mM NaCl, 2 mM CaCl_2_, and 1 mM NaN_3_). Diluted E200 (rmDNase1L2) or the FT (rmDNase1L3) of the DEAE-cellulose anion-exchange chromatography was run through the column followed by washing with 10 ml C-buffer. Elution occurred with 7.5 ml of C-buffer containing 1.5 M NaCl. The eluate was diluted 1:2 with C-buffer without NaCl and concentrated with a 3K filter unit for subsequent gel-filtration.

### Gel-filtration

For gel-filtration, 80 ml swollen BioGel-P100 (Bio-Rad Laboratories) was loaded in a glass column and equilibrated with 2x storage buffer (50 mM MES, pH 6.0, 200 mM NaCl, 4 mM CaCl_2_, and 2 mM NaN_3_). Concentrated chromatography elution fractions were loaded on the gel bed and filtration was performed with 2x storage buffer. Fractions of 1 ml were collected and analyzed by SDS-PAGE and the hyperchromicity assay (HCA). Fractions containing mature nuclease were combined and concentrated to 2 mg/ml. The protein solution in 2x storage buffer was diluted 1:2 with glycerol and stored at -20°C. The storage buffer had been compiled based on different examples for commercially available DNase1 and was used to avoid extensive changes of the buffer compositions during the different steps of the purification procedure. Addition of proton donors to the storage or C-buffer was not tested, but does not seem to be recommended due to the two disulfide bridges in the native DNases of the DNase1 family.

### SDS-polyacrylamide gel electrophoresis (SDS-PAGE)

For SDS-PAGE according to Laemmli [60], 4% (v/v) collecting and 10–12% (v/v) resolving gels were prepared. Routinely, 0.5 ml SN was concentrated 1:10 with a 3K filter unit, mixed with 4x SDS gel-loading buffer, heated to 95°C for 5 min (rmDNase1L2: 70°C for 10 min) and half of the sample was employed in SDS-PAGE. Corresponding amounts of the sample from different steps of the purification were prepared for SDS-PAGE. Electrophoresis was carried out at 80–120 V using Tris/glycine buffer (25 mM Tris, 192 mM glycine, 0.1% (w/v) SDS, pH 8.7). As a protein marker, PageRuler™ Prestained Protein Ladder (ThermoFisher Scientific) or Cozy Prestained Protein Ladder (highQu) was used. After electrophoresis, the gels were stained with 0.5% (w/v) Coomassie brilliant blue G-250 (Serva) dissolved in 40% (v/v) methanol and 10% (v/v) acetic acid. For destaining, the gel was incubated in 10% (v/v) ethanol and 5% (v/v) acetic acid. Gels were photographed and analyzed using the ChemiDoc™ XRS+ System with the ImageLab 5.0 software (Bio-Rad Laboratories).

### Denaturing SDS-PAGE zymography (DPZ)

For DPZ, SDS-PAGE was performed with exception that the separating gels contained 100–200 μg/ml calf thymus DNA (Calbiochem). Samples, prepared as for SDS-PAGE, were diluted 1:20 to 1:200 in 1x SDS-sample buffer and 25 μl were loaded on the DPZ gel. For the detection of the native DNases, murine tissue homogenates were prepared as previously described [37]. Electrophoresis, washing, nuclease reaction, and detection within the gel were performed as previously described [37].

### Glycoprotein staining in SDS-polyacrylamide gels

Detection of glycoproteins by Periodic acid-Schiff staining in acrylamide gels was done according to Zacharius et al. [61]. In brief, gels were incubated in 12.5% (v/v) trichloracetic acid for 30 min, rinsed in distilled water and immersed in 1% (v/v) periodic acid for 50 min. Having been washed in several batches of distilled water for 60 min, the gels were immersed in fuchsin-sulfite stain for 50 min in the dark. Subsequently the gels were washed first 3x 10 min in 0.5% (w/v) metabisulfite and secondly in distilled water over night. Destaining and storage were achieved by transferring the gels to 5% (v/v) acetic acid.

### Hyperchromicity assay (HCA)

Specific activities of DNases were determined by HCA according to Kunitz [62]. The 1 ml reaction sample consisted of 50 μg/ml calf thymus DNA dissolved in 10 mM buffer, 0.1 mM CaCl_2_ and either 1 mM MgCl_2_, CoCl_2_ or MnCl_2_. The pH-values were adjusted using sodium acetate buffer for pH 5.0–5.5, MES-buffer for pH 6.0–6.5 and Tris-buffer for pH 7.0–9.0. Unless otherwise noted, we routinely used Tris-buffer, pH 7.0, containing 0.1 mM CaCl_2_ and 1 mM MnCl_2_ for measuring the specific activities of expression or purification samples. The absorbance at 260 nm was recorded for 1 min at RT in a quartz cuvette with 1 cm path length (Beckman DU640). One unit of nuclease is defined by an increase in absorbance of 0.001 per minute under the given conditions. For the calculation of equimolar amounts of the nucleases the molecular mass (MM) of the mature protein was used (Fig 1A). For actin inhibition the respective DNase and monomeric skeletal muscle α-actin were incubated in 50 μl of 10 mM buffer (as indicated in the figure captions) containing 0.1 mM CaCl_2_ and 1% (v/v) protease inhibitor cocktail (PIC, Sigma-Aldrich) for 10 min on ice. Afterwards, the sample was completely employed in the HCA.

### Lambda DNA digestion assay

Activity of DNases towards protein-free DNA was determined in an assay containing 25 μg/ml phage lambda DNA (ThermoScientific) dissolved in 10 mM buffer in the presence of 2 mM CaCl_2_ and either 2 mM MgCl_2_ or MnCl_2_ for 30 min at 37°C. The pH-values were adjusted using sodium acetate buffer for pH 4.0–5.0, MES-buffer for pH 6.0 and Tris-buffer for pH 7.0–9.0. The reaction was stopped by addition of 10 mM EDTA and heating to 65°C for 5 min. An aliquot was analyzed by 1.5% (w/v) Tris-borate/EDTA agarose gel electrophoresis with subsequent EtBr-staining. In some experiments, the effect of 125 units/ml heparin (B. Braun Melsungen AG) on the DNase activities was investigated.

### Chromatin digestion assay

Activity of DNases towards protein-complexed DNA (chromatin) was determined in a 200 μl reaction with 10^5^ nuclei of murine NIH-3T3 fibroblasts dissolved in 10 mM Tris-buffer, pH 7.5, 50 mM NaCl, 2 mM CaCl_2_, 2 mM MgCl_2_, and 1% (v/v) PIC. After incubation at 37°C for 18 hours, nuclear DNA was isolated with the QIAamp DNA Blood Mini Kit (Qiagen), and half of the DNA was analyzed by 1.5% (w/v) Tris-borate/EDTA agarose gel electrophoresis with subsequent EtBr-staining. In some experiments, the effect of 500 units/ml heparin on the DNase activities was investigated. Cell nuclei were isolated according to Stolzenberg et al. [63].

### Actin polymerization assay

Actin polymerization was measured by following the increase in fluorescence intensity of 0.5 μM pyrenyl-labeled monomeric skeletal muscle α-actin (pyrene-actin) added to 5 μM non-labeled monomeric α-actin [64] over a period of 20 to 30 min after addition of 2 mM MgCl_2_ using a Perkin-Elmer spectrofluorometer at excitation and emission wavelengths of 365 and 385 nm, respectively [65]. For the determination of the effect of the DNase variants on actin polymerization the respective DNase was pre-incubated for 10 min with equimolar monomeric α-actin at RT in reaction buffer (10 mM Hepes-buffer, pH 7.5, 0.1 mM CaCl_2_ and 0.2 mM ATP) before the addition of 2 mM MgCl_2_. Rabbit skeletal muscle α-actin was prepared from acetone powder as described [66].

## Results

### Establishment of rmDNase1L2 expression

**Fig 2:**
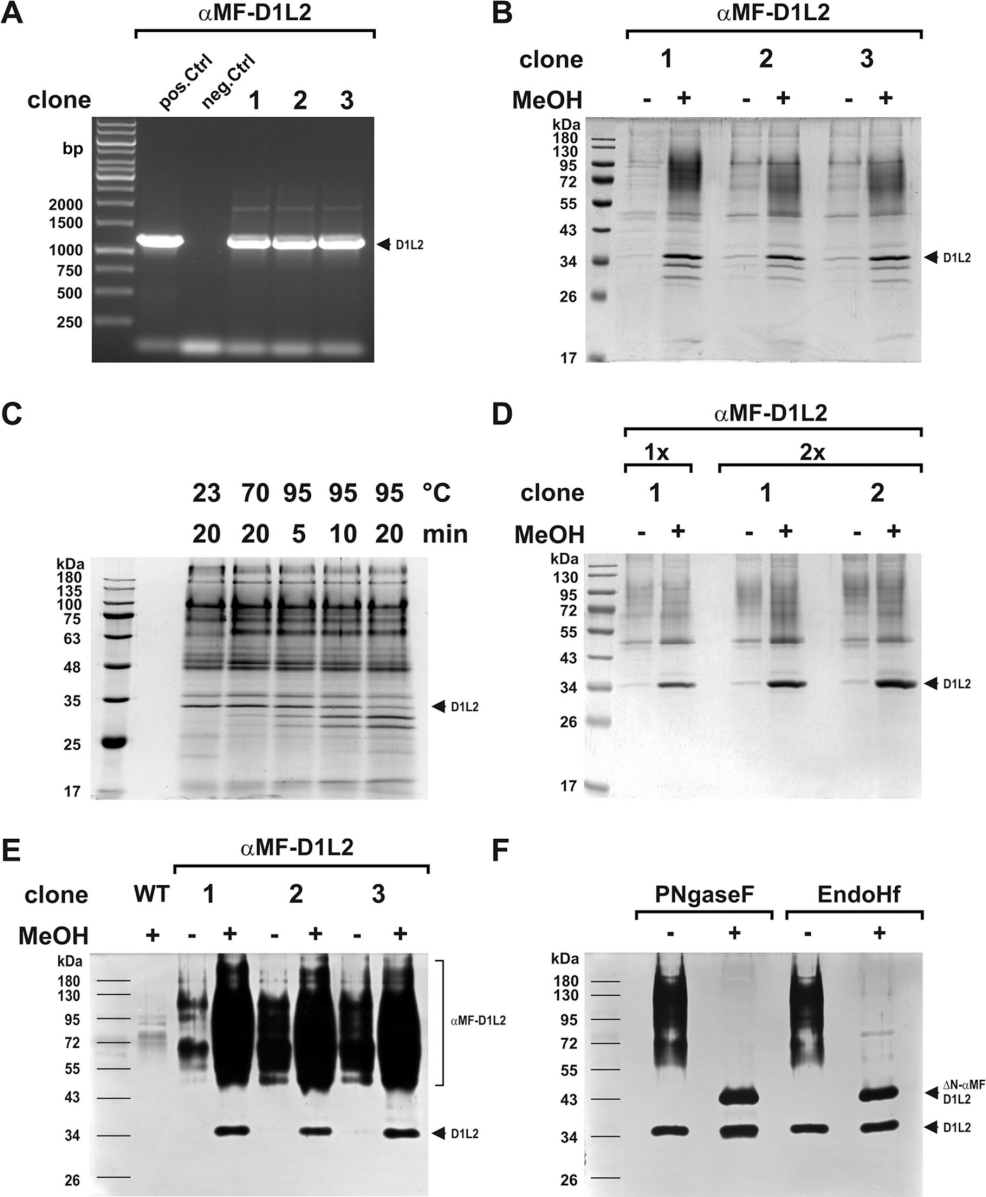
Establishment of rmDNase1L2 expression. (A) Detection of a single transgene integration for αMF-DNase1L2 (αMF-D1L2) in three transformed clones by expression cassette specific PCR (αMF-D1L2: 1197 bp, pos. Ctrl: vector, neg. Ctrl: water, marker: GeneRuler 1 kb DNA Ladder). (B) Detection of mature rmDNase1L2 (D1L2, ~34 kDa vs calculated 29 kDa) in the SN of MeOH-induced clones by SDS-PAGE and Coomassie staining. Note the heat-dependent instability of rmDNase1L2 in Laemmli sample buffer [60] as shown in (C). (D) Enhanced expression level of mature rmDNase1L2 by doubling the expression cassette for αMF-DNase1L2 in the transformed vector (1x vs 2x). (E) Functionality of rmDNase1L2 as verified by DPZ. Note the high MM nuclease smear with its lowest band at ~48 kDa representing pre-mature rmDNase1L2 that is still fused to the hyper-tri-N-mannosylated pro-peptide of αMF-SP (αMF-D1L2). (F) De-N-glycosylation (ΔN) by PNgaseF or EndoHf confers the high MM nuclease smear to a single ΔN-αMF-DNase1L2 nuclease band (~41 kDa) in DPZ (ΔN-αMF-D1L2: pre-mature rmDNase1L2 of ~34 kDa in fusion to the de-N-glycosylated 7 kDa pro-peptide of αMF-SP, Fig 1A and 1B). Marker: PageRuler™ Prestained Protein Ladder in (B, D, E, and F) and Cozy Prestained Protein Ladder in (C).

Recombinant expression of the secretory murine DNases was carried out with the *PichiaPink*^*TM*^ *pastoris* system using of αMF-SP [17]. We started with mDNase1L2, which as pre-mature form consists of 278 amino acids (aa). After the cleavage of the N-terminal SP, mature mDNase1L2 consists of 257 aa with a calculated MM of ~29 kDa (Fig 1A and Table 2). For expression of rmDNase1L2, the cDNA encoding the mature nuclease was cloned in fusion to the αMF-SP (αMF-mDNase1L2) generating pPinkα-HC/mDNase1L2. Strain 4 of *PichiaPink*^*TM*^ *pastoris*, which displays reduced proteolysis of secreted recombinant proteins (i.e. a lack of proteinase A, proteinase B and carboxypeptidase Y due to a double knockout of *pep4* and *prb1*) [17], was transformed and transgenic clones were isolated (Fig 2A). Before optimization, SCG-Ade growth medium was used for biomass production and SCM-Ade for transgene expression. Cultures were incubated at 28–30°C in a shaking incubator at 250–300 rpm (growth, baffled flasks) or 175 rpm (expression, non-baffled flasks). In order to reach optimal ventilation, the ratio of culture and flask volume was always 1:10. Expression SN was concentrated and analyzed by SDS-PAGE and/or DPZ.

**Table 2:** Biochemical features of the secretory murine DNase1 family members.

Feature		mDNase1	mDNase1L2	mDNase1L3^∞^
signal peptide *°		22 aa	21 aa	25 aa
mature protein *°		262 aa / 29.7 kDa	257 aa / 29.0 kDa	285 aa/ 33.1 kDa
pI-value °		4.69	4.95	8.85
aa-sequence identity / similarity ^#^	mDNase1	100%	53.4% / 70.2%	45.1% / 58.4%
	mDNase1L2	53.4% / 70.2%	100%	41.5% / 57.5%
	mDNase1L3^∞^	45.1% / 58.4%	41.5% / 57.5%	100%
protein-free DNA-cleavage by HCA: pH-optimum specific activity [kU/nmol]	Ca^2+^ / Mg^2+^	**pH 7.5**	pH 6.5	pH 8.0
		**15.6±2.5**	2.8±0.3	1.8±0.1
	Ca^2+^ / Co^2+^	pH 7.0	pH 7.0	pH 7.0
		7.7±0.6	1.4±0.3	1.6±0.1
	Ca^2+^ / Mn^2+^	pH 8.0	**pH 7.5**	**pH 7.5**
		13.9±0.6	**5.0±0.3**	**5.5±0.9**
glycosylation*		N-18 / N-106	-	-
monomeric α-actin interaction	HCA (Ca^2+^ / Mg^2+^)	~50% inhibition (equimolar)	-	-
chromatin cleavage		random	bi-modular	internucleosomal
heparin interaction		-	binding	binding
	HCA	-	inhibition	inhibition
	λ-DNA assay	-	inhibition	inhibition
	chromatin assay	activation	switch to random cleavage	inhibition

^#^ EMBOSS Needle program of the European Molecular Biology Laboratory.

* UniProt Knowledgebase (UniProtKB).

° ExPASy Compute pI/Mw tool from the Swiss Institute for Bioinformatics.

^**∞**^ K305R (position 280 without SP, Fig 1A).

Interestingly, the SDS-PAGE showed that the SN of transgenic clones contained a prominent protein of ~34 instead of 29 kDa as calculated for mature mDNase1L2 (Fig 2B). This implies either post-translational modification or an aberrant mobility of rmDNase1L2 in SDS-PAGE. Additionally, proteins of 17–34 kDa were detectable, which resembled a degradation pattern (Fig 2B). However, the addition of protease inhibitors during expression and sample preparation did not prevent the hypothesized degradation (S1 Fig). Instead, we found that rmDNase1L2 is vulnerable to heat dependent degradation in acidic loading buffer (Fig 2C) as described for other proteins [67, 68]. To prevent degradation, rmDNase1L2 samples were heated for 10 min at only 70°C. Since the expression level of mature rmDNase1L2 appeared low, we doubled the expression cassette in the vector and observed an increased expression in transformed clones (Fig 2D).

Next, we evaluated whether the MeOH-induced protein pattern entirely resulted from rmDNase1L2 expression and performed DPZ. As expected, wild-type (WT) *P*. *pastoris* did not secrete appreciable nucleases (Fig 2E). In contrast, all clones transformed displayed excessive nuclease activity in the SN after induction. Most of the activity appeared as a smear of high MM migrating above 48 kDa (lowest detectable band of the smear). On the other hand, the assigned mature rmDNase1L2 detectable in SDS-PAGE (Fig 2D) appeared again as a distinct band of ~34 kDa in DPZ (Fig 2E). This is consistent with a functional expression of rmDNase1L2 in *P*. *pastoris*. To evaluate a plausible post-translational modification, N-glycosylation was investigated. Notably, de-N-glycosylation by PNGaseF or EndoHf resulted in the disappearance of the high MM smear in DPZ and transformed it into a single nuclease band of ~41 kDa (Fig 2F). In contrast, the mature rmDNase1L2 of ~34 kDa was unaffected by this treatment as it is not N-glycosylated. After de-N-glycosylation, the difference in MM between both detectable nuclease bands was ~7 kDa, which fits to the MM of the de-N-glycosylated pro-peptide of αMF-SP (14 and 7 kDa with and without glycosylation, respectively [69], Fig 1B). In summary, ~50% of rmDNase1L2 expressed is pre-mature and still contains an extensively N-glycosylated pro-peptide (Fig 2E). Because processing of αMF-DNase1L2 occurs inefficiently, we replaced the αMF-SP in the expression cassette by the SP of native mDNase1L2. Unfortunately, expression failed pointing to a functional loss of this mammalian SP in *P*. *pastoris* (S2 Fig).

**Fig 3:**
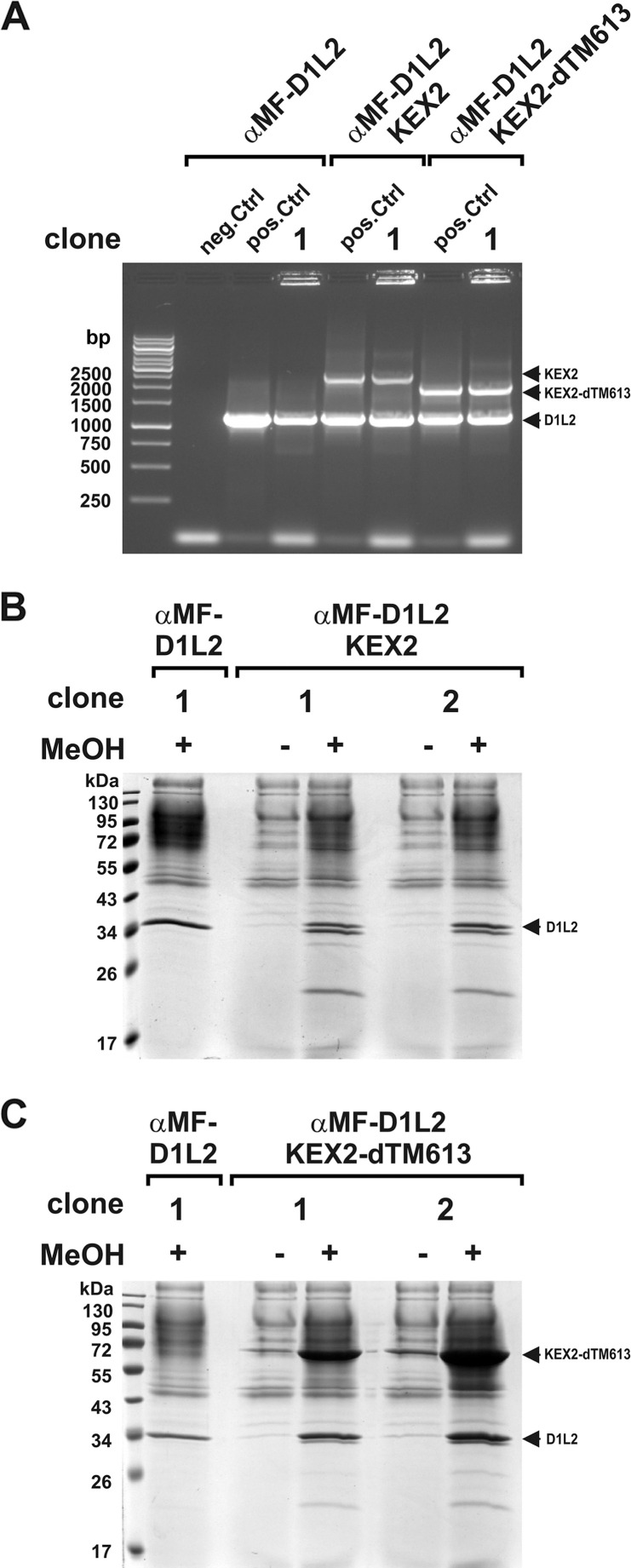
Co-expression of αMF-DNase1L2 with KEX2 or soluble KEX2-ΔTM613. (A) Detection of the different transgene integrations in transformed clones by expression cassette specific PCR (αMF-D1L2: 1197 bp, KEX2: 2597 bp, KEX2-ΔTM613: 2034 bp, pos.Ctrl: vector, neg.Ctrl: water, marker: GeneRuler 1 kb DNA Ladder). (B) Detection of rmDNase1L2 (D1L2) in the SN of MeOH-induced clones by SDS-PAGE and Coomassie staining reveals rmDNase1L2 degradation in clones co-expressing Golgi-located full-length KEX2. (C) Degradation of rmDNase11L2 also occurs in the presence of soluble truncated KEX2-ΔTM613. Marker in (B, C): PageRuler™ Prestained Protein Ladder.

In a further attempt, we tried to overcome the inefficient pro-peptide cleavage by co-expressing KEX2. Therefore, chimeric expression vectors for αMF-DNase1L2 and either Golgi-located full-length or truncated soluble KEX2 lacking the transmembrane domain (KEX2-ΔTM613) were cloned [57]. Despite successful transformation (Fig 3A), we found signs of proteolysis of mature rmDNase1L2 instead of an increased expression level (Fig 3B and 3C).

### Optimization of rmDNase1L2 expression

**Fig 4:**
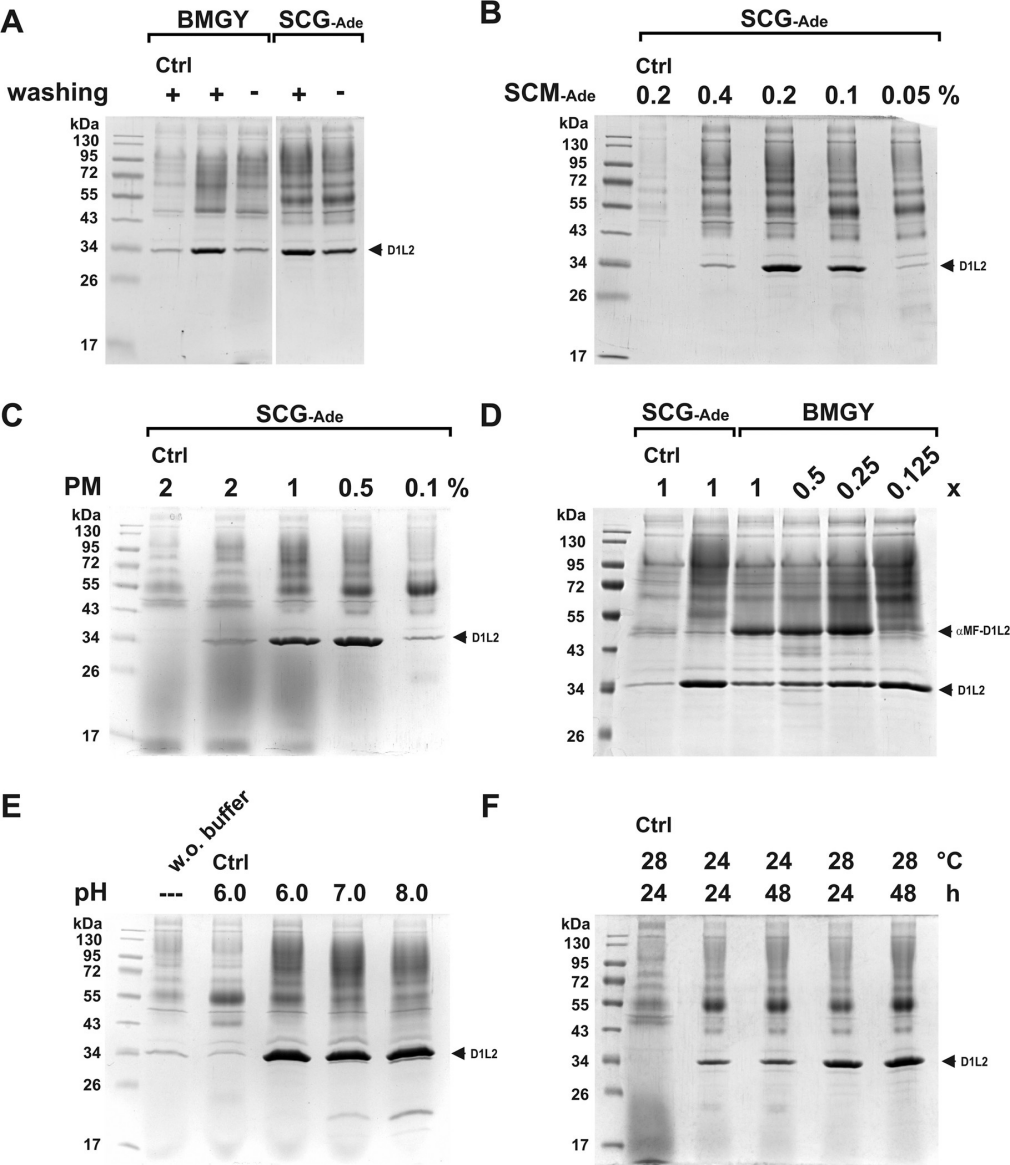
Optimization of rmDNase1L2 expression. (A) Washing cells with PBS, pH 6.0, between growth and expression (MeOH containing SCM-Ade medium) increased the expression of rmDNase1L2 (D1L2) independently of the growth medium (BMGY or SCG-Ade). (B) Dependence of rmDNase1L2 expression on the concentration of Kaiser`s synthetic complete adenine drop-out mixture in the SCM-Ade expression medium. SCG-Ade growth medium was used. (C) Replacement of SCM-Ade by peptone methanol (PM) expression medium enhances the expression of rmDNase1L2 at an optimal concentration of 0.5% (w/v) peptone. (D) Dependence of the expression level and processing of pre-mature αMF-DNase1L2 (αMF-D1L2) to mature rmDNase1L2 on the nutrient content of the growth medium. Compared are 1x SCG-Ade (0.2% (w/v) Kaiser’s SC-Ade) and BMGY medium (1x: 1% (w/v) yeast extract, 2% (w/v) peptone, YP) with varying YP-concentrations. (E, F) Expression of rmDNase1L2 using 1x SCG-Ade growth and PM-expression medium with 0.5% (w/v) peptone. (E) Stability of mature rmDNase1L2 depends on the pH-value of the expression medium (0.1 M potassium phosphate buffer). (F) Influence of the temperature and duration of the expression culture on the expression level of mature rmDNase1L2. In all experiments, the basal expression pattern of *P*. *pastoris* transgenic for *mDnase1l2* without MeOH induction was evaluated in parallel (Ctrl). Marker in (A-F): PageRuler™ Prestained Protein Ladder.

**Fig 5:**
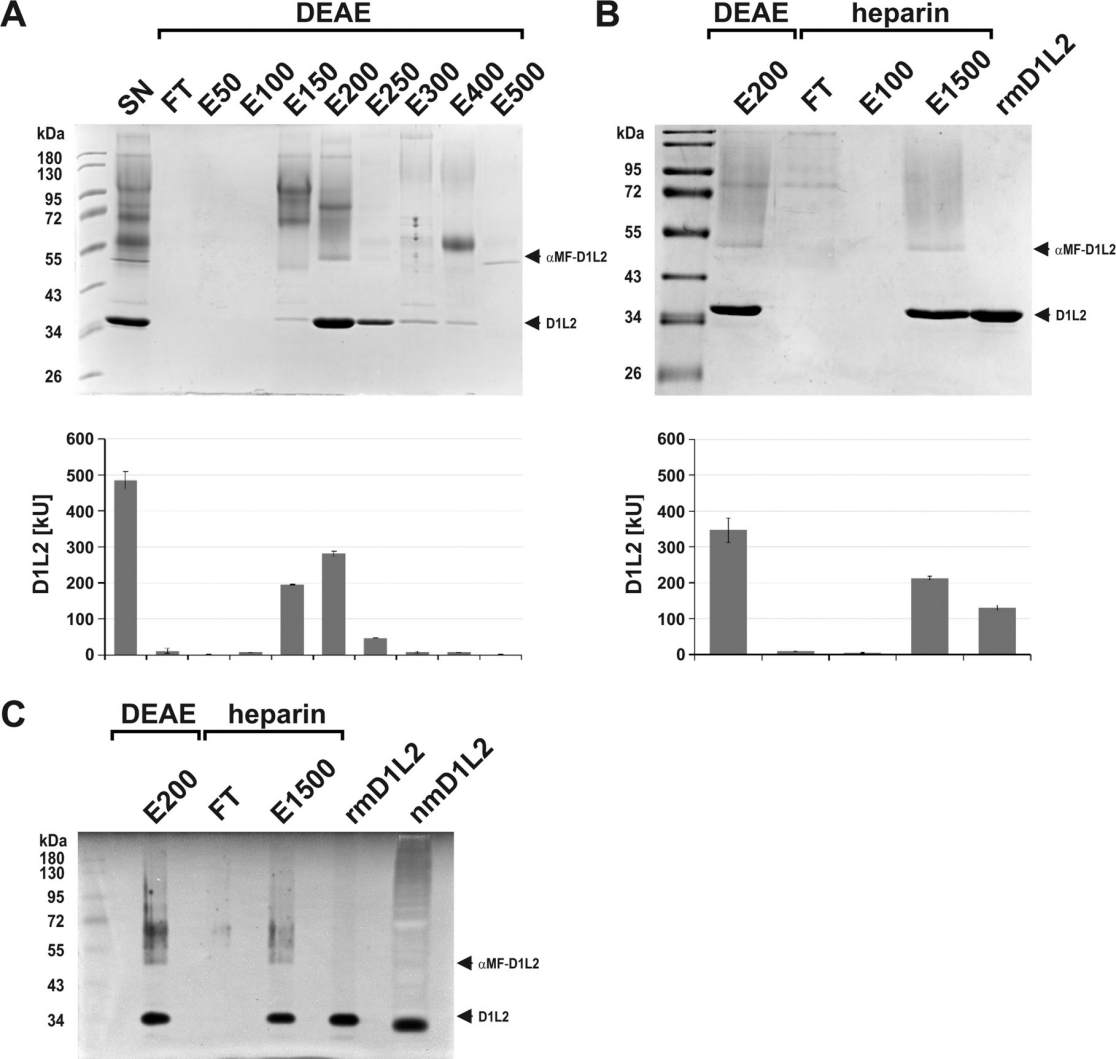
Purification of rmDNase1L2 from culture supernatant. (A) DEAE-cellulose anion-exchange chromatography of filtrated and diluted SN. (FT: flow through, E50-500: elution fraction at 50–500 mM NaCl). (B) Purification of mature rmDNase1L2 (rmD1L2, D1L2) from DEAE elution fraction E200 by Heparin-Sepharose affinity chromatography. Final purification of mature rmDNase1L2 from E1500 was done by gel-filtration (last lane). (A, B) Top image: Coomassie-stained SDS-gel, lower image: absolute activity of the samples measured by HCA. (C) Successful separation of mature rmDNase1L2 from pre-mature αMF-DNase1L2 (αMF-D1L2) as shown by DPZ. The final pure rmDNase1L2 protein co-migrates with its native form (nmD1L2) present in a cutaneous tissue homogenate of a *Dnase1/Dnase1l3* double KO mouse (20 μg protein loaded). Marker in (A-C): PageRuler™ Prestained Protein Ladder.

In general, we followed the instructions of the Invitrogen user manual for the *PichiaPink*^*TM*^ *pastoris* system [17]. However, several factors are critical for recombinant protein expression in *P*. *pastoris* [17, 70]. Routinely, we started our experiments using SCG-Ade growth and SCM-Ade expression medium to maintain the selection pressure. Subsequently, parameters were adapted to enhance expression as the following account shows: We found that washing cells with PBS prior to the induction of gene expression increased the expression level of rmDNase1L2 (Fig 4A). Since this increase might have been caused by a starvation effect, we varied the nutrient content during expression. However, changing the standard concentration of 0.2% (w/v) Kaiser’s SC-Ade mixture in the SCM-Ade medium did not further increase protein expression (Fig 4B). Therefore, we tried to change the type of expression medium to reach this goal. Some investigators and the supplier propose Buffered Methanol-complex Medium (BMMY) with 1% (w/v) yeast extract and 2% (w/v) peptone (1x YP). Because BMMY contains high amounts of pigments, which are difficult to remove during purification, we reduced their content by omitting yeast extract and varying the amount of peptone (Peptone Methanol (PM) medium). At a concentration of 0.5% (w/v) peptone, PM was found to be even more efficient than the SCM-Ade expression medium (Fig 4B and 4C) and was therefore applied in all further experiments. Notably, no additional increase of expression was achieved using BMGY instead of SCG-Ade growth medium in combination with PM (Fig 4D). Instead, we observed that processing of pre-mature αMF-DNase1L2 (~48 kDa) to mature rmDNase1L2 (~34 kDa) depended on the amount of YP in the BMGY growth medium. Lowering its concentration to 0.125x was shown to be equivalent to SCG-Ade (Fig 4D). Further factors for an optimal production of mature rmDNase1L2 were a slightly acidic pH-value in the expression medium (pH 6.0) as well as an incubation temperature around 28°C (Fig 4E and 4F). The more alkaline the pH-value the more degradation of rmDNase1L2 occurred (Fig 4E). For growth, we routinely used potassium phosphate buffer, pH 6.0, whereas for expression MES, pH 6.0, was preferred, since phosphate might interfere with the nuclease activities. Phosphate or MES-buffer behaved equivalently in the expression experiments (compare Fig 4E with Fig 5A). Notably, an increased incubation time had no further promoting effect on expression (Fig 4F).

In summary, for optimal expression we grew *P*. *pastoris* transgenic for *mDnase1l2* at 28°C and 250–300 rpm to OD_600_ 6 using standard SCG-Ade medium, pH 6.0, washed the cells with PBS, pH 6.0, for 30 min at RT and finally performed expression with PM medium, pH 6.0, containing 0.5% (w/v) peptone at a culture density of OD_600_ 60 at 28°C and 175 rpm for 24 hours.

### Purification of rmDNase1L2 from culture supernatant

Mature mDNase1L2 has a pI-value of 4.95 with a charge of -8.2 at pH 6.0 (Table 2). Therefore, we chose DEAE-cellulose anion-exchange chromatography for its concentration from the cell-free SN of the expression cultures. For all preparation steps, the buffer of the expression medium (MES, pH 6.0) was maintained to avoid destabilization of rmDNase1L2. After dilution, the SN was loaded onto an equilibrated DEAE column. Performing SDS-PAGE and HCA, we found that rmDNase1L2 bound completely to the resin (Fig 5A). Stepwise elution was performed with buffer containing increasing concentrations of 50–500 mM NaCl. The major part of mature rmDNase1L2 eluted at 200 mM (E200), whereas most of pre-mature αMF-DNase1L2 and further proteins of high MM eluted at 150 mM NaCl (Fig 5A). Since elution fraction E200 still included contaminants of high MM (Fig 5A), we tested further purification methods and found that rmDNase1L2 of diluted E200 bound completely to equilibrated Heparin-Sepharose comparable to rmDNase1L3 [37] (Fig 5B). The FT contained the high MM contaminants and almost no nuclease activity. Elution occurred at 1.5 M NaCl with an efficiency of ~62%. However, E1500 still contained nuclease activity of high MM (≥48 kDa) representing pre-mature αMF-DNase1L2 (Fig 5C).

In order to achieve final purification we concentrated diluted E1500, performed gel-filtration and observed two elution peaks. The fractions of the second peak were concentrated, diluted by addition of glycerol and an aliquot was tested by DPZ. It contained pure mature rmDNase1L2 co-migrating with native mDNase1L2 present in a cutaneous tissue homogenate (Fig 5C). Purified rmDNase1L2 stored at -20°C was stable for at least one month as determined by HCA (4.96±0.03 versus 4.12±0.12 kU/nmol). About 26% of the nuclease activity present in the SN was isolated as mature rmDNase1L2 with a yield of maximal 3 mg/l expression culture.

### Establishment and optimization of rmDNase1 expression

**Fig 6:**
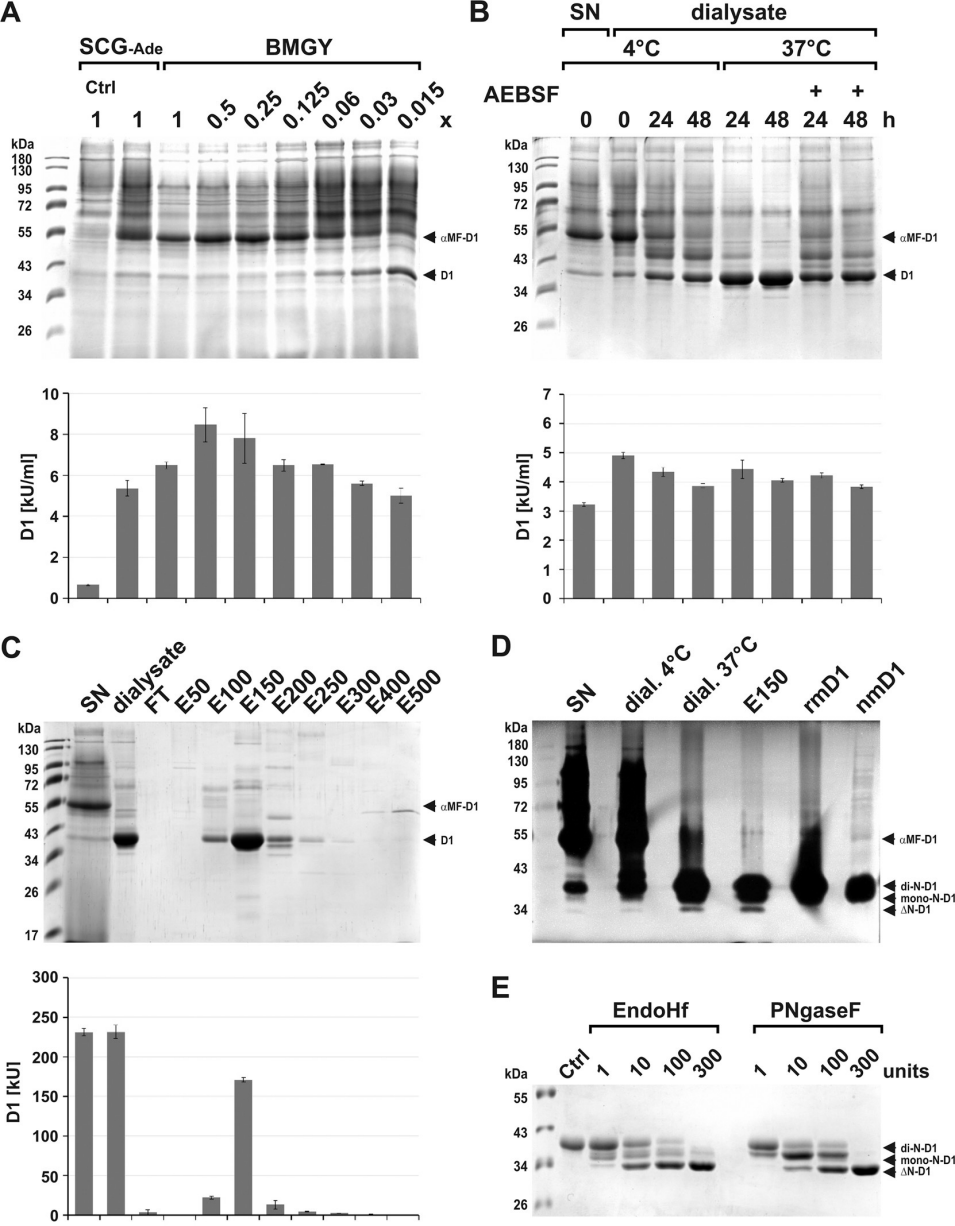
Establishment of expression and purification of rmDNase1 from culture supernatant. (A) Expression and processing of pre-mature αMF-DNase1 (αMF-D1, ~51 kDa) to mature rmDNase1 (D1, ~37 kDa) depends on the choice and nutrient content (1x BMGY: 1% yeast extract, 2% (w/v) peptone) of the growth medium (PM expression medium was used). (B) Temperature, time and protease dependent processing of pre-mature αMF-DNase1 to mature rmDNase1 in dialyzed SN in the presence and absence of the protease inhibitor AEBSF. (C) Purification of mature rmDNase1 from dialyzed and processed SN by DEAE-cellulose anion-exchange chromatography (FT: flow through, E50-500: elution fraction at 50–500 mM NaCl). (A-C) Top image: Coomassie-stained SDS-gel, lower image: specific activity of the samples measured by HCA. (D) Successful separation of mature rmDNase1 from pre-mature αMF-DNase1 as shown by DPZ. Purified rmDNase1 co-migrates with native mDNase1 (nmD1) present in a lacrimal tissue homogenate of a WT mouse (50 ng protein loaded). (E) Analysis of de-N-glycosylation by EndoHf and PNGaseF reveals di-N-glycosylation (mannosylation) of rmDNase1 by DPZ. (rmDNase1: di-N-glycosylated ~ 37 kDa, mono-N-glycosylated ~35 kDa and Δ-N-glycosylated ~ 33 kDa). Marker: PageRuler™ Prestained Protein Ladder.

Next, we applied the protocol for expression of rmDNase1L2 to mDNase1. Mature mDNase1 consists of 262 aa with a calculated MM of ~30 kDa excluding any N-linked glycosylation (Fig 1A and Table 2). For expression, the cDNA for mature mDNase1 cloned in fusion to αMF-SP in pPinkα-HC was used to transform strain 4 of *PichiaPink*^*TM*^ *pastoris* and positive clones were selected. We started with SCG-Ade growth and PM expression medium and also left other parameters identical to those for rmDNase1L2. In contrast to rmDNase1L2, a single expression cassette sufficed to reach elevated rmDNase1 activity. However, only pre-mature αMF-DNase1 (~51 kDa) was detectable (Fig 6A). Since an impact of the growth medium on processing of pre-mature αMF-DNase1L2 existed (Fig 4D), we tested BMGY with reduced YP and found an optimized processing of pre-mature to mature rmDNase1 (~37 kDa) at a concentration of 0.015x. Regarding the rmDNase1 activity however, BMGY with a higher YP content was more effective (Fig 6A). Therefore, we chose BMGY with 0.25x YP in combination with PM expression medium for the next experiments. A further reason for this was found by chance: Incubation of SN for 24–48 hours at 37°C led to the trimming of the αMF-SP with conversion of premature to mature rmDNase1. This process depended on the prior dialysis of the SN and occurred without loss of function as proved by HCA (Fig 6B). Trimming was mediated by a protease(s) in the SN, since it was retarded by addition of the protease inhibitor AEBSF (Figs 6B and S3). The protease concentration depended on the growth conditions and was greatest at 30°C and during shaking at 300 rpm for maximal ventilation (S3 Fig). However, protease release occurred during expression, since cells were prior washed with PBS.

### Purification of rmDNase1 from culture supernatant

Mature mDNase1 has a pI-value of 4.69 with a charge of -10.2 at pH 6.0 (Table 2). Thus, having trimmed the αMF-SP in the dialyzed SN, we performed DEAE-cellulose anion-exchange chromatography analogous to rmDNase1L2. Binding was complete and elution occurred at 150 mM NaCl with an efficiency of ~74% and high purity (Fig 6C). In order to remove minor contaminants (Fig 6C) we subsequently performed gel-filtration identical to rmDNase1L2. Fractions with pure mature rmDNase1 were concentrated and samples were analyzed by DPZ. Our results show that pre-mature αMF-DNase1 was transformed entirely into mature rmDNase1, which was subsequently purified to homogeneity. It co-migrated with native mDNase1 present in a murine lacrimal tissue homogenate (Fig 6D). Similar to native mDNase1 from the parotid gland and fibroblastic rmDNase1 [37], the rmDNase1 produced by *P*. *pastoris* possessed two N-glycosylations, which, in contrast to mDNase1 expressed by mammalian cells, were entirely of the high-mannose type (Fig 6E). Analogous to rmDNase1L2, rmDNase1 stored at -20°C was stable for at least one month as determined by HCA (5.09±0.11 versus 4.12±0.05 kU/nmol). About ~35% of the nuclease activity present in the SN was isolated as pure mature rmDNase1 with a maximal yield of 9 mg/l expression culture.

### Establishment and optimization of rmDNase1L3 expression

**Fig 7:**
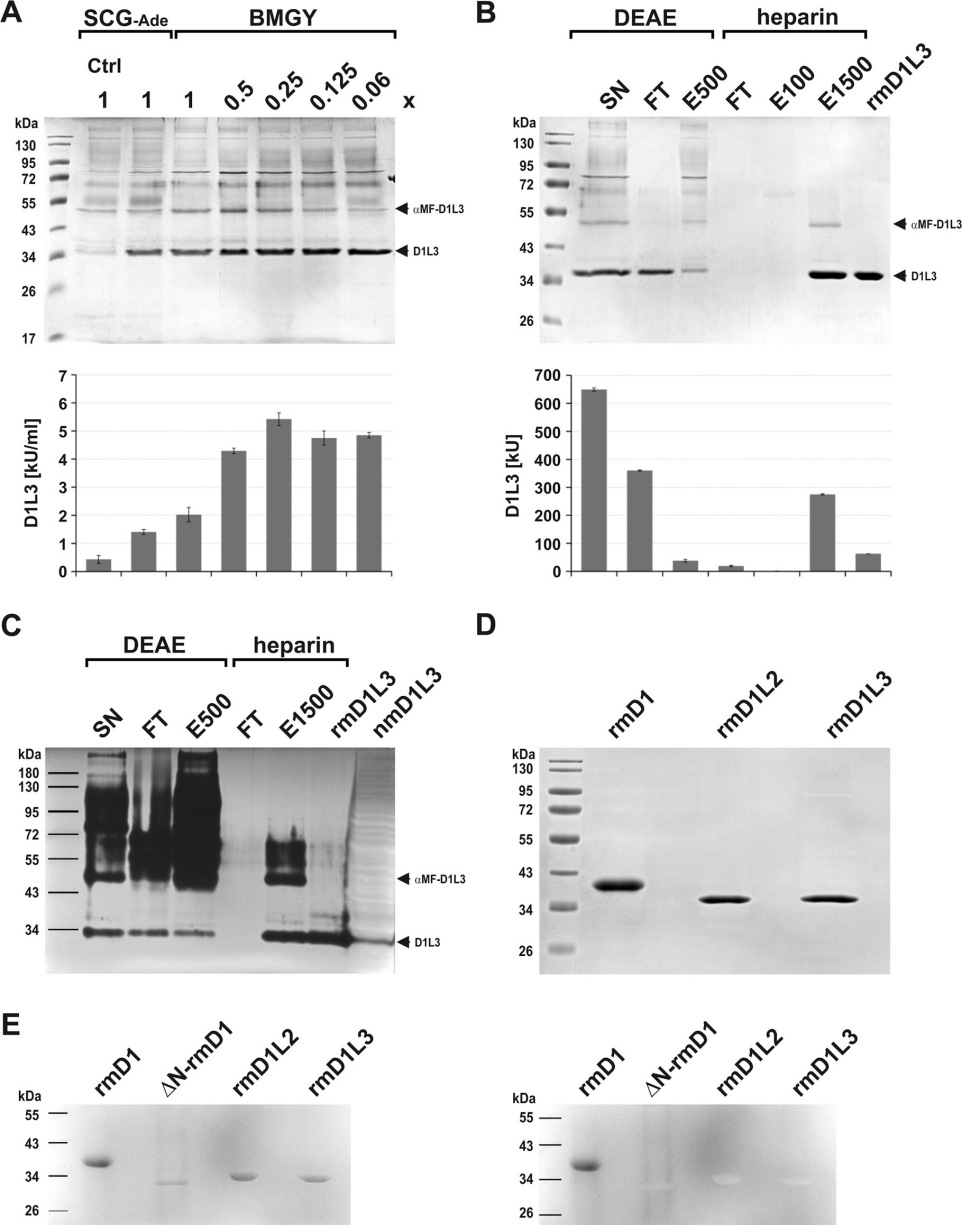
Establishment of expression and purification of rmDNase1L3 from culture supernatant. (A) Expression and processing of pre-mature αMF-DNase1L3 (αMF-D1L3, ~49 kDa) to mature rmDNase1L3 (D1L3, ~35 kDa) is less dependent on the choice and nutrient content of the growth medium (1x BMGY: 1% yeast extract, 2% (w/v) peptone) than that of rmDNase1 and rmDNase1L2 (PM expression medium was used). (B) Purification of mature rmDNase1L3 from diluted SN by DEAE-cellulose anion-exchange and Heparin-Sepharose affinity chromatography (FT: flow through, E50-1500: elution fraction at 50–1500 mM NaCl). (A, B) Top image: Coomassie-stained SDS-gel, lower image: specific nuclease activity of the samples measured by HCA. (C) Successful separation of pre-mature αMF-DNase1L3 from mature rmDNase1L3 as shown by DPZ. The final pure rmDNase1L3 protein co-migrates with native mDNase1L3 (nmD1L3) present in splenic tissue homogenate of a *Dnase1* KO mouse (40 μg protein loaded). (D) Direct comparison of the purified recombinant secretory DNase1 proteins by SDS-PAGE and Coomassie-staining (each 4 μg). (E) Detection of glycosylation by Periodic acid-Schiff staining of an SDS-PAGE gel. Only rmDNase1 (rmD1) is glycosylated whereas de-N-glycosylated rmDNase1 (ΔN-rmD1), rmDNase1L2 (rmD1L2) and rmDNase1L3 (rmD1L3) lack glycosylation. Left image: stained gel prior to washing, right image: stained gel after washing with diluted acetic acid. Marker: PageRuler™ Prestained Protein Ladder.

Finally, we attempted the expression of mature mDNase1L3, which consists of 285 aa with a calculated MM of ~33 kDa (Fig 1A and Table 2). For expression, the cDNA encoding mature mDNase1L3 was cloned in fusion to the αMF-SP in pPinkα-HC. Unintentionally the cDNA contained a nucleotide exchange, which led to a conservative K to R mutation in the second NLS (see Materials and methods). Comparable to the results for rmDNase1L2, two expression cassettes were necessary to reach elevated nuclease activity in the SN of transgenic clones (Fig 7A). Again, we optimized expression and found analogous to rmDNase1 that BMGY growth medium with 0.25x YP in combination with PM expression medium was optimal. Most of rmDNase1L3 in the SN was mature and migrated at ~35 kDa in SDS-PAGE, whereas pre-mature αMF-DNase1L3 migrated at ~49 kDa (Fig 7A).

### Purification of rmDNase1L3 from culture supernatant

Mature mDNase1L3 has a pI-value of 8.85 with a charge of +9.91 at pH 6.0 (Table 2). Although rmDNase1L3 did not efficiently bind to DEAE-cellulose at pH 6.0, we applied it to remove contaminants. Therefore, the diluted SN was loaded on an equilibrated DEAE-cellulose column. About 55% of rmDNase1L3 activity remained in the FT, whereas contaminants bound and eluted at 0.5 M NaCl (Fig 7B). The FT was further processed by Heparin-Sepharose affinity chromatography. Similar to rmDNase1L2, rmDNase1L3 eluted at 1.5 M NaCl with a yield of ~74% (Fig 7B). However, E1500 still contained residual pre-mature αMF-DNase1L3 (Fig 7B). Final purification was achieved by gel-filtration comparable to rmDNase1 and rmDNase1L2. SDS-PAGE and DPZ showed that rmDNase1L3 was separated from pre-mature αMF-DNase1L3 and contaminants (Fig 7B and 7C). It co-migrated with native mDNase1L3 present in splenic tissue homogenate from a *Dnase1* KO mouse. Like rmDNase1 and rmDNase1L2, rmDNase1L3 stored at -20°C was stable for at least one month as determined by HCA (3.90±0.56 versus 3.68±0.16 kU/nmol). About ~10% of the nuclease activity present in the SN was isolated as pure mature rmDNase1L3 with a yield of maximal 3 mg/l expression culture.

### Glycosylation of the rmDNases

Comparing the rmDNases by SDS-PAGE, we confirmed their purification to homogeneity in the mature form (Fig 7D). Since the MM of all nucleases appeared marginally higher in SDS-PAGE (rmDNase1: ~37 kDa, rmDNase1L2: ~34 kDa, and rmDNase1L3: ~35 kDa) than calculated (Fig 1A and Table 2), we stained the gel according to the Periodic acid-Schiff procedure to screen for glycosylation. In accordance with the de-N-glycosylation of rmDNase1 shown in Fig 6E, rmDNase1 is the only member of the secretory DNase1 family, which possesses glycosylation at all (Fig 7E).

### Specific activities of the rmDNases

So far, the specific activities of the mDNase1 family members were not determined in comparison. A prerequisite for this is their comparable recombinant expression and purification with sufficient yields of pure protein, since it had not been possible to isolate all DNases to homogeneity from their natural sources. The most common assay allowing quantification is the HCA [62], which we applied at different pH-values in the presence of Ca^2+^ in combination with either Mg^2+^ ions or one of the essential trace elements Co^2+^ and Mn^2+^ as co-factors.

**Fig 8:**
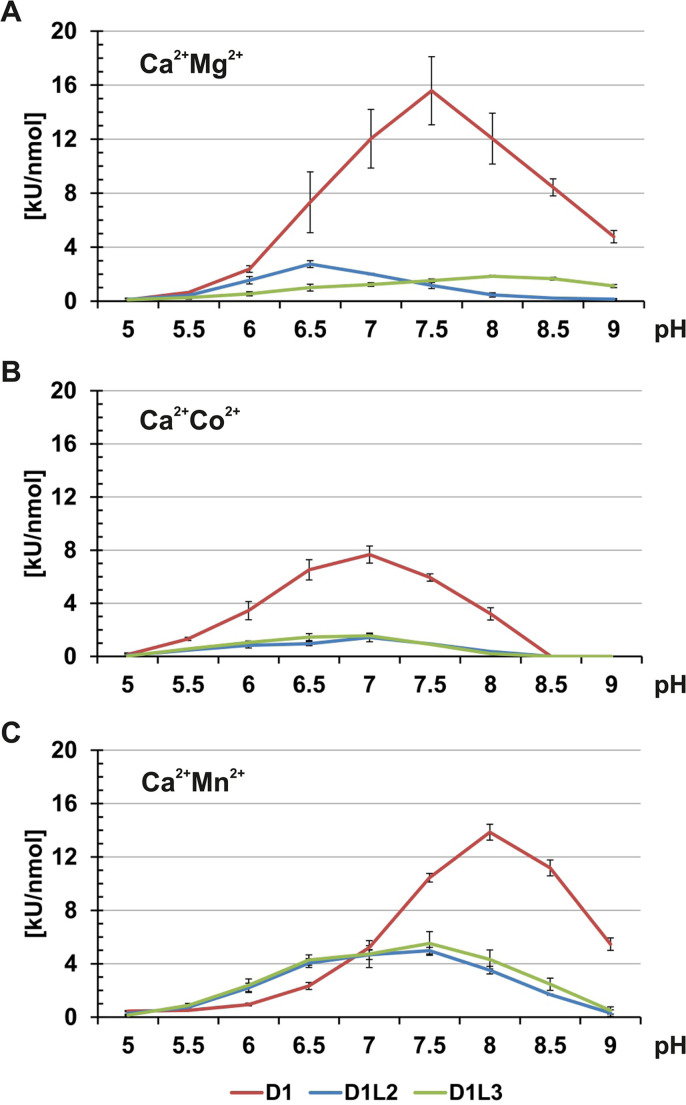
Specific activities of the rmDNases. Hyperchromicity assay at different pH-values in the presence of 0.1 mM CaCl_2_ and either 1 mM MgCl_2_ (A), CoCl_2_ (B) or MnCl_2_ (C). Equimolar amounts of the recombinant nucleases were tested (30 μM).

In the presence of Ca^2+^ and Mg^2+^ ions, the nucleases covered the broadest pH-range with respect to their optima (Fig 8A). Whereas rmDNase1L2 (2.8±0.3 kU/nmol) displayed a slightly acidic optimum at pH 6.5, rmDNase1 (15.6±2.5 kU/nmol) and rmDNase1L3 (1.8±0.1 kU/nmol) showed a slightly basic optimum at pH 7.5 and 8.0, respectively (Fig 8A and Table 2). The difference in the specific activity between rmDNase1 and the others was greatest, i.e. ten times higher, at pH 7.5 (Fig 8A). Replacing Mg^2+^ by Co^2+^ ions led to an alignment of all optima to pH 7.0 with a difference of about five times between the specific activity of rmDNase1 and the others (Fig 8B and Table 2). The specific activity of rmDNase1L3 did not change significantly, whereas it was reduced by ~50% for rmDNase1 and rmDNase1L2 at optimal pH 7.0. Replacing Co^2+^ or Mg^2+^ by Mn^2+^ ions selectively increased the specific activities of rmDNase1L2 and rmDNase1L3 over a broad pH-range (Fig 8C and Table 2). Consequently, the specific activities of all nucleases aligned at neutral pH 7.0. At pH 7.5, rmDNase1 (10.4±0.3 kU/nmol) was only twice more active than rmDNase1L2 (5.0±0.3 kU/nmol) and rmDNase1L3 (5.5±0.9 kU/nmol) (Fig 8C).

### Substrate specificities of the rmDNases

**Fig 9:**
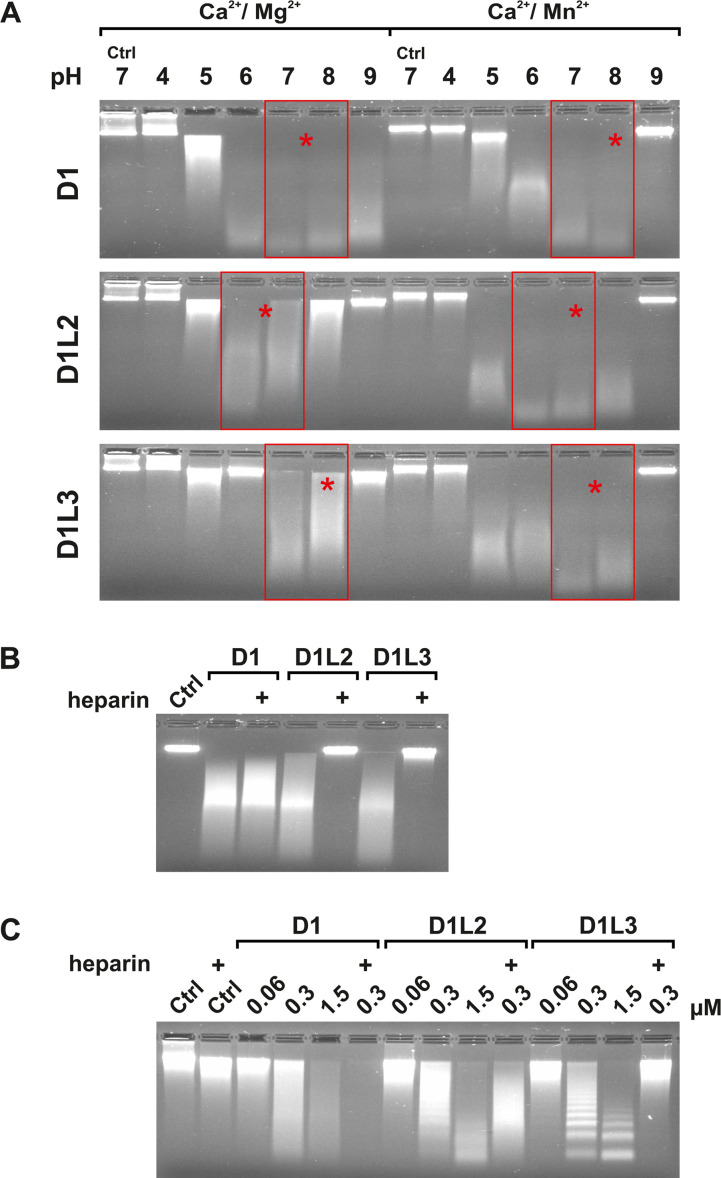
Substrate specific activities of the rmDNases. (A, B) Lambda DNA digestion at different pH-values in the presence of 2 mM CaCl_2_ and either 2 mM MgCl_2_ or MnCl_2_. Aliquots of the assay were analyzed by agarose gel electrophoresis and subsequent EtBr-staining. Equimolar amounts of the recombinant nucleases were tested (0.3 μM). Optimal pH-ranges are framed in red and the optimal pH-value estimated by HCA (Fig 8) is marked by an asterisk *. Consistent with the HCA, all three nucleases exert the same activity at pH 7.0 in the presence of Mn^2+^ ions, whereas at pH 7.5 in the presence of Mg^2+^ ions rmDNase1 (D1) exerts an activity, which is ten times higher as quantified by HCA. (B) In contrast to rmDNase1, heparin inhibits rmDNase1L2 (D1L2) and rmDNase1L3 (D1L3) in the protein-free lambda DNA assay at pH 7.0 in the presence of 2 mM CaCl_2_ and MnCl_2_. (C) In contrast to protein-free lambda DNA in (A), all three nucleases exert an almost equal ability to degrade chromatin at pH 7.5 in the presence of 2 mM CaCl_2_ and MgCl_2_ using cell nuclei of murine NIH-3T3 fibroblasts. However, the cleavage mode differs (rmDNase1: random, rmDNase1L2: bi-modular and rmDNase1L3: internucleosomal) as evaluated by agarose gel electrophoresis and subsequent EtBr-staining. Heparin accelerates the activity of rmDNase1 and inhibits that of rmDNase1L3 as previously shown [37]. In contrast to the lambda DNA assay, rmDNase1L2 is not inhibited by heparin but its cleavage mode changes from bi-modular to exclusively random.

We confirmed the quantitative results obtained by HCA with a semi-quantitative assay investigating DNA degradation by agarose gel electrophoresis. Instead of plasmid DNA [37, 71], we used phage lambda DNA, which is linear and of defined length (48.5 kbp). In contrast to plasmid DNA, it has the advantage that degradation is directly visible by the generation of DNA fragments, whereas plasmid DNA first undergoes conformational changes from supercoiled, to relaxed and linear during DNA hydrolysis, which complicates a qualitative comparison of the DNase activities. Equimolar amounts of the nucleases were tested at different pH-values in the presence of Ca^2+^ with either Mg^2+^ or Mn^2+^ ions as a cofactor (Fig 9A). At pH 7.0 and in the presence of Mn^2+^ ions, all DNases exerted the same efficiency to degrade protein-free DNA, whereas at pH-values between 7.0 and 8.0 and in the presence of Mg^2+^ ions the greatest difference existed (Fig 9A). In agreement to previous studies [37], heparin inhibited rmDNase1L3 but not rmDNase1, and for the first time we demonstrate that rmDNase1L2 was also inhibited by heparin (Fig 9B). To evaluate whether these findings also apply to protein-complexed DNA, we performed a nuclear chromatin digestion assay (Fig 9C). In contrast to protein-free DNA, equimolar amounts of the DNases comparably degraded chromatin at pH 7.5 in the presence of Ca^2+^ and Mg^2+^ ions. Remarkably, rmDNase1 cleaved randomly, whereas rmDNase1L3 cleaved preferentially at internucleosomal sites [37] (Fig 9C). Notably, the cleavage ability of rmDNase1L2 appeared to be an intermediate, i.e. is bi-modular. Chromatin cleavage by rmDNase1 was enhanced by heparin, whereas rmDNase1L3 was inhibited [37]. In contrast to protein-free DNA, heparin did not inhibit rmDNase1L2 in chromatin cleavage but changed its cleavage pattern from bi-modular to exclusively random (Fig 9C).

### Interaction of monomeric α-actin with the rmDNases

**Fig 10:**
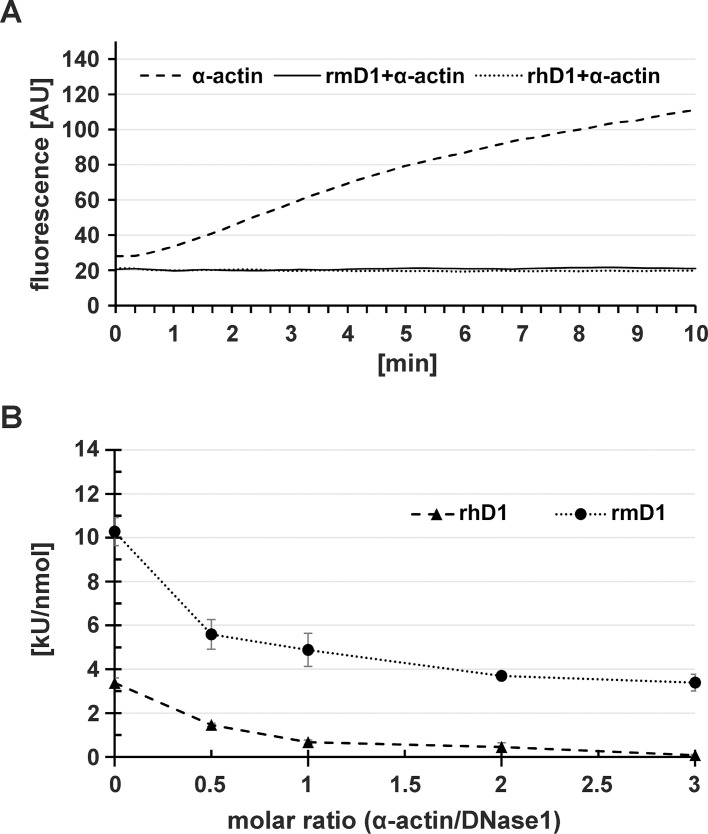
Interaction of monomeric α-actin with rhDNase1 and rmDNase1. (A) Polymerization of monomeric skeletal muscle α-actin supplemented with fluorescent pyrenyl-labeled monomeric α-actin to microfilaments in the absence and presence of rhDNase1 (rhD1, Pulmozyme™, Roche) or rmDNase1 (rmD1) at equimolar ratio (2.5 μM). (B) Effect of increasing monomeric α-actin concentrations on the DNase1 activity as determined by HCA at pH 7.0 in the presence of 0.1 mM CaCl_2_ and 1 mM MgCl_2_.

Finally, we evaluated binding of the DNases to monomeric skeletal muscle α-actin, which is well known for bovine DNase1 [51]. We observed that recombinant human (rh)DNase1 and rmDNase1 comparably inhibited α-actin polymerization at equimolar ratio (Fig 10A) illustrating their equal actin binding ability (Fig 1A). In contrast to 53% for rmDNase1, 80% of rhDNase1 activity was inhibited by equimolar amounts of monomeric α-actin (Fig 10B). Increasing the α-actin:DNase1 ratio led to complete inhibition of rhDNase1, whereas inhibition of rmDNase1 was again less effective (Fig 10B). These data hint to an altered conformation of the actin binding-site or its position relative to the catalytic centre in both DNases. Consistent with the non-conserved binding region for monomeric α-actin (Fig 1A), we found no significant effect of rmDNase1L2 and rmDNase1L3 on α-actin polymerization and *vice versa* of α-actin on the activity of both nucleases (S4 Fig). In comparison to Mn^2+^, inhibition of rhDNase1 and rmDNase1 by monomeric α-actin occurs with higher efficiency in the presence of Mg^2+^ ions (S4 Fig).

## Discussion

The present study describes recombinant expression of the secretory mDNase1 family members using *P*. *pastoris*. The major goal was to produce sufficient amounts of purified nucleases using a eukaryotic system resembling most closely the natural expression conditions and omitting interventions such as purification tags, protease recognition sites for post-expressional processing or optimization of the KEX2 cleavage site. Nevertheless, we are aware of the heterologous protein expression system. Unfortunately, standard protocols led to limited expression levels and therefore had to be modified as described. Increasing the gene dosage was effective, which is consistent with the recommendation to select multi-copy transgenic yeast or to increase the number of expression cassettes in the transformed vector [70, 72]. The first procedure, however, necessitates long-lasting high throughput screening, while the latter can be realized by a targeted and faster way. Besides gene dosage, optimizing protein maturation was found to be important since ~50% of the recombinant nucleases was pre-mature, i.e. was still bound to an extensively tri-N-glycosylated pro-peptide. Limited protein maturation and hyper-mannosylation are known for *P*. *pastoris* when using the αMF-SP. They result from an overload of the secretory machinery such as an inefficient pro-peptide cleavage by KEX2 [73, 74]. Known reasons are for example the size of the target protein or the removal of the di-basic spacer-peptide from the αMF-SP [75]. In addition, extensive glycosylation might sterically hinder binding of KEX2 to its substrate [76]. Appropriate methods to overcome the inefficient maturation were reported to be the co-overexpression of KEX2 [77, 78], the post-expressional *in vitro* maturation by recombinant KEX2 [79] or the expression of the target protein with its native SP [70]. However, none of these approaches led to an improvement in our experiments.

Instead and consistent with previous findings, adjusting the equilibrium of recombinant protein translation and processing by starvation was successful [70, 80]. Thus, media with reduced nutrients and washing the cells with PBS prior to expression, improved protein expression and maturation. However, the latter finding could also be explained by a more efficient removal of glycerol, which is a repressor of the *AOX1* promoter [70, 81]. Appropriate media compositions appear to be very important and should be carefully evaluated for every protein of interest. Nutrient adaption reduced glycosylation and increased maturation, which correlates with the theory that extensive glycosylation sterically interferes with KEX2 binding.

In addition to nutrient adaption, we found a new procedure to process pre-mature rmDNase1 by N-terminal trimming employing dialysis of the SN. Maturation was facilitated by protease(s) in the SN and for their release *P*. *pastoris* had to be conditioned during biomass production. Trimming was not accompanied by loss of function and led to stable mature rmDNase1. We did not identify these protease(s), yet stress-induced up-regulation and release of yapsin 1 for example has been described [82]. However, yapsin 1 cannot be inhibited by AEBSF, which is in contrast to the retarded maturation of pre-mature DNase in the presence of AEBSF in our experiments [83]. Nevertheless, it cleaves the same di-basic motif as KEX2, pointing to the co-existence of comparable proteases in *P*. *pastoris* [84]. Furthermore, it has been shown that methanol, but not glycerol induces secretion of subtilisin 2, a PMSF inhibitable serine protease [85]. These examples demonstrate that although strain 4 of *PichiaPink*^*TM*^ *pastoris* lacks proteinase A, proteinase B and carboxypeptidase Y, other proteases might be released probably as a result of cell damage or due to misdirection under stress conditions [86].

After expression, purification procedures according to the biochemical properties of the nucleases were performed demonstrating that rmDNase1L2, as rmDNase1L3, binds to heparin [37]. After purification to homogeneity, all nucleases were shown to be functional and stable in storage buffer. The purified DNases were comparable to their mammalian originals even though the rmDNase1L3 contains an unintentional conservative K to R mutation in the second NLS (see Materials and methods). For example, rmDNase1, in contrast to the non-glycosylated rmDNase1L2 and rmDNase1L3, was found to be di-N-glycosylated as described in prior research [37]. This might explain its reduced processing by KEX2 (sterical hindrance) and its higher expression level probably caused by a greater proteolytic resistance [87]. We found no indication for the presence of protein or further contaminants influencing the biochemical characterization of the final DNase preparations. The prerequisite of contaminants would be that they have identical chromatographic behaviour and MM compared to the DNases. We assume that contaminations are rather unlikely or of only low amount, since it was possible to separate pre-mature from mature DNase differing by only 7 kDa by gel-filtration. It will be interesting to compare our DNase preparations with those using tags for affinity purification with respect to enzymatic properties and contaminants.

The subsequent biochemical analysis allowed a direct quantitative comparison of the specific activities of the mDNase1 members towards different substrates and under varying conditions for the first time. Previously, such studies were not possible because only defined volumes of expression SN or enriched preparations of His- or Myc-tagged nucleases were available for analysis [7, 37, 71]. Using HCA and the lambda DNA assay we showed that in the presence of the most abundant divalent cations Ca^2+^ and Mg^2+^, the three DNases cover the broadest pH-range with respect to their optima. Under these conditions, mDNase1 is the major nuclease for the degradation of protein-free DNA with an optimum at neutral to slightly basic pH-value. This large activity difference in comparison to the other DNases diminishes in the presence of either Co^2+^ or Mn^2+^ in combination with Ca^2+^ ions. While the presence of Co^2+^ ions equalizes the pH-optima of all DNases and reduces their activity, Mn^2+^ ions only equalize the pH-optima of rmDNase1L2 and rmDNase1L3 and increase their activity. However, they do not affect rmDNase1. Quite notably, at pH 7.0 in the presence of Mn^2+^ ions, all DNases exert an essentially identical activity. So far, the difference between the earth alkali (Ca^2+^, Mg^2+^) and the transition metal ions (Co^2+^, Mn^2+^), when used alone, is explained by the promotion of single versus double-stranded DNA hydrolysis [88]. However, this does not explain why Mn^2+^, but not Co^2+^, in comparison to Mg^2+^ ions enhances the activity of rmDNase1L2 and rmDNase1L3. It also does not explain why rmDNase1 is more active in the presence of Mg^2+^ than Mn^2+^ or Co^2+^ ions. Instead, it appears that the catalytic centre of mDNase1 is built differently from that of mDNase1L2 and mDNase1L3 and that all metal ions investigated have different abilities to coordinate the scissile phosphodiester bond of the DNA within the catalytic centre [14]. So far, no data are available about the 3D-structure of mDNase1L2 or mDNase1L3. In addition, no crystallographic data exist for mammalian DNases in the presence of cations other than Ca^2+^ and Mg^2+^. It will be interesting to use the purified DNases for such investigations, as the necessary amounts can now be provided. Furthermore, it remains to be elucidated whether natural situations exist, under which the different cation-dependent DNA degrading activities might be of importance. Compared to the study of the human DNase1 family, which was expressed with His-tags, we determined similar pH-optima for the tag-free mDNases in the presence of Ca^2+^ and Mg^2+^ ions [7]. However, our study shows for the first time that the pH-optima vary with respect to the ion composition used. In addition to the estimation of pH-optima, specific activities were determined in our study for the first time. Previously determined pH-optima for rmDNase1 and rmDNase1L3 present within the SN of transfected culture cells differ from those in the present study [37]. This may be explained by the high bicarbonate and buffer concentration of the culture media, i.e. the SN, employed in small-scale plasmid DNA assays [37]. This finding points to the importance to use purified DNases for their biochemical characterization.

In contrast to the digestion of protein-free DNA, all nucleases display a comparable efficiency to degrade chromatin in the presence of Ca^2+^ and Mg^2+^ ions. This is consistent with the finding that mDNase1 and mDNase1L3 in the blood stream equally participate in the dissolution of NETs [41]. The chromatinolysis pattern differs from random (rmDNase1) to internucleosomal (rmDNase1L3) as previously described, although its biological consequence is unknown [71]. Thereby the pattern might depend on the charge and the tertiary structure of the nucleases. Non-glycosylated “small” basic proteins such as mDNase1L3 might more easily replace histone H1 from chromatin than the N-glycosylated acidic mDNase1. This scenario would preferentially render internucleosomal regions accessible for chromatinolysis by mDNase1L3, whereas mDNase1 might attack all exposed regions of the DNA backbone with a similar efficiency. Interestingly, rmDNase1L2 shows a bi-modular cleavage mode, which could be explained by the hypothesis described above: It possesses an acidic pI-value, but is not glycosylated and therefore, unlike mDNase1, might also gain access to internucleosomal regions. Displacement of histones from chromatin appears to be a necessary requirement for its optimal degradation. Heparin and proteases such as plasmin have this ability and thereby promote chromatinolysis by rmDNase1 as shown previously and in this study [37, 89]. Thus, the large difference in the specific activity between rmDNase1 and the other DNases towards protein-free DNA at pH 7.5 in the presence of Ca^2+^ and Mg^2+^ ions is maintained in the presence of heparin. Under these circumstances, the chromatin cleavage pattern of rmDNase1L2 switches from bi-modular to exclusively random, whereas the basic rmDNase1L3 is completely inhibited. The reason why rmDNase1L2 is not inhibited by heparin when cleaving chromatin instead of protein-free DNA might be explained by a higher affinity of the polyanionic heparin to basic histones than to the acidic rmDNase12. Displacement of histones from chromatin ultimately results in the exclusively random DNA cleavage pattern by rmDNase1L2. From these results, it appears that heparin treatment should be carefully considered during therapeutic regimes. As long as DNase1 and DNase1L3 occur together in the presence of heparin, the enhanced chromatinolysis by DNase1 might compensate the inhibition of DNase1L3. However, in situations where macrophage derived DNase1L3 is probably dominating (inflammation), heparin treatment might be contra-indicated. In this context it remains to be elucidated, which natural role heparin has on DNase1L3. A common feature of rmDNase1L2 and rmDNase1L3 is their inability to bind to monomeric actin, which is again in agreement with their human counterparts [7]. This might point to their postulated role in intracellular chromatin cleavage of cells undergoing differentiation or apoptosis [21, 22, 48]. In contrast, DNase1 appears to function primarily extracellularly or in necrotic cells and is mostly inhibited by monomeric actin when released intracellularly [44, 45, 47, 52]. Alternatively or in parallel, residual amounts of liberated intracellular active DNase1 may induce the upregulation of apoptotic endonucleases as proposed recently [53]. Probably, heparin fulfills a role similar to monomeric actin by inhibiting misdirected DNase1L2 and DNase1L3.

Taken together, our results indicate that mDNase1L2 is an intermediate isoform of mDNase1 and mDNase1L3 with conserved features of both of them. Since mDNase1 and mDNase1L2 share the highest sequence identity and similarity and have both an almost equal identity and similarity to mDNase1L3, it appears that mDNase1L3 has diverged earlier from a common ancestor (Table 2). Whereas biochemical features present in all members of the DNase1 family can be definitely attributed to a common phylogenetic ancestor (see introduction), those shared by either mDNase1 or mDNase1L2 with mDNase1L3 might have been evolutionarily conserved. In contrast, individual features appear to be acquired during further differentiation. Thus, heparin binding, the ability for internucleosomal chromatin degradation and a preference for Ca^2+^ and Mn^2+^ ions appear to be characteristics of the common ancestor. In contrast, N-glycosylation (mDNase1), a preference for Mg^2+^ ions (mDNase1), monomeric actin binding (mDNase1), nuclear localization signals (mDNase1L3), or an exclusively random chromatin degradation (mDNase1) may have developed later individually. Thus, mDNase1L2 can be functionally regarded as an evolutionary intermediate isoform of mDNase1 and mDNase1L3.

Finally, our results demonstrate that the rmDNases are functional and suitable for further molecular and biological studies. In addition, it is worth considering their implementation as an additional therapeutic option for the treatment of micro-vessel congestion caused by aggregated NETs and damaged endothelial cells as found in patients suffering for instance from sepsis, autoimmune and cardiovascular diseases, and currently from a SARS-CoV2 infection [89, 90].

## Conclusions

This manuscript demonstrates successful recombinant expression of the secretory (soluble) murine DNase1 family members in *P*. *pastoris* resulting in the purification of biologically functional DNases, which were shown to be comparable to their native counterparts. Limitations of expression such as a reduced αMF-SP pro-peptide processing with accumulation of pre-mature DNase was overcome by adaption of the culture media compositions and by pro-peptide cleavage by stress-induced proteases. Our data describe for the first time an experimental protocol for the production and purification of these DNases in amounts suitable for investigation of their specific activities. Under the most prevalent extracellular conditions (high Ca^2+^ and Mg^2+^ ion concentrations, pH 7.4), rmDNase1, rmDNase1L2 and rmDNase1L3 display an equal ability to catalyse chromatinolysis, whereas rmDNase1 is significantly more active in protein-free DNA degradation. This difference in activity changes when Mn^2+^ replace Mg^2+^ ions, since Mn^2+^ ions align the specific activities of the DNases. From the comparative characterization, it can be concluded that mDNase1L2, which was so far not extensively investigated, is an evolutionary intermediate isoform of the three DNases with respect to its biochemical properties.

## Supporting information

S1 Fig Protease inhibitors do not prevent degradation of rmDNase1L2. Analysis of SN prepared from expression cultures for rmDNase1L2 (SCM-Ade) by SDS-PAGE and Coomassie staining. (A) Expression in the absence or presence of the serine protease inhibitor AEBSF (1 mM) or bovine serum albumin (0.01% (w/v) BSA) to protect against a postulated protease degradation. (B) As described in Materials and methods, 0.5 ml SN was concentrated with a 3K filter unit by centrifugation. Addition of a protease inhibitor cocktail (PIC) during concentration and/or dialysis by H_2_O did not prevent degradation of rmDNase1L2, which can be also seen in Fig 2B. Marker: PageRuler™ Prestained Protein Ladder in (A) and Cozy Prestained Protein Ladder in (B).
(TIF)

S2 Fig Expression of rmDNase1L2 with its natural signal peptide. (A) Detection of single transgene integrations in a transformed clone for expression of mDNase1L2 with the αMF-SP (αMF-D1L2) in comparison to three clones for mDNase1L2 with its natural signal peptide (SP-D1L2) by expression cassette specific PCR (αMF-D1L2: 1197 bp, SP-D1L2: 1011 bp, pos. Ctrl: vector, neg. Ctrl: water, marker: GeneRuler 1 kb DNA Ladder). (B) Analysis of SN prepared from induced (MeOH) or not induced SCM-Ade expression cultures of the different clones by SDS-PAGE and Coomassie staining. Marker: PageRuler™ Prestained Protein Ladder.
(TIF)

S3 Fig Protease release for αMF-DNase1 maturation depends on the grade of ventilation of the growth culture. Processing of pre-mature αMF-DNase1 (αMF–D1, ~51 kDa) in 2x dialyzed expression SN of *P*. *pastoris* grown in BMGY at 30°C and shaking at either 175 or 300 rpm in baffled flasks. Processing to mature rmDNase1 (D1, ~37 kDa) depends on protease(s) released into the PM expression medium in dependence of the ventilation of the growth culture. Optimal ventilation occurs at 300 rpm. The maturation can be retarded by the protease inhibitor AEBSF (1 mM) and occurs optimally in the presence of Ca^2+^ and Mg^2+^ ions at 37°C. Marker: PageRuler™ Prestained Protein Ladder.
(TIF)

S4 Fig Interaction of monomeric α-actin with the rmDNase1 family members. (A) Polymerization of monomeric skeletal muscle α-actin supplemented with fluorescent pyrenyl-labeled monomeric α-actin to microfilaments in the absence and presence of rhDNase1 (rhD1, Pulmozyme™, Roche), rmDNase1 (rmD1), rmDNase1L2 (rmD1L2), or rmDNase1L3 (rmD1L3) at equimolar ratio (2.5 μM). In contrast to rmDNase1 or rhDNase1, rmDNase1L2 and rmDNase1L3 did not influence α-actin polymerization, which indicates a lack of molecular interaction. (B) Upper bar chart: DNase activity in the absence (Ctrl) or presence of monomeric α-actin at optimal pH-value for all nucleases as determined by HCA with Tris-buffer, pH 7.0, in the presence of 0.1 mM CaCl_2_ and 1 mM MnCl_2_. Lower bar chart: HCA at optimal pH-value for the different nucleases in the presence of 0.1 mM CaCl_2_ and 1 mM MgCl_2_ using Tris-buffer, pH 7.5 (rhDNase1 and rmDNase1), Tris-buffer, pH 8.0 (rmDNase1L3) or MES-buffer, pH 6.5 (rmDNase1L2). The inhibition of rhDNase1 by binding to monomeric α-actin is stronger than of rmDNase1 and is enhanced in the presence of Mg^2+^ in comparison to Mn^2+^ ions for both nucleases. In contrast to rmDNase1 or rhDNase1, no molecular interaction of rmDNase1L2 and rmDNase1L3 with monomeric α-actin could be detected.
(TIF)

S1 Dataset (PDF)

S1 Raw images (PDF)
