# Supplementary figures and images for: Comparison of the secretory murine DNase1 family members expressed in Pichia pastoris
Source: PLoS One. 2021 Jul 30;16(7):e0253476. doi: 10.1371/journal.pone.0253476 (PMC8323900; doi:10.1371/journal.pone.0253476)

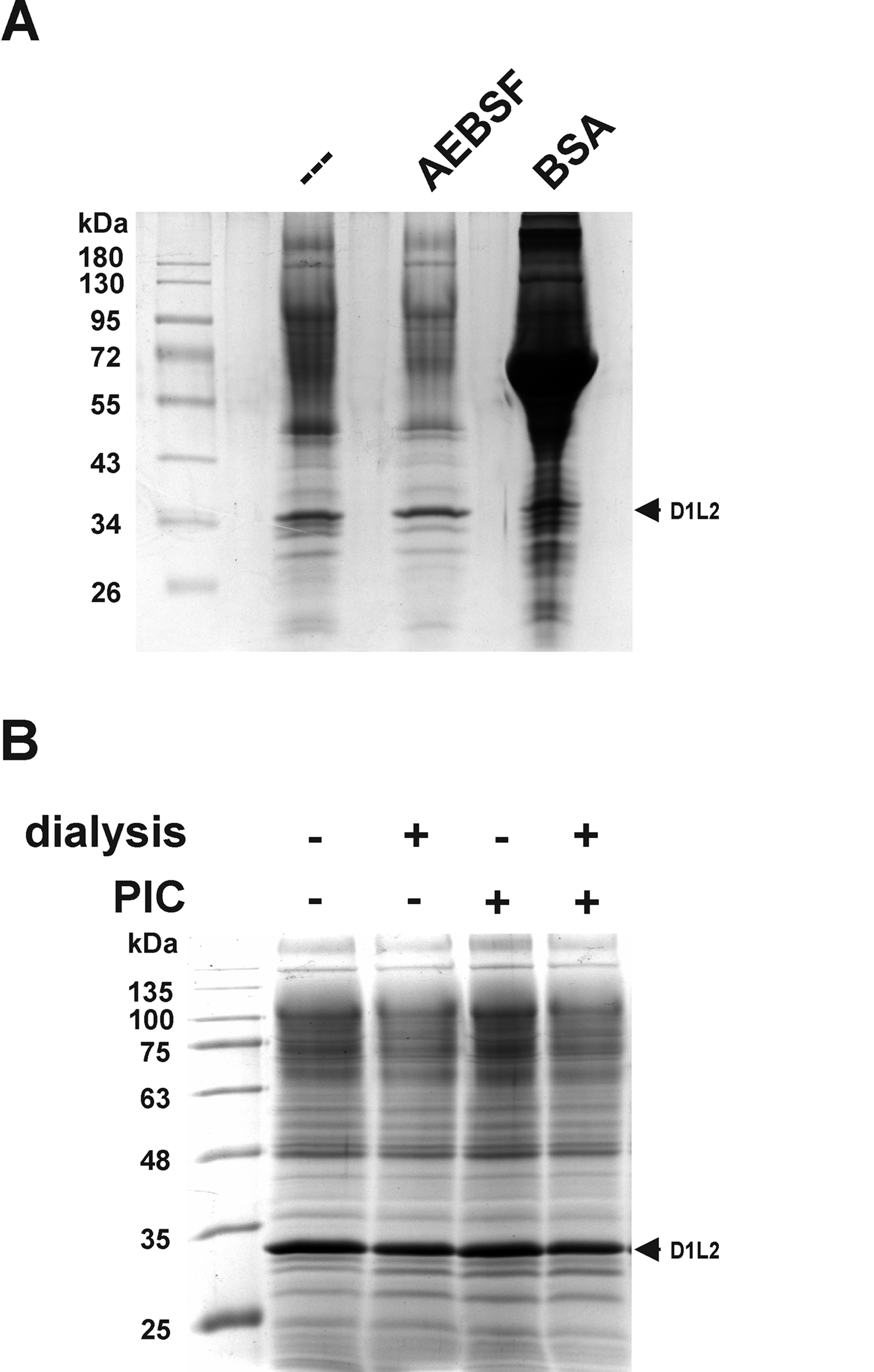

Supplement: S1 Fig — Analysis of SN prepared from expression cultures for rmDNase1L2 (SCM-Ade) by SDS-PAGE and Coomassie staining. (A) Expression in the absence or presence of the serine protease inhibitor AEBSF (1 mM) or bovine serum albumin (0.01% (w/v) BSA) to protect against a postulated protease degradation. (B) As described in Materials and methods, 0.5 ml SN was concentrated with a 3K filter unit by centrifugation. Addition of a protease inhibitor cocktail (PIC) during concentration and/or dialysis by H2O did not prevent degradation of rmDNase1L2, which can be also seen in Fig 2B. Marker: PageRuler™ Prestained Protein Ladder in (A) and Cozy Prestained Protein Ladder in (B). (TIF) [file pone.0253476.s001.tif]

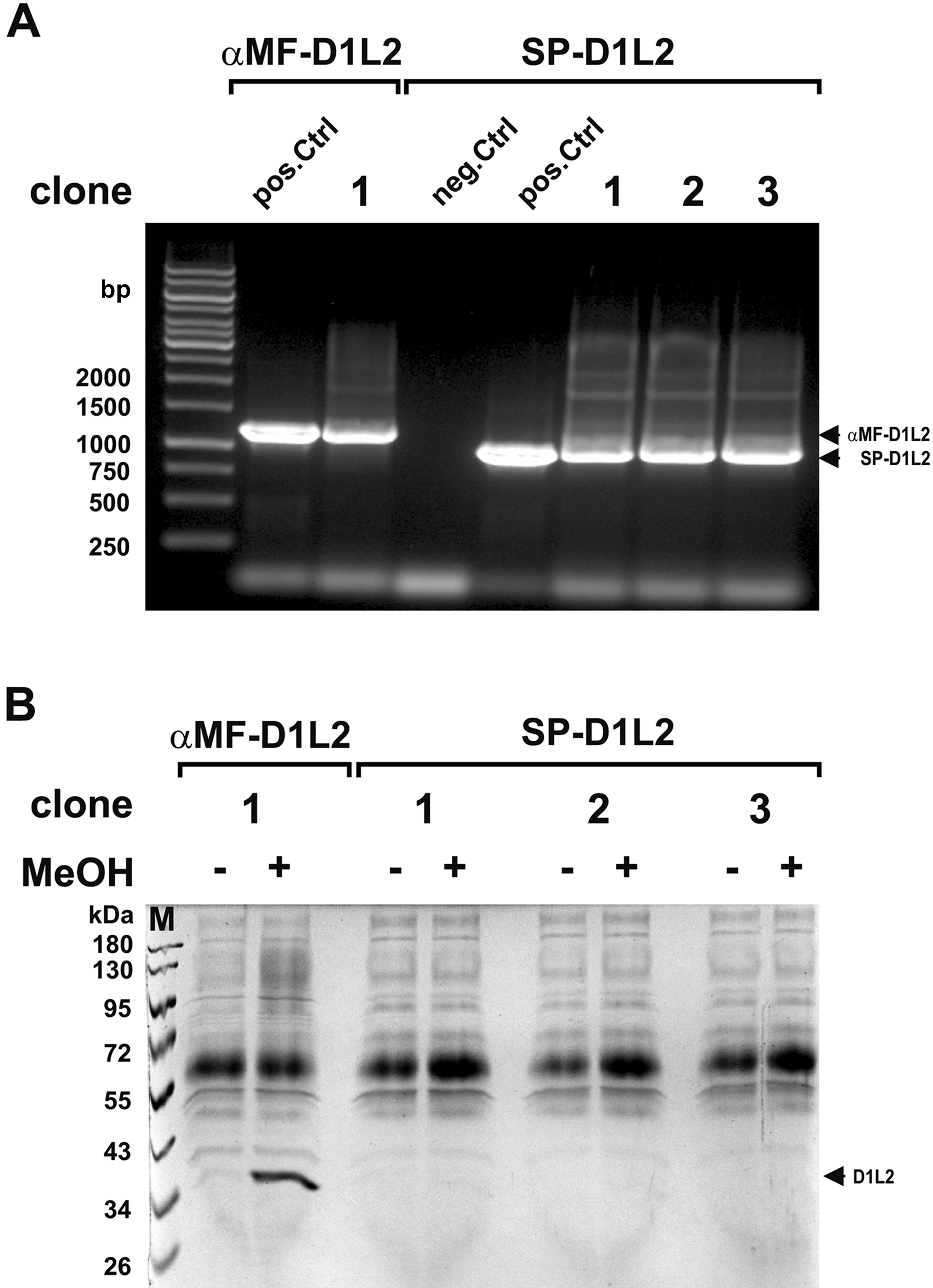

Supplement: S2 Fig — (A) Detection of single transgene integrations in a transformed clone for expression of mDNase1L2 with the αMF-SP (αMF-D1L2) in comparison to three clones for mDNase1L2 with its natural signal peptide (SP-D1L2) by expression cassette specific PCR (αMF-D1L2: 1197 bp, SP-D1L2: 1011 bp, pos. Ctrl: vector, neg. Ctrl: water, marker: GeneRuler 1 kb DNA Ladder). (B) Analysis of SN prepared from induced (MeOH) or not induced SCM-Ade expression cultures of the different clones by SDS-PAGE and Coomassie staining. Marker: PageRuler™ Prestained Protein Ladder. (TIF) [file pone.0253476.s002.tif]

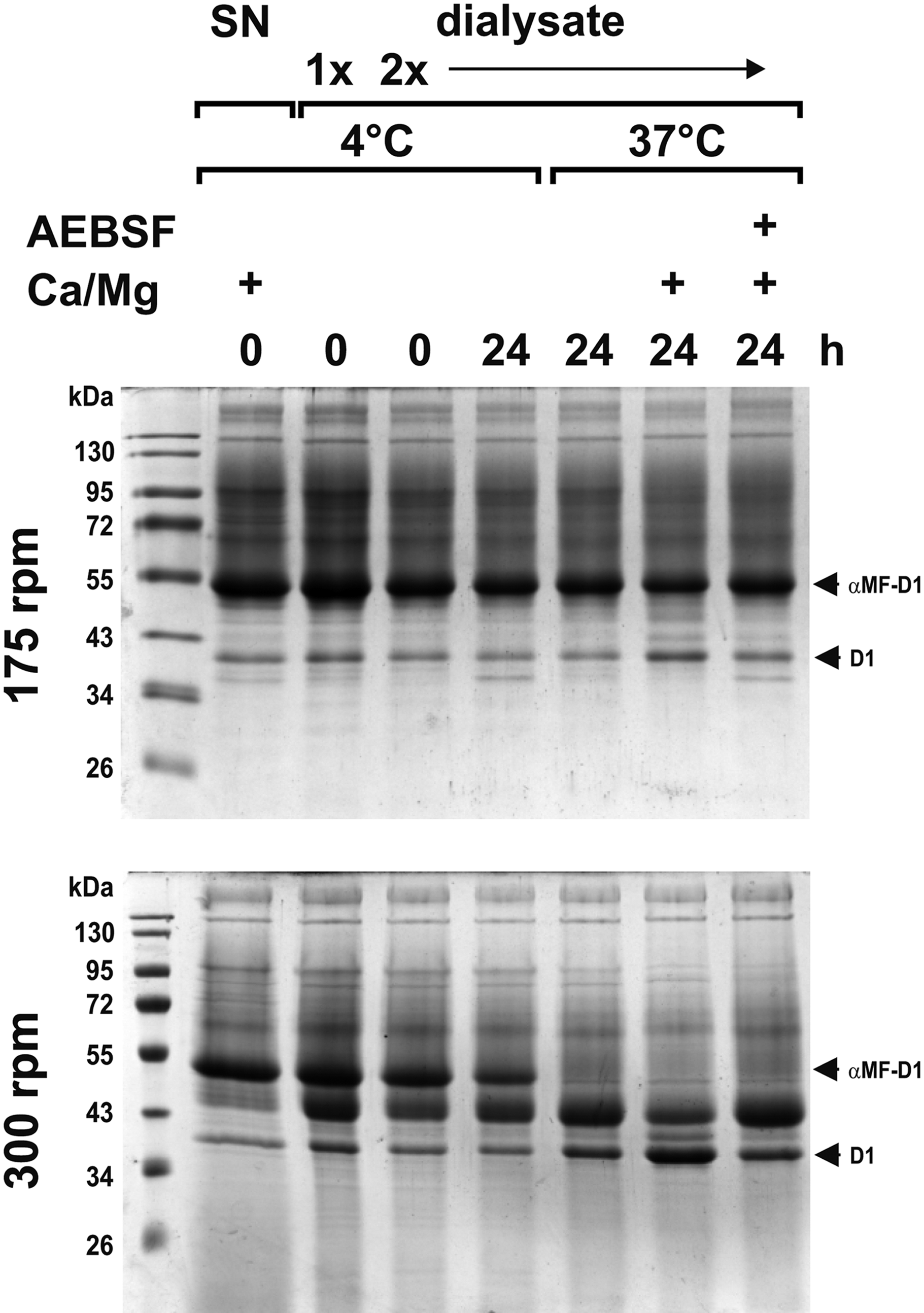

Supplement: S3 Fig — Processing of pre-mature αMF-DNase1 (αMF–D1, ~51 kDa) in 2x dialyzed expression SN of P. pastoris grown in BMGY at 30°C and shaking at either 175 or 300 rpm in baffled flasks. Processing to mature rmDNase1 (D1, ~37 kDa) depends on protease(s) released into the PM expression medium in dependence of the ventilation of the growth culture. Optimal ventilation occurs at 300 rpm. The maturation can be retarded by the protease inhibitor AEBSF (1 mM) and occurs optimally in the presence of Ca2+ and Mg2+ ions at 37°C. Marker: PageRuler™ Prestained Protein Ladder. (TIF) [file pone.0253476.s003.tif]

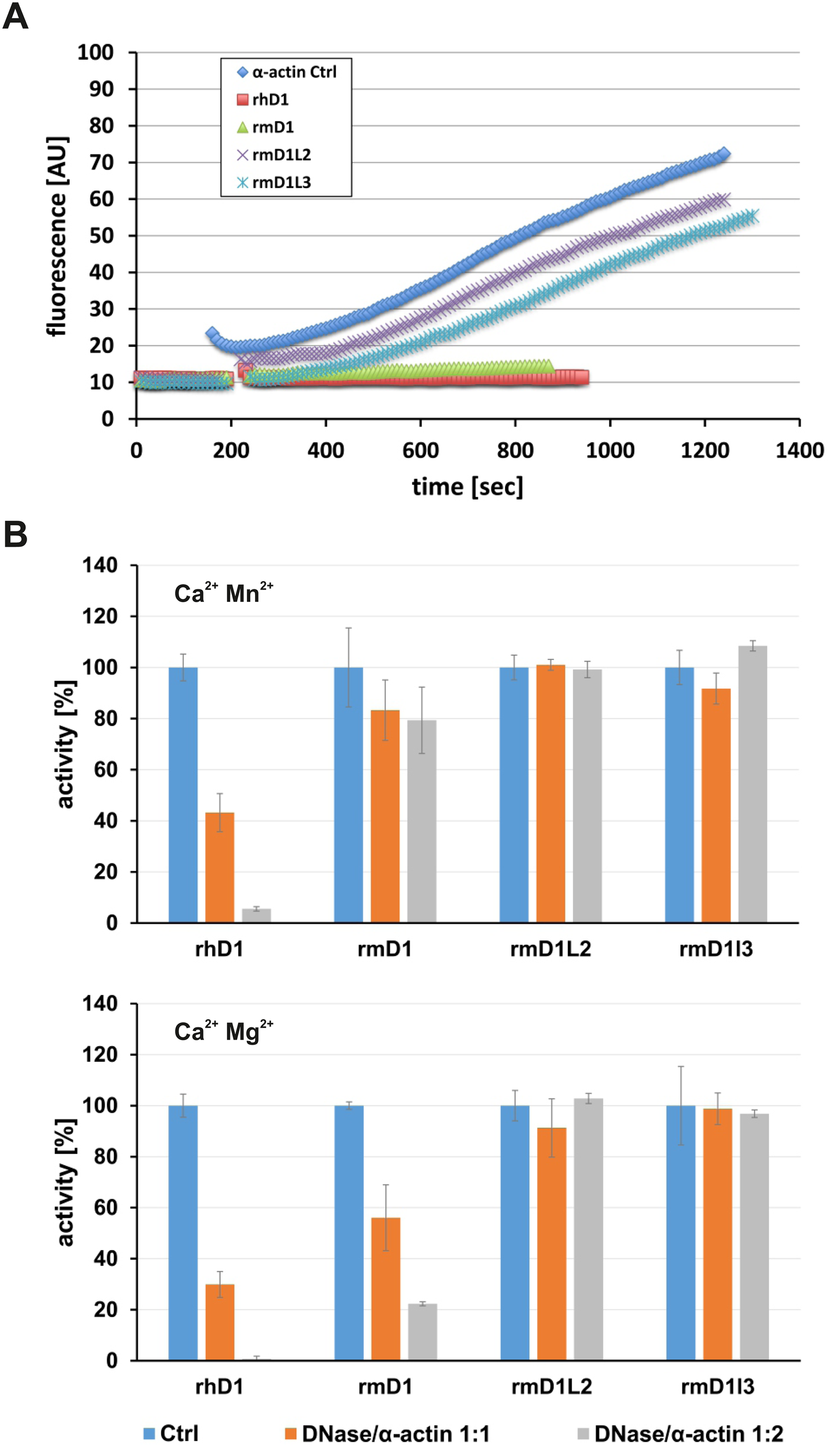

Supplement: S4 Fig — (A) Polymerization of monomeric skeletal muscle α-actin supplemented with fluorescent pyrenyl-labeled monomeric α-actin to microfilaments in the absence and presence of rhDNase1 (rhD1, Pulmozyme™, Roche), rmDNase1 (rmD1), rmDNase1L2 (rmD1L2), or rmDNase1L3 (rmD1L3) at equimolar ratio (2.5 μM). In contrast to rmDNase1 or rhDNase1, rmDNase1L2 and rmDNase1L3 did not influence α-actin polymerization, which indicates a lack of molecular interaction. (B) Upper bar chart: DNase activity in the absence (Ctrl) or presence of monomeric α-actin at optimal pH-value for all nucleases as determined by HCA with Tris-buffer, pH 7.0, in the presence of 0.1 mM CaCl2 and 1 mM MnCl2. Lower bar chart: HCA at optimal pH-value for the different nucleases in the presence of 0.1 mM CaCl2 and 1 mM MgCl2 using Tris-buffer, pH 7.5 (rhDNase1 and rmDNase1), Tris-buffer, pH 8.0 (rmDNase1L3) or MES-buffer, pH 6.5 (rmDNase1L2). The inhibition of rhDNase1 by binding to monomeric α-actin is stronger than of rmDNase1 and is enhanced in the presence of Mg2+ in comparison to Mn2+ ions for both nucleases. In contrast to rmDNase1 or rhDNase1, no molecular interaction of rmDNase1L2 and rmDNase1L3 with monomeric α-actin could be detected. (TIF) [file pone.0253476.s004.tif]
