# Supplementary material for: Comparison of the secretory murine DNase1 family members expressed in Pichia pastoris
Source: PLoS One. 2021 Jul 30;16(7):e0253476. doi: 10.1371/journal.pone.0253476 (PMC8323900; doi:10.1371/journal.pone.0253476)
Supplement: S1 Dataset — (PDF) [file pone.0253476.s005.pdf]

S1_minimal data set

**Figure 5A: DEAE chromatography of rmDNase1l2**				
HCA, Ca/Mn, pH 7.0				
Sample	Replicate 1 [kU]	Replicate 2 [kU]	Mean [kU]	Std. ±
SN	459.8	510.0	484.9	25.1
FT	17.8	4.5	11.1	6.6
E50	0.0	1.9	0.9	0.9
E100	7.7	7.2	7.5	0.3
E150	194.6	197.0	195.8	1.2
E200	287.9	274.5	281.2	6.7
E250	46.9	47.4	47.1	0.3
E300	10.0	5.3	7.6	2.4
E400	7.6	5.9	6.7	0.9
E500	1.8	0.2	1.0	0.8

Figure 5B: DEAE and Heparin chromatography of rmDNase1l2				
HCA, Ca/Mn, pH 7.0				
Sample	Replicate 1 [kU]	Replicate 2 [kU]	Mean [kU]	Std. ±
E200	346.4		346.4	10%
FT	8.9		8.9	0
E100	4.9		4.9	0
E1500	218.1	207.7	212.9	5.2
rmD1L2	123.1	137.4	130.2	7.2

**Figure 6A: rmDNase1 expression in different growth media**						
HCA, Ca/Mn, pH 7.0						
	Replicates [kU/ml]					
Sample	1	2	3	4	Mean [kU/ml]	Std. ±
1x SCG-Ade Ctrl	0.7	0.6			0.7	0.05
1x SCG-Ade	5.2	5.1	6.0	5.2	5.4	0.37
1x BMGY	6.6	6.6	6.5	6.2	6.5	0.16
0.5x BMGY	8.2	7.3	9.4	9.0	8.5	0.83
0.25x BMGY	9.1	8.9	6.3	6.9	7.8	1.21
0.125x BMGY	6.6	6.7	6.7	6.0	6.5	0.28
0.06x BMGY	6.5	6.5			6.5	0.01
0.03x BMGY	5.7	5.5			5.6	0.11
0.015x BMGY	5.4	4.6			5.0	0.36

**Figure 6B: Maturation of pre-mature αMF-DNase1 in dialyzed supernatants**				
HCA, Ca/Mn, pH 7.0				
Sample	Replicate 1 [kU/ml]	Replicate 2 [kU/ml]	Mean [kU/ml]	Std. ±
SN / 0h / 4°C	3.3	3.2	3.2	0.06
Dia / 0h / 4°C	4.8	5.0	4.9	0.11
Dia / 24h 4°C	4.5	4.2	4.3	0.15
Dia / 48h / 4°C	3.9	3.8	3.9	0.08
Dia / 24h / 37°C	4.8	4.1	4.4	0.33
Dia / 48h / 37°C	4.0	4.1	4.1	0.06
Dia / 24h / 37°C + AEBSF	4.1	4.3	4.2	0.09
Dia / 48h / 37°C + AEBSF	3.9	3.8	3.8	0.06

**Figure 6C: DEAE chromatography of rmDNase1**				
HCA, Ca/Mn, pH 7.0				
Sample	Replicate 1 [kU]	Replicate 2 [kU]	Mean [kU]	Std. ±
DN	236.0	226.5	231.3	4.75
Dialysate	223.0	240.0	231.5	8.50
FT	7.0	0	3.5	3.50
E50	0.0	0	0	0
E100	20.0	23.9	21.9	1.91
E150	168.0	174.0	171.0	3.00
E200	18.5	7.9	13.2	5.33
E250	3.8	4.7	4.2	0.41
E300	2.5	2.1	2.3	0.19
E400	1.2	1.1	1.2	0.04
E500	0	0	0	0

**Figure 7A: rmDNase1l3 expression in different growth media**				
HCA, Ca/Mn, pH 7.0				
Sample	Replicate 1 [kU/ml]	Replicate 2 [kU/ml]	Mean [kU/ml]	Std. ±
1x SCG-Ade Ctrl	0.29	0.58	0.4	0.14
1x SCG-Ade	1.50	1.32	1.4	0.09
1x BMGY	2.28	1.77	2.0	0.26
0.5x BMGY	4.39	4.20	4.3	0.10
0.25x BMGY	5.20	5.65	5.4	0.22
0.125x BMGY	5.00	4.50	4.8	0.25
0.06x BMGY	4.95	4.75	4.9	0.10

Figure 7B: DEAE and Heparin chromatography of rmDNase1l3				
HCA, Ca/Mn, pH 7.0				
Sample	Replicate 1 [kU]	Replicate 2 [kU]	Mean [kU]	Std. ±
SN	644.0	655.0	649.5	5.50
FT DEAE	358.0	362.0	360.0	2.00
E500 DEAE	33.3	43.0	38.1	4.84
FT heparin	22.0	16.0	19.0	3.00
E100 heparin	1.3	1.7	1.5	0.18
E1500 heparin	276.8	272.6	274.7	2.14
rmD1L3	63.1		63.1	0

Figure 8A: DNase activity measured by HCA / $Ca^{2+}Mg^{2+}$							
	pH-value	Experiment 1 [kU/nmol]		Experiment 2 [kU/nmol]		Mean [kU/nmol]	Std. ±
		Replicate 1	Replicate 2	Replicate 1	Replicate 2		
DNase1	5.0	0.00	0.26	0.00	0.14	0.10	0.13
	5.5	0.63	0.62	0.65	0.71	0.65	0.04
	6.0	2.35	2.06	2.62	2.50	2.38	0.24
	6.5	9.11	9.90	9.37	9.34	7.32	2.25
				*5.58	*5.40		
				*4.82	*5.07		
	7.0	15.53	14.69	12.82	12.73	12.03	2.17
				*10.84	*10.25		
				*9.97	*9.41		
	7.5	18.66	17.81	12.23	12.22	15.58	2.52
				*15.22	*16.52		
				*16.01	*16.01		
	8.0	14.24	12.97	10.45	10.49	12.04	1.88
	8.5	9.11	8.81	7.81	7.99	8.43	0.63
	9.0	5.13	5.21	4.30	4.48	4.78	0.46
DNase1l2	5.0	0.05	0.21	0.13	0.16	0.14	0.07
	5.5	0.59	0.37	0.36	0.40	0.43	0.11
	6.0	1.44	1.88	1.24	1.64	1.55	0.27
	6.5	2.67	3.05	2.82	2.45	2.75	0.25
	7.0	2.09	1.94	2.01	2.00	2.01	0.06
	7.5	0.98	1.10	1.51	1.09	1.17	0.24
	8.0	0.37	0.69	0.46	0.34	0.46	0.16
	8.5	0.18	0.23	0.18	0.33	0.23	0.07
	9.0	0.23	0.13	0.12	0.14	0.15	0.05
DNase1l3	5.0	0.00	0.14	0.26	0.05	0.11	0.12
	5.5	0.35	0.40	0.20	0.13	0.27	0.12
	6.0	0.33	0.61	0.59	0.67	0.55	0.15
	6.5	0.67	0.97	1.26	1.12	1.00	0.25
	7.0	1.43	1.21	1.18	1.11	1.23	0.14
	7.5	1.64	1.62	1.38	1.43	1.52	0.13
	8.0	1.90	1.92	1.81	1.74	1.84	0.08
	8.5	1.63	1.81	1.56	1.65	1.66	0.10
	9.0	1.20	1.21	1.09	0.97	1.12	0.12

*additional replicates

Figure 8B: DNase activity measured by HCA / $Ca^{2+}Co^{2+}$							
	pH-value	Experiment 1 [kU/nmol]				Mean [kU/nmol]	Std. ±
		Replicate 1	Replicate 2	Replicate 1	Replicate 2		
DNase1	5.0	0.23	0.05			0.14	0.13
	5.5	1.23	1.40			1.32	0.12
	6.0	2.96	3.93			3.45	0.68
	6.5	5.98	7.06			6.52	0.76
	7.0	7.21	8.12			7.67	0.64
	7.5	5.75	6.14			5.94	0.28
	8.0	2.88	3.53			3.20	0.46
	8.5	0.00	0.00			0.00	0.00
	9.0	0.00	0.00			0.00	0.00
DNase1l2	5.0	0.00	0.15			0.07	0.10
	5.5	0.52	0.43			0.48	0.07
	6.0	0.98	0.69			0.84	0.20
	6.5	1.06	0.85			0.95	0.15
	7.0	1.20	1.66			1.43	0.33
	7.5	0.99	0.89			0.94	0.07
	8.0	0.36	0.38			0.37	0.02
	8.5	0.00	0.00			0.00	0.00
	9.0	0.00	0.00			0.00	0.00
DNase1l3	5.0	0.02	0.04			0.03	0.02
	5.5	0.56	0.56			0.56	0.00
	6.0	1.13	0.97			1.05	0.11
	6.5	1.64	1.27			1.46	0.27
	7.0	1.46	1.65			1.55	0.13
	7.5	0.95	0.88			0.91	0.05
	8.0	0.18	0.22			0.20	0.03
	8.5	0.00	0.00			0.00	0.00
	9.0	0.00	0.00			0.00	0.00

Figure 8C: DNase activity measured by HCA / $Ca^{2+}Mn^{2+}$							
	pH-value	Experiment 1 [kU/nmol]		Experiment 2 [kU/nmol]		Mean [kU/nmol]	Std. ±
		Replicate 1	Replicate 2	Replicate 1	Replicate 2		
DNase1	5.0	0.45	0.52	0.43	0.40	0.45	0.05
	5.5	0.55	0.51	0.48	0.50	0.51	0.03
	6.0	1.06	0.89	0.99	0.83	0.94	0.10
	6.5	2.45	2.63	2.04	2.20	2.33	0.26
	7.0	5.29	5.34	5.17	5.01	5.20	0.15
	7.5	10.89	10.41	10.11	10.37	10.44	0.32
	8.0	14.48	14.11	13.09	13.73	13.85	0.59
	8.5	11.87	11.24	10.42	11.19	11.18	0.60
	9.0	6.04	5.64	5.00	5.19	5.47	0.47
DNase1l2	5.0	0.29	0.29	0.11	0.41	0.28	0.13
	5.5	0.77	0.83	0.74	0.57	0.73	0.11
	6.0	2.15	2.69	1.87	2.06	2.19	0.35
	6.5	4.51	4.09	3.78	3.83	4.06	0.33
	7.0	4.94	4.99	4.27	4.49	4.67	0.35
	7.5	5.14	5.24	4.70	4.83	4.98	0.25
	8.0	3.87	3.63	3.33	3.25	3.52	0.29
	8.5	1.65	1.73	1.71	1.64	1.68	0.05
	9.0	0.00	0.20	0.52	0.48	0.30	0.25
DNase1l3	5.0	0.19	0.07	0.12	0.23	0.15	0.07
	5.5	0.78	0.71	1.01	1.00	0.87	0.15
	6.0	1.79	2.23	2.74	2.79	2.39	0.47
	6.5	4.11	3.80	4.58	4.60	4.27	0.39
	7.0	4.29	3.51	5.64	5.46	4.72	1.01
	7.5	4.48	5.08	6.21	6.31	5.52	0.89
	8.0	3.70	3.75	4.71	5.12	4.32	0.71
	8.5	2.11	2.04	2.74	2.96	2.46	0.46
	9.0	0.86	0.45	0.47	0.21	0.50	0.27

**Figure 10A: Inhibition of actin polymerization by DNase1**			
	Fluorescence [arbitrary units]		
Time [sec]	α-actin	α-actin + rmDNase1	α-actin + rhDNase1
10	28,047333	20,568333	21,168666
20	28,063333	20,741333	21,239666
30	28,196333	20,867000	20,985000
40	29,256333	20,688666	20,442333
50	30,597000	20,371333	20,038666
60	32,027666	19,962333	19,975666
70	33,587000	19,663333	19,847000
80	35,398666	19,739333	19,954333
90	37,293000	20,125000	20,027666
100	39,142999	20,403000	20,172000
110	41,040666	20,232666	20,172000
120	42,972333	19,986000	20,356000
130	45,249333	19,912666	20,387666
140	47,400666	19,878666	20,584333
150	49,655333	19,810666	20,502666
160	51,636000	19,776666	20,358333
170	53,589666	19,839666	19,981000
180	55,495000	20,109666	19,813333
190	57,578000	20,306333	19,676666
200	59,755999	20,476666	19,616000
210	61,941666	20,405666	19,752333
220	63,760000	20,295333	19,852333
230	65,544333	20,329333	19,991333
240	67,474666	20,334666	19,786666
250	69,319333	20,584000	19,692000
260	71,291333	20,746000	19,548000
270	72,750999	20,845666	19,589666
280	74,468666	20,871333	19,681666
290	76,042999	20,879666	19,718333
300	77,798666	21,010666	19,635000
310	79,294666	21,147000	19,572000
320	80,635666	21,217666	19,572000
330	81,864333	21,285666	19,645333
340	83,171666	21,191666	19,572000
350	84,410999	21,044666	19,474666
360	85,594333	21,010666	19,316666
370	86,664000	20,863666	19,258333
380	88,211666	20,900333	19,413333
390	89,258000	20,871666	19,576333
400	90,646000	20,840333	19,720666

410	91,736333	20,777666	19,733666
420	93,264333	20,675333	19,623666
430	94,310000	20,842666	19,469333
440	95,085333	20,979000	19,437666
450	96,004000	21,146666	19,584333
460	97,053333	21,262666	19,752333
470	98,218333	21,427333	19,606000
480	99,105999	21,498333	19,527666
490	99,972666	21,524000	19,457000
500	100,821666	21,566666	19,651000
510	102,043000	21,629333	19,687666
520	103,204666	21,655000	19,724333
530	104,023666	21,609666	19,666666
540	104,570333	21,467666	19,493666
550	105,061666	21,345333	19,527666
560	106,367000	21,217333	19,522333
570	107,369666	21,259666	19,760666
580	108,600666	21,217333	19,729000
590	109,451000	21,143666	19,802333
600	110,249666	21,070000	19,763333
610	111,099000	21,041666	19,826333
620	111,844666	21,010333	19,758333
630	112,683000	20,979333	19,766000
640	113,185666	21,005000	19,842000

**Figure 10B: DNase1 inhibition by α-actin**					
HCA, Ca/Mg, pH 7.0					
	rmDNase1				
Molar ratio α-actin : DNase1	Replicate 1 [kU/nmol]	Replicate 2 [kU/nmol]	Replicate 3 [kU/nmol]	Mean [kU/nmol]	Std. ±
0	10,9	10,6	9,4	10,3	0,64
0.5	5,9	6,2	4,7	5,6	0,68
1.0	5,4	3,8	5,4	4,9	0,75
2.0	3,8	3,6		3,7	0,12
3.0	3,8	3,0		3,4	0,38
	rhDNase1				
0	3,3	3,1	3.7	3,4	0,22
0.5	1,5	1,4		1,5	0,07
1.0	0,6	0,8		0,7	0,10
2.0	0,6	0,3		0,5	0,19
3.0	0,0	0,1		0,1	0,04

**S4A Figure: Inhibition of actin polymerization by DNases**					
	Fluorescence [arbitarry units]				
Time [sec]	α-actin Ctrl	α-actin + rhDNase1	α-actin + rmDNase1	α-actin + rmDNase1l2	α-actin + rmDNase1l3
10	10,617000	11,118000	10,597000	11,293000	10,178000
20	10,462333	11,055666	10,631666	10,993000	10,011666
30	10,399666	11,054333	10,463666	10,790333	9,910666
40	10,431666	11,118333	10,430333	10,654333	9,914333
50	10,394333	11,112666	10,461000	10,683666	10,009000
60	10,500000	11,141666	10,797000	10,681000	9,948666
70	10,533333	11,111333	10,962333	10,747666	9,916666
80	10,529333	11,008333	11,024666	10,745000	9,914000
90	10,390333	10,911333	10,855333	10,714333	10,078000
100	10,358333	10,875000	10,790000	10,613000	10,043666
110	10,263333	10,982000	10,765000	10,586333	9,947999
120	340,153333	10,912333	10,899666	10,621000	9,817333
130	423,091000	10,809333	10,997000	10,657000	9,818333
140	428,328000	10,737000	11,025000	10,557000	9,754000
150	102,677333	10,908000	10,984666	10,518333	9,816666
160	23,383333	11,008333	10,990333	10,549000	9,784666
170	21,590666	11,079333	11,093000	10,647666	9,746666
180	20,624666	11,011333	11,265333	275,695666	9,611333
190	20,056333	10,911000	11,327666	396,310333	9,675666
200	19,824666	160,163666	307,762666	398,931666	339,818000
210	19,533666	162,223333	498,385666	135,509000	395,954666
220	19,672666	162,422000	498,391333	16,331000	397,052333
230	19,773000	13,233333	202,026000	15,408000	67,349000
240	19,968666	11,339000	11,702666	15,848333	11,543333
250	19,968666	11,272333	12,126666	16,410333	10,742000
260	20,002333	11,233333	12,189000	16,679000	10,670000
270	20,264666	11,066666	12,092333	16,679000	10,805666
280	20,588333	11,097333	11,864000	16,548000	10,840333
290	20,951000	11,268333	11,965333	16,483333	11,044000
300	21,154333	11,269666	12,032000	16,490000	11,081666
310	21,296333	11,242000	12,036666	16,718666	11,284333
320	21,605333	11,113000	12,231666	17,092000	11,481000
330	22,079000	11,115666	12,361666	17,420333	11,850333
340	22,474333	11,182333	12,688666	17,717666	12,152666
350	22,864333	11,146000	12,628666	17,775333	12,488666
360	23,223333	11,217000	12,533333	17,775333	12,817000
370	23,654666	11,153000	12,468333	17,789000	13,080333
380	24,019000	11,183333	12,533333	17,753333	13,275000

390	24,389666	11,272000	12,636666	17,891333	13,431000
400	24,736333	11,268000	12,568333	17,944000	13,625333
410	25,210666	11,239000	12,473000	18,310333	13,922666
420	25,628666	11,105666	12,503000	18,576666	14,188333
430	26,147000	11,172333	12,538000	19,019000	14,528000
440	26,534333	11,236333	12,669666	19,516000	14,729333
450	27,045333	11,342000	12,770000	20,085000	14,934333
460	27,616666	11,410000	12,736666	20,684666	15,323666
470	28,076666	11,349000	12,700333	20,951000	15,720666
480	28,444000	11,349000	12,600000	21,309333	16,242000
490	28,833666	11,315666	12,703333	21,673000	16,414666
500	29,559666	11,376666	12,805000	22,172333	16,749333
510	30,253333	11,375333	12,937000	22,626666	17,119000
520	30,928666	11,336333	13,035333	23,114000	17,546666
530	31,344666	11,336333	13,133666	23,694666	18,012000
540	31,778666	11,368333	13,198666	24,220000	18,353666
550	32,333666	11,272666	13,298666	24,742333	18,852333
560	32,876333	11,237666	13,230333	25,273666	19,251333
570	33,606000	11,136000	13,097000	25,718666	19,617000
580	34,290666	11,301000	13,028666	26,293666	20,123333
590	35,012000	11,334333	13,137000	26,935333	20,483666
600	35,574666	11,402666	13,273666	27,567333	20,941333
610	36,095333	11,268000	13,277000	28,066000	21,426333
620	36,695666	11,239000	13,373666	28,376333	22,118333
630	37,410666	11,140333	13,442000	29,041666	22,677333
640	38,121000	11,279666	13,543666	29,737666	22,985333
650	38,821666	11,345000	13,554000	30,528666	23,096666
660	39,494666	11,445000	13,620666	31,190000	23,450000
670	40,252333	11,414333	13,687333	31,795000	23,915333
680	41,024999	11,279666	13,710333	32,451666	24,544000
690	41,731666	11,308666	13,673666	33,112333	25,070000
700	42,510000	11,305666	13,626666	33,831000	25,705000
710	43,211000	11,439000	13,619666	34,471333	26,200333
720	44,190666	11,473666	13,682666	35,007666	26,762333
730	44,902000	11,502666	13,791333	35,401666	27,055000
740	45,469333	11,502666	13,828333	36,224000	27,501000
750	46,136333	11,469333	13,932000	36,846000	27,955666
760	46,888000	11,400000	13,924666	37,458000	28,609333
770	47,777000	11,395666	14,057666	37,786333	29,267333
780	48,263000	11,265333	14,087333	38,347666	29,873000
790	48,761666	11,339333	14,128000	39,240333	30,378666
800	49,428666	11,345000	14,161333	39,638000	30,799000
810	50,117000	11,445000	14,200000	40,183666	31,135333
820	50,826666	11,543333	14,331333	40,633333	31,600333
830	51,313333	11,476666	14,326000	41,486666	32,223333
840	51,987666	11,442000	14,355666	42,146000	32,959000

850	52,708333	11,275333	14,355666	42,599333	33,548666
860	53,516666	11,343333	14,424000	43,163666	34,039000
870	53,943666	11,311333	14,485000	43,551666	34,648666
880	54,310666	11,308333		43,967333	35,240000
890	54,530333	11,337333		44,294333	36,038333
900	55,178999	11,437333		44,671000	36,626333
910	55,560333	11,505666		45,385666	37,197000
920	56,216333	11,508666		45,968999	37,539333
930	56,736000	11,443333		46,818000	38,133000
940	57,370333	11,314000		47,250333	38,722000
950	58,145333			47,777000	39,367333
960	58,697666			48,149000	39,871333
970	59,342666			48,581333	40,498333
980	59,654999			49,060000	41,007666
990	60,085999			49,465999	41,723000
1000	60,527333			49,757333	42,111000
1010	61,238333			50,251333	42,604333
1020	61,749000			50,571333	42,881333
1030	62,441666			50,858000	43,562333
1040	62,774000			51,044000	44,149666
1050	63,349333			51,543333	44,521000
1060	63,632333			52,195666	44,748666
1070	64,166999			52,794666	45,170666
1080	64,617666			53,333666	45,581333
1090	65,051666			53,786333	46,373333
1100	65,476999			54,299333	46,872333
1110	65,968999			54,665666	47,486666
1120	66,803333			55,157999	47,864666
1130	67,128666			55,331333	48,403333
1140	67,603999			55,671333	48,721000
1150	67,913333			56,032666	49,020333
1160	68,507000			56,566000	49,393000
1170	68,900333			56,999000	50,046000
1180	69,234333			57,469666	50,590999
1190	69,857666			57,895333	50,937666
1200	70,246666			58,428000	51,250333
1210	70,687000			58,898000	51,703000
1220	70,887333			59,364000	51,975666
1230	71,563333			59,697000	52,268333
1240	72,411999			59,941000	52,527666
1250					53,146333
1260					53,759333
1270					54,239000
1280					54,538000
1290					55,030333
1300					55,508000

**S4B Figure: DNase1 inhibition by α-actin**				
HCA, Ca/Mn, pH 7.0				
	Ctrl	DNase/α-actin 1:1	DNase/α-actin 1:2	
	ΔOD260/min	ΔOD260/min	ΔOD260/min	
rhDNase1	0,5404	0,2129	0,0168	Replicate 1
	0,5820	0,2720	0,0100	Replicate 2
	0,5612	0,2425	0,0134	Mean
	0,0294	0,0418	0,0048	Std. ±
rmDNase1	0,3826	0,2891	0,2340	Replicate 1
	0,3139	0,2702	0,2657	Replicate 2
	0,4292	0,3548	0,3297	Replicate 3
	0,3752	0,3125	0,2977	Mean
	0,0580	0,0444	0,0488	Std. ±
rmDNase1l2	0,2627	0,2789	0,2760	Replicate 1
	0,2813	0,2707	0,2637	Replicate 2
	0,2720	0,2748	0,2699	Mean
	0,0132	0,0058	0,0087	Std. ±
rmDNase1l3	0,1466	0,1224	0,1498	Replicate 1
	0,1333	0,1344	0,1538	Replicate 2
	0,1400	0,1284	0,1518	Mean
	0,0094	0,0085	0,0028	Std. ±
HCA, Ca/Mg, pH 7.5				
rhDNase1	0,1759	0,0478	0,0000	Replicate 1
	0,1875	0,0608	0,0027	Replicate 2
	0,1817	0,0543	0,0014	Mean
	0,0082	0,0092	0,0019	Std. ±
rmDNase1	0,9189	0,4267	0,1979	Replicate 1
	0,8999	0,5927	0,2082	Replicate 2
	0,9094	0,5097	0,2031	Mean
	0,0134	0,1174	0,0073	Std. ±
HCA, Ca/Mg, pH 6.5				
rmDNase1l2	0,0555	0,0443	0,0555	Replicate 1
	0,0510	0,0529	0,0540	Replicate 2
	0,0533	0,0486	0,0548	Mean
	0,0032	0,0061	0,0011	Std. ±
HCA, Ca/Mg, pH 8.0				
rmDNase1l3	0,0254	0,0269	0,0273	Replicate 1
	0,0316	0,0294	0,0279	Replicate 2
	0,0285	0,0282	0,0276	Mean
	0,0044	0,0018	0,0004	Std. ±
