# Supplementary material for: Comparison of the secretory murine DNase1 family members expressed in Pichia pastoris
Source: PLoS One. 2021 Jul 30;16(7):e0253476. doi: 10.1371/journal.pone.0253476 (PMC8323900; doi:10.1371/journal.pone.0253476)
Supplement: S1 Raw images — (PDF) [file pone.0253476.s006.pdf]

S1_raw images

Figure 2

All pictures were taken with the ChemiDoc™ XRS+ System with the ImageLab 5.0 software (Bio-Rad Laboratories).

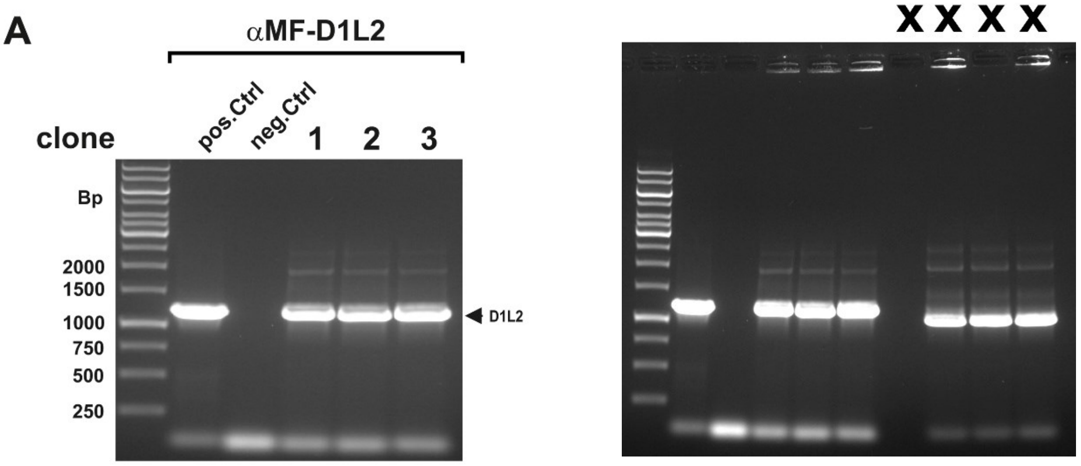

Marker: GeneRuler 1 kbp DNA Ladder (ThermoFisher Scientific)

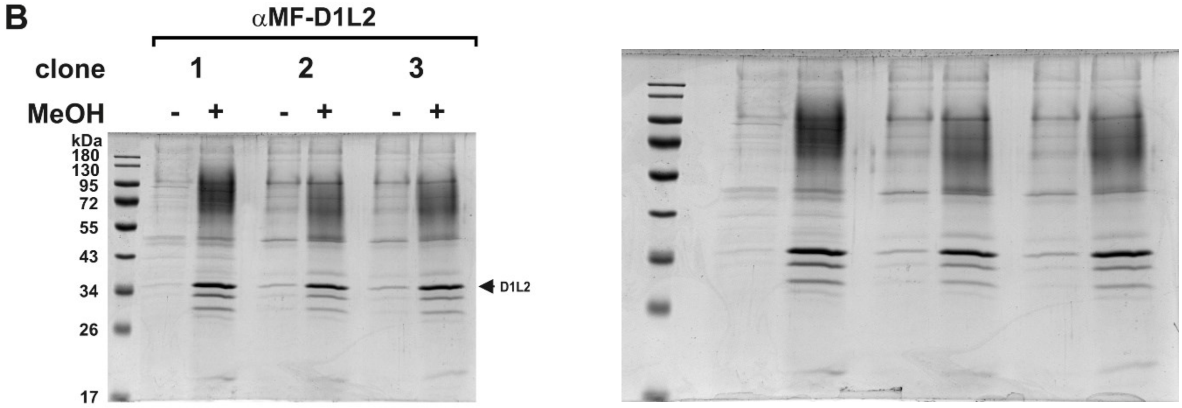

Marker: PageRuler Prestained Protein Ladder (ThermoFisher Scientific)

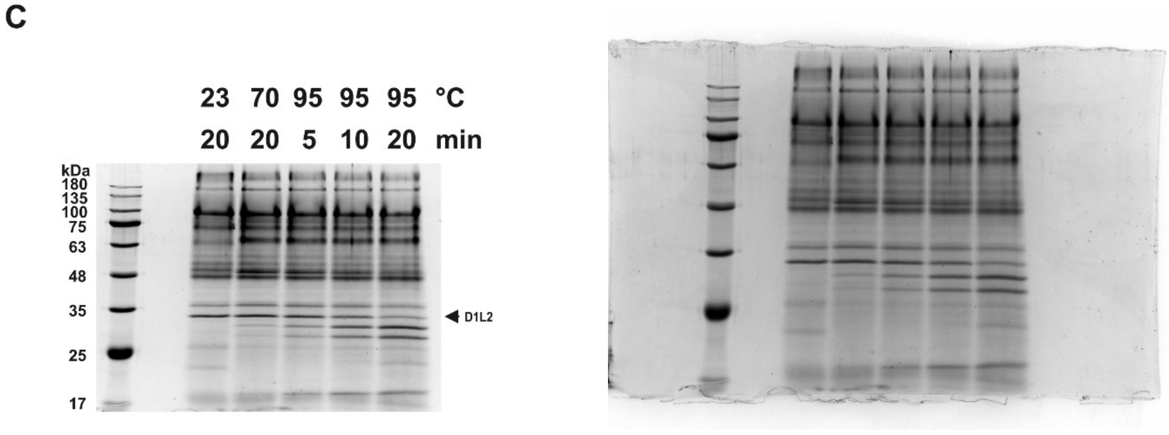

Marker: Cozy Prestained Protein Ladder (highQu)

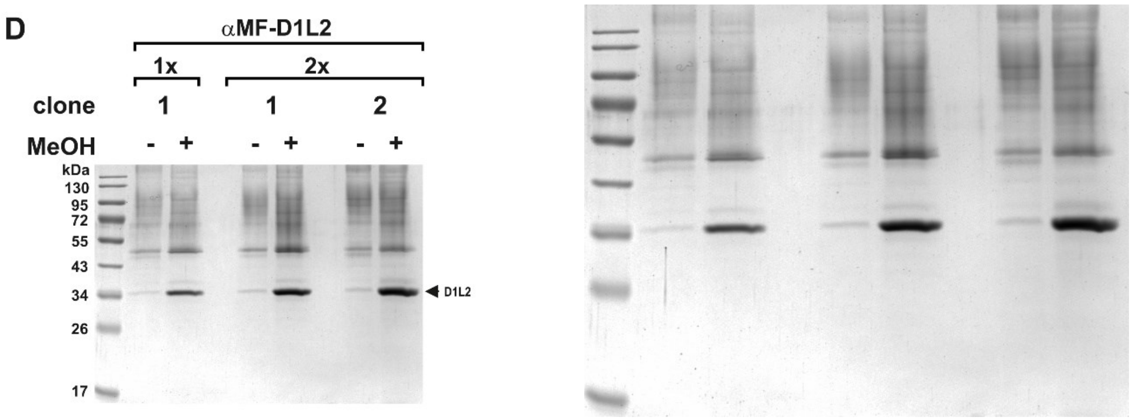

Marker: PageRuler Prestained Protein Ladder (ThermoFisher Scientific)

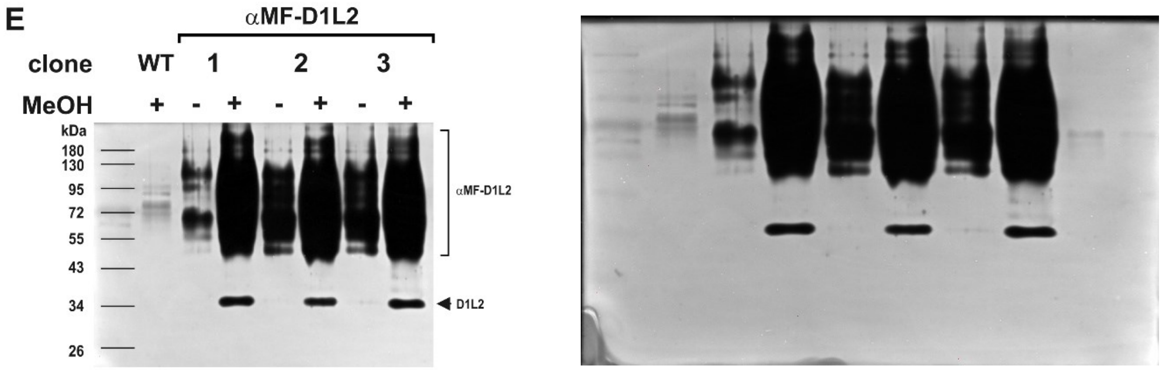

Marker: PageRuler Prestained Protein Ladder (ThermoFisher Scientific)

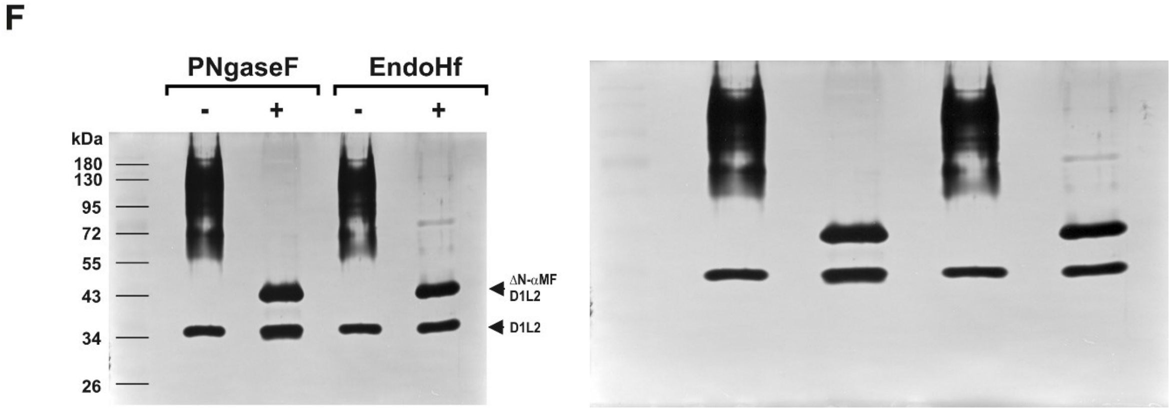

Marker: PageRuler Prestained Protein Ladder (ThermoFisher Scientific)

Figure 3

All pictures were taken with the ChemiDoc™ XRS+ System with the ImageLab 5.0 software (Bio-Rad Laboratories).

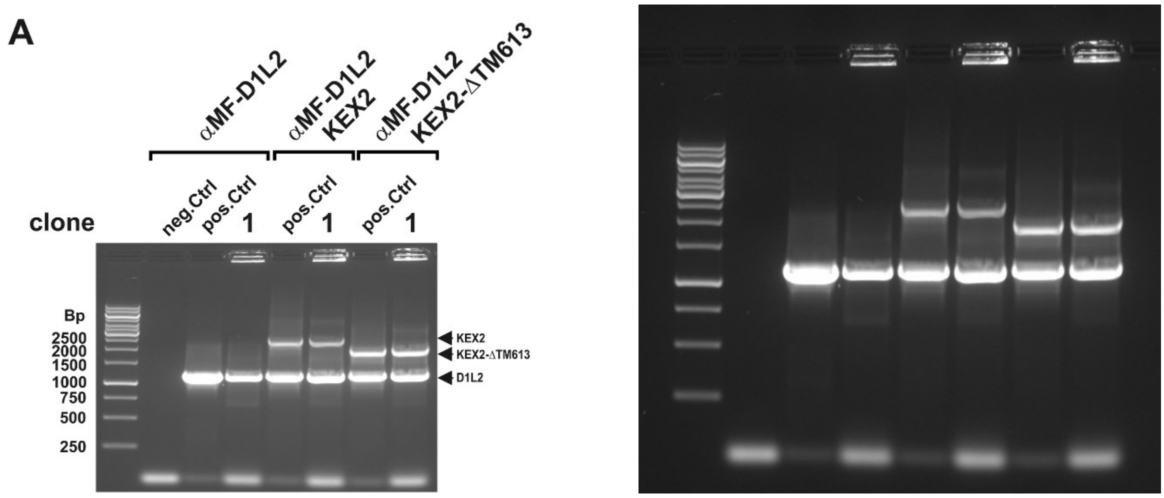

Marker: GeneRuler 1 kbp DNA Ladder (ThermoFisher Scientific)

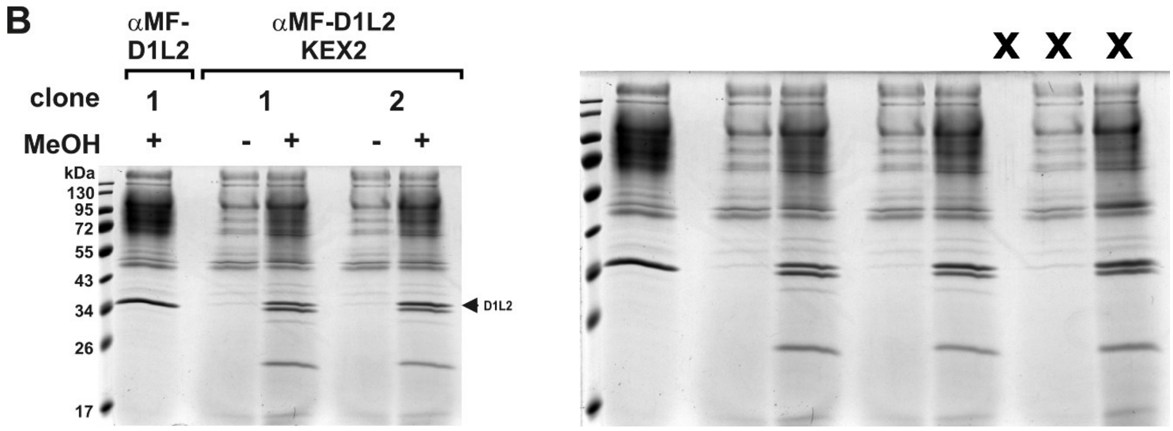

Marker: PageRuler Prestained Protein Ladder (ThermoFisher Scientific)

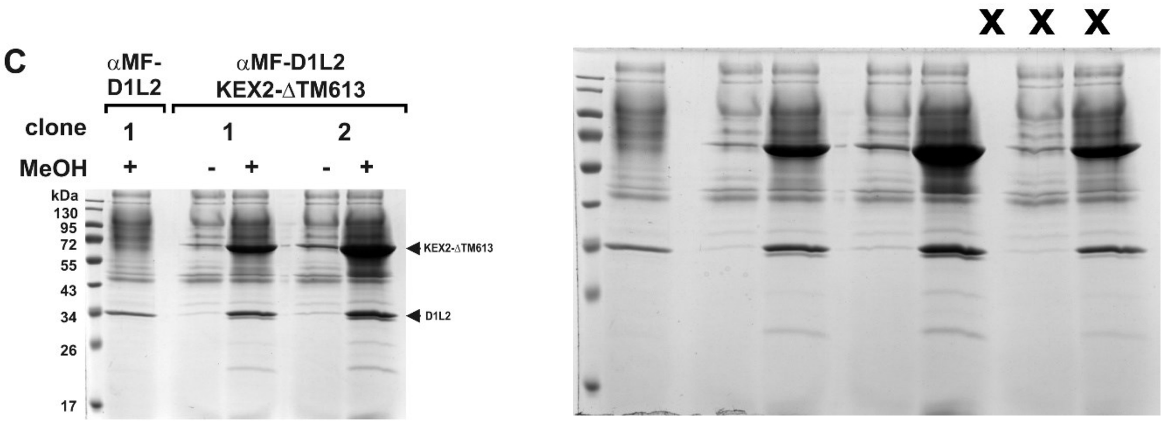

Marker: PageRuler Prestained Protein Ladder (ThermoFisher Scientific)

Figure 4

All pictures were taken with the ChemiDoc™ XRS+ System with the ImageLab 5.0 software (Bio-Rad Laboratories).

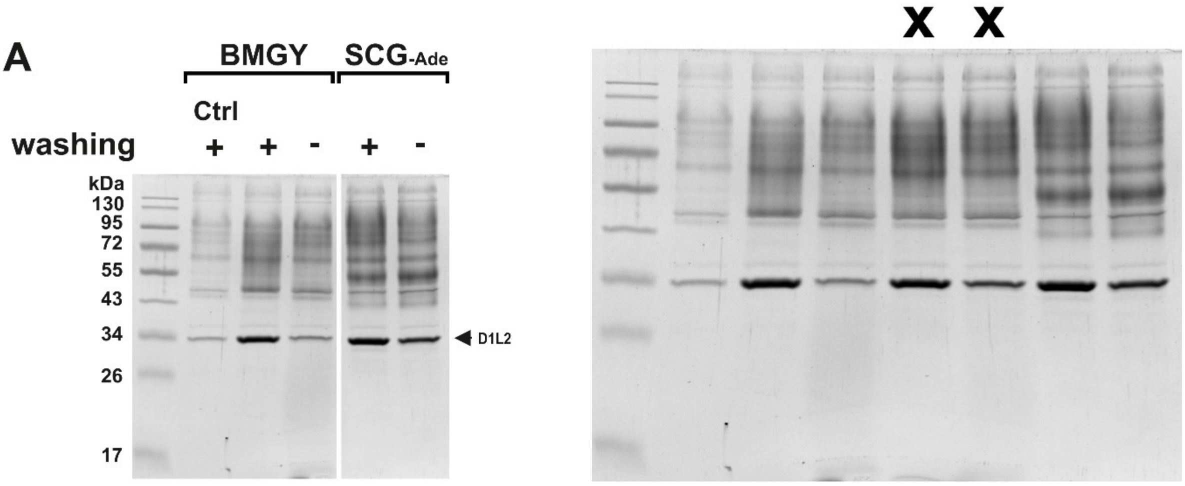

Marker: PageRuler Prestained Protein Ladder (ThermoFisher Scientific)

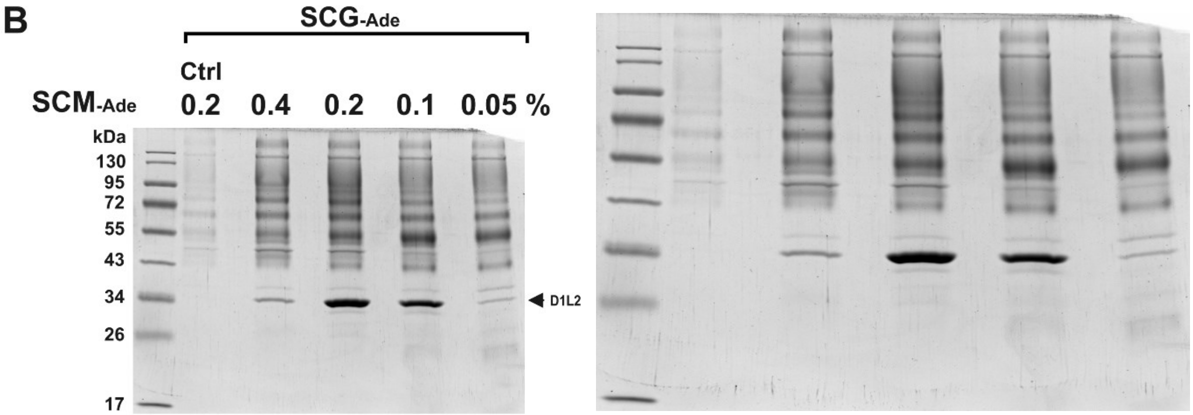

Marker: PageRuler Prestained Protein Ladder (ThermoFisher Scientific)

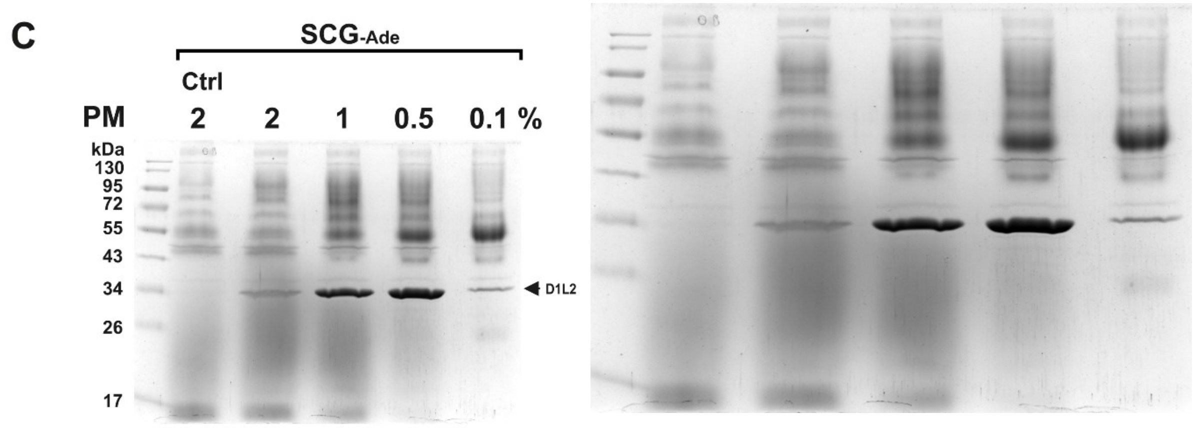

Marker: PageRuler Prestained Protein Ladder (ThermoFisher Scientific)

D

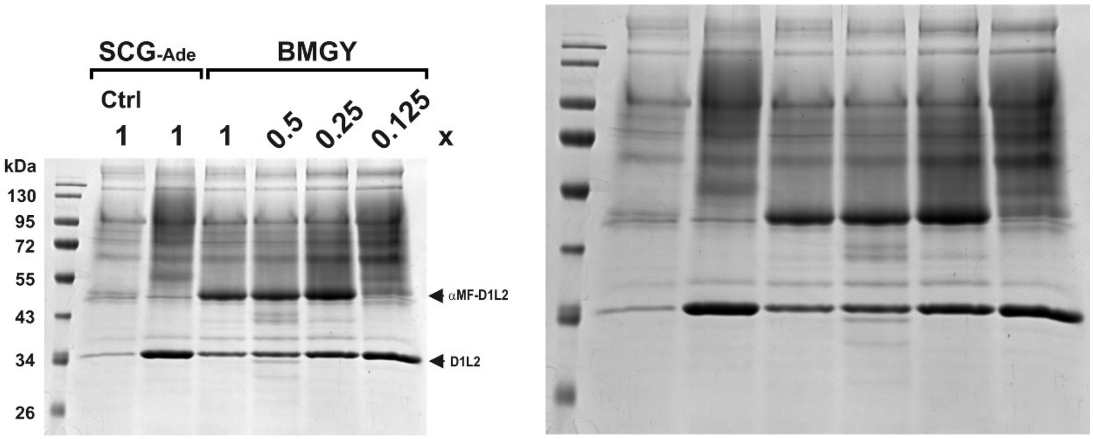

Marker: PageRuler Prestained Protein Ladder (ThermoFisher Scientific)

E

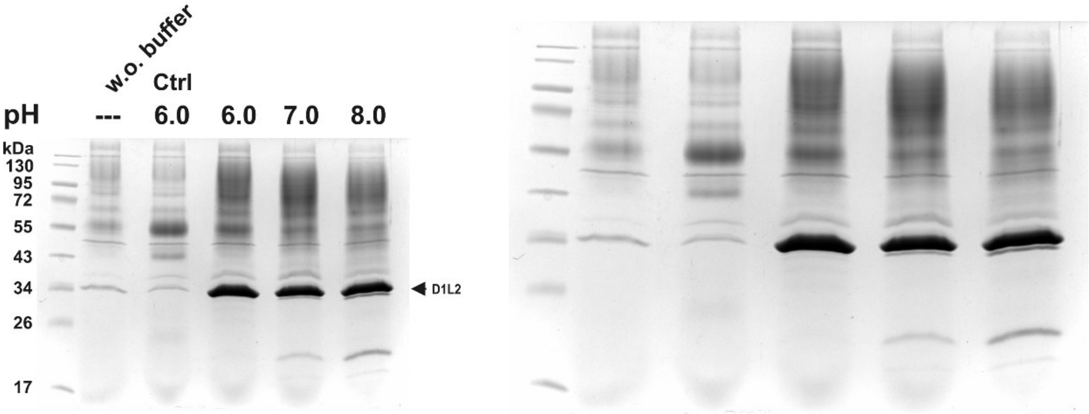

Marker: PageRuler Prestained Protein Ladder (ThermoFisher Scientific)

F

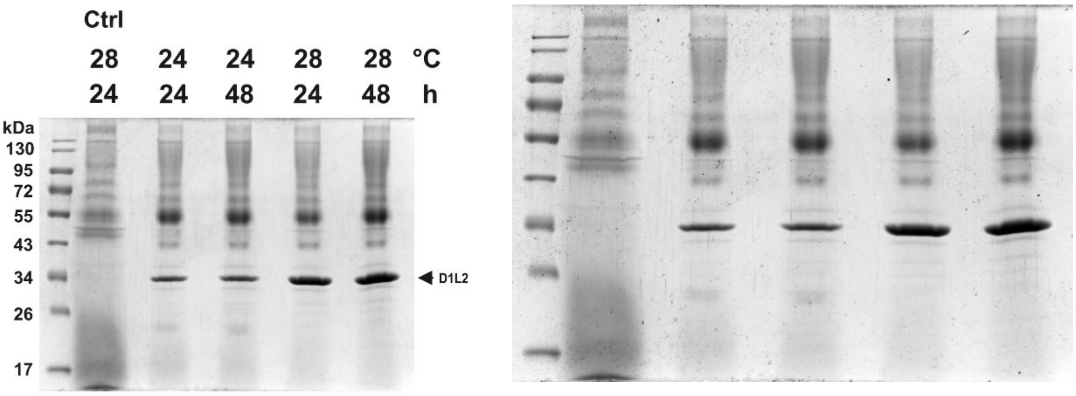

Marker: PageRuler Prestained Protein Ladder (ThermoFisher Scientific)

Figure 5

All pictures were taken with the ChemiDoc™ XRS+ System with the ImageLab 5.0 software (Bio-Rad Laboratories).

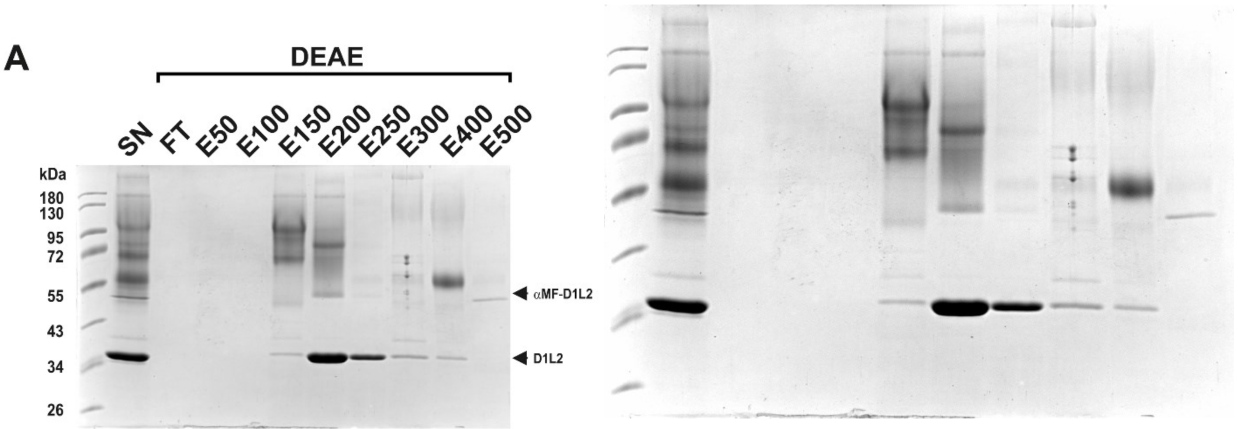

Marker: PageRuler Prestained Protein Ladder (ThermoFisher Scientific)

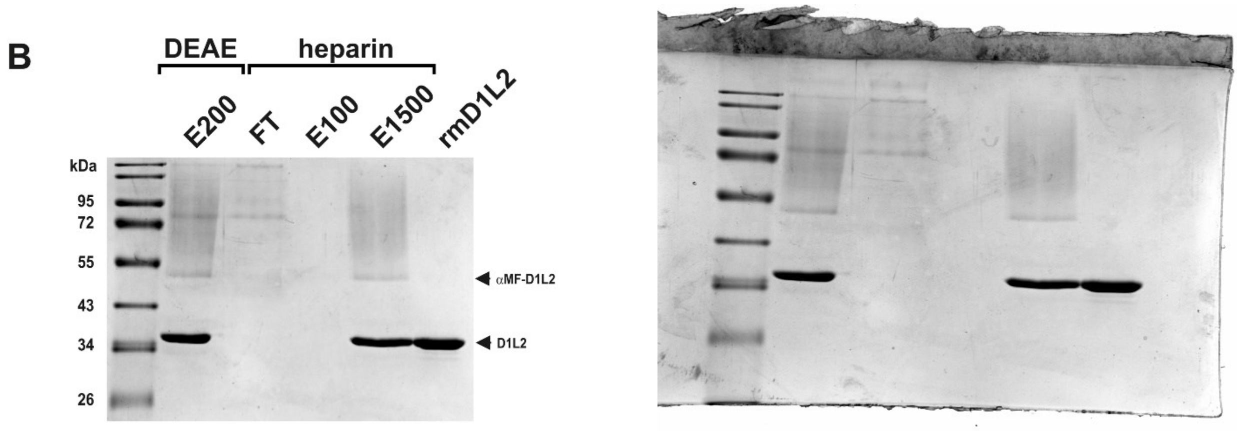

Marker: PageRuler Prestained Protein Ladder (ThermoFisher Scientific)

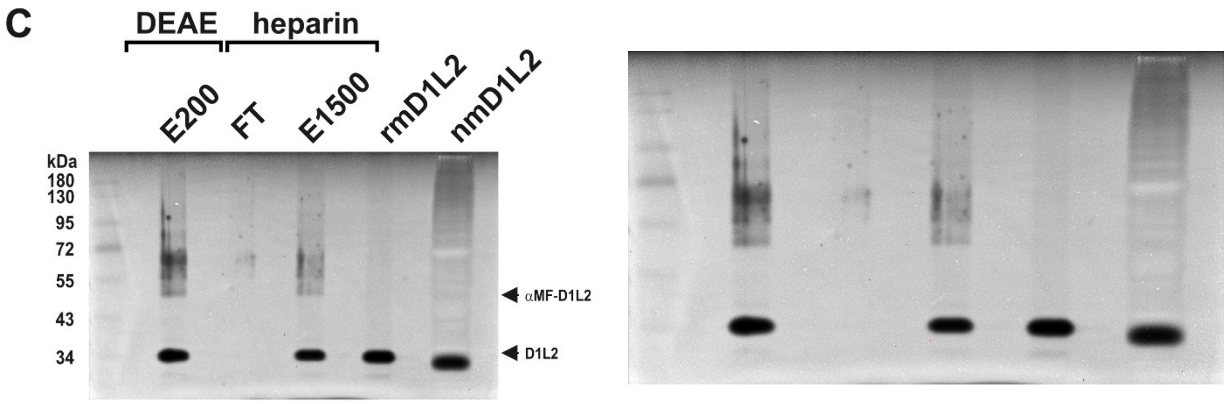

Marker: PageRuler Prestained Protein Ladder (ThermoFisher Scientific)

Figure 6

All pictures were taken with the ChemiDoc™ XRS+ System with the ImageLab 5.0 software (Bio-Rad Laboratories).

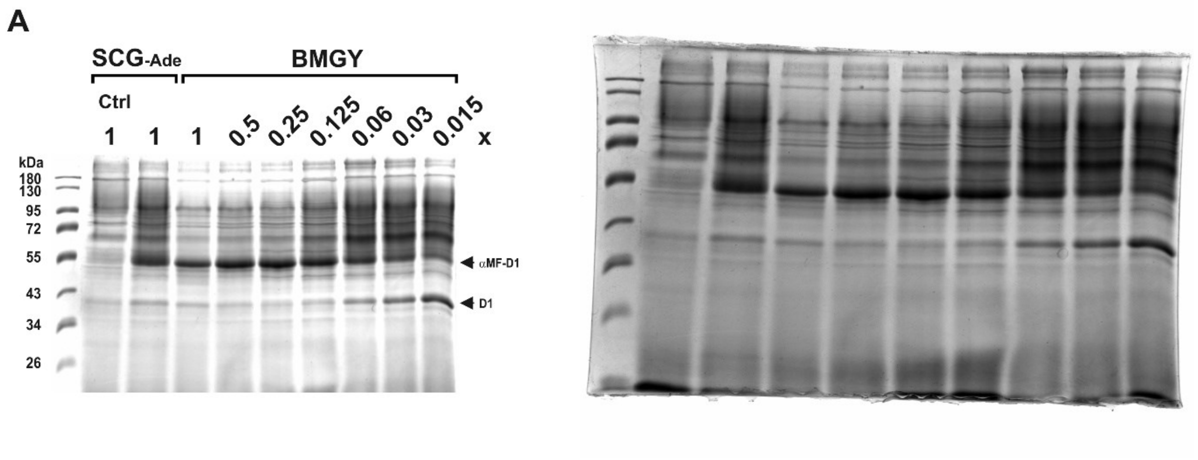

Marker: PageRuler Prestained Protein Ladder (ThermoFisher Scientific)

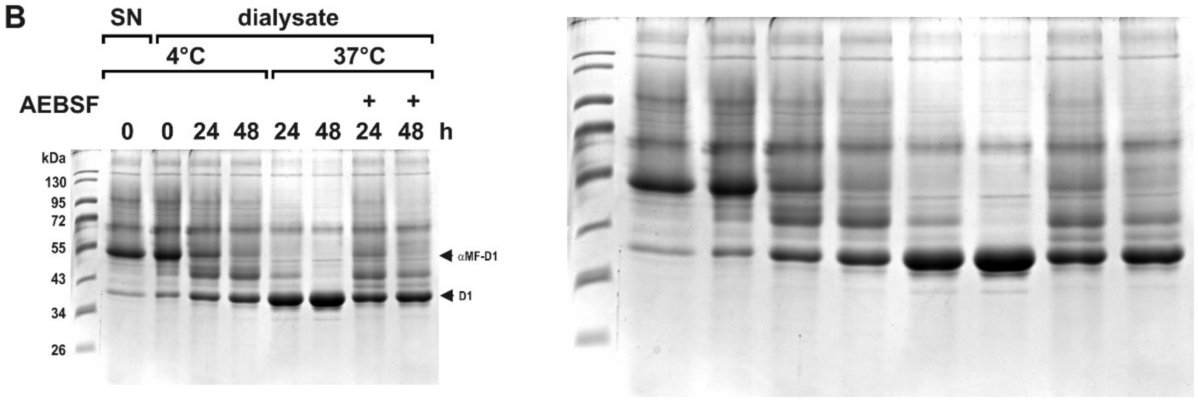

Marker: PageRuler Prestained Protein Ladder (ThermoFisher Scientific)

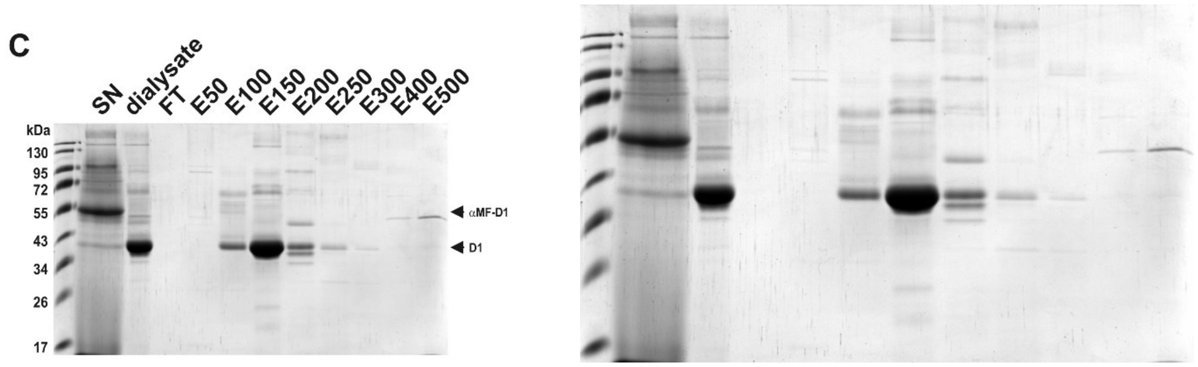

Marker: PageRuler Prestained Protein Ladder (ThermoFisher Scientific)

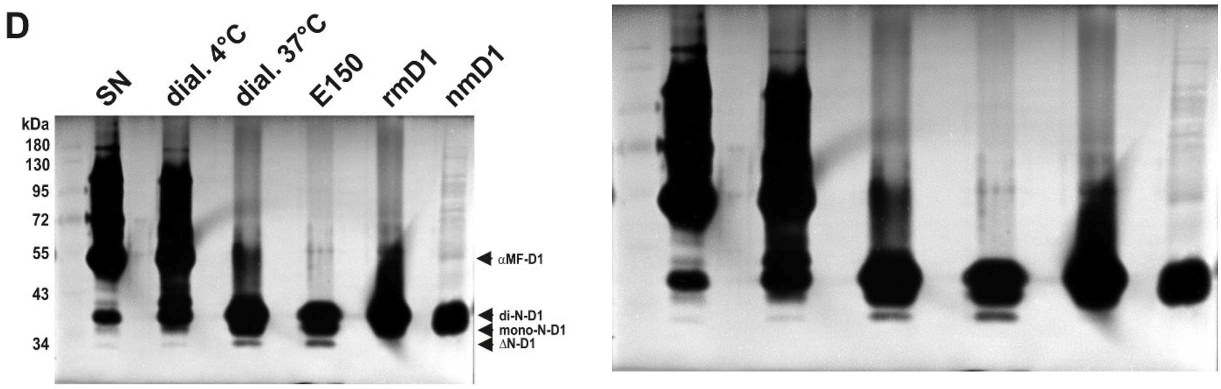

Marker: PageRuler Prestained Protein Ladder (ThermoFisher Scientific)

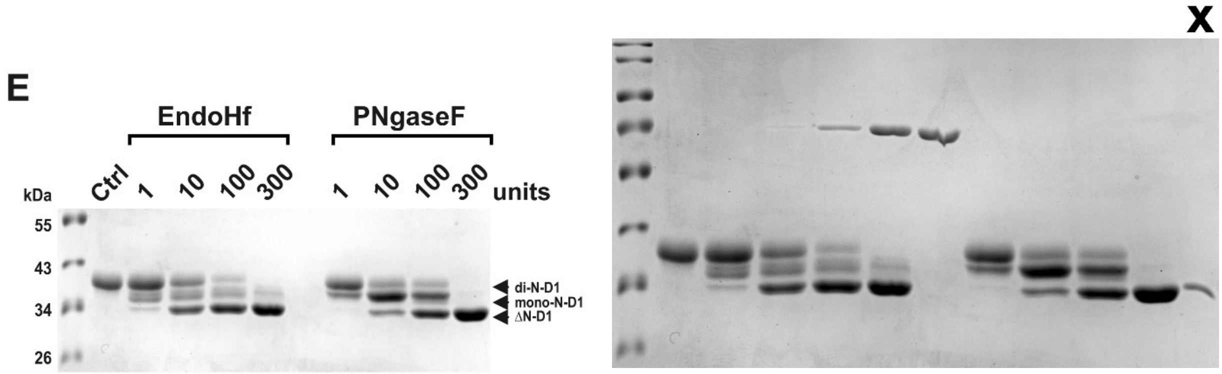

Marker: PageRuler Prestained Protein Ladder (ThermoFisher Scientific)

Figure 7

All pictures were taken with the ChemiDoc™ XRS+ System with the ImageLab 5.0 software (Bio-Rad Laboratories).

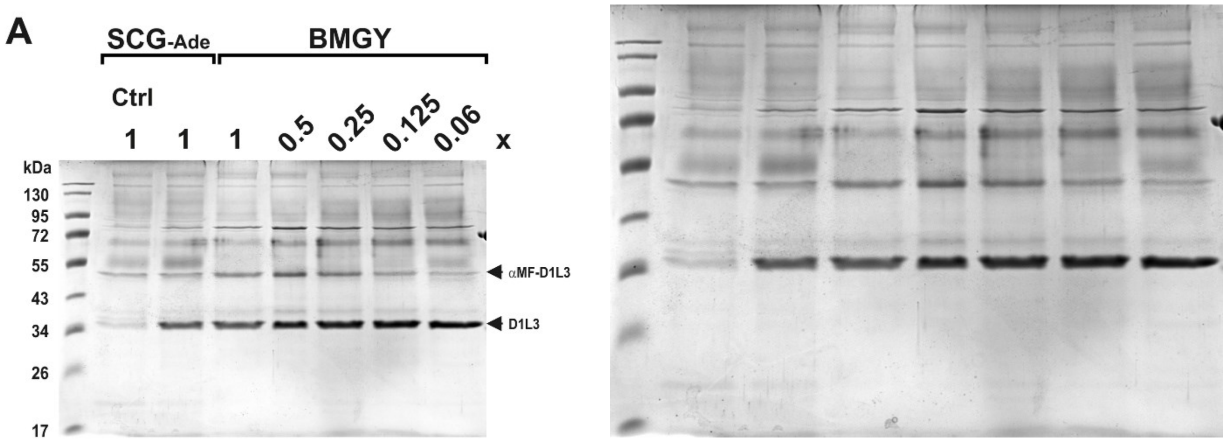

Marker: PageRuler Prestained Protein Ladder (ThermoFisher Scientific)

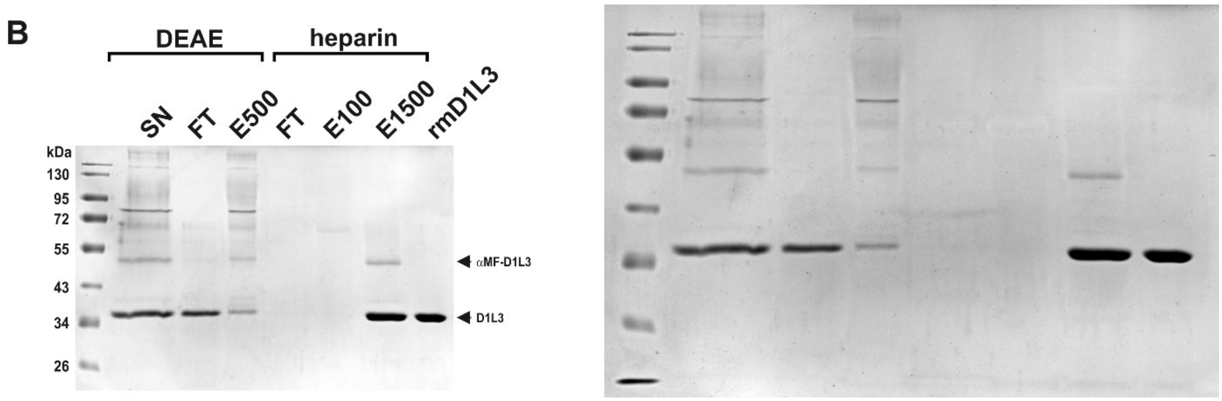

Marker: PageRuler Prestained Protein Ladder (ThermoFisher Scientific)

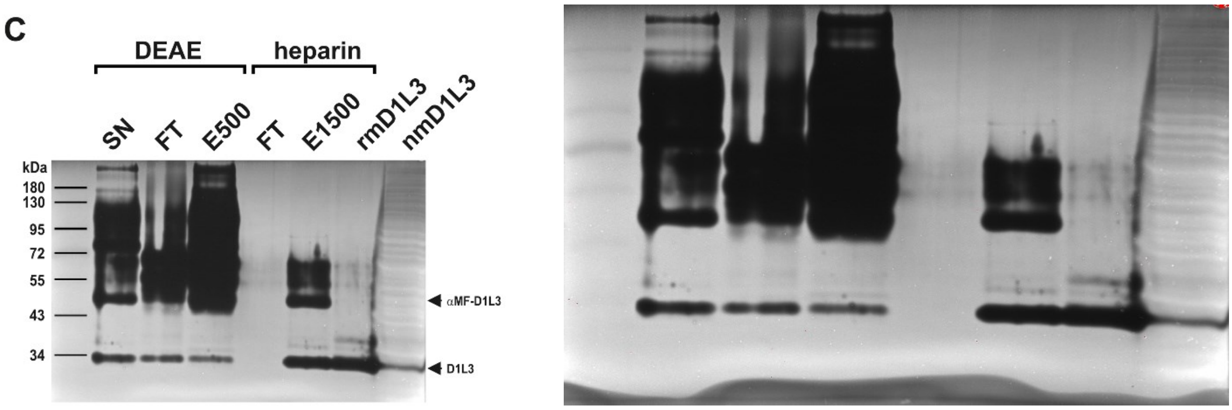

Marker: PageRuler Prestained Protein Ladder (ThermoFisher Scientific)

D

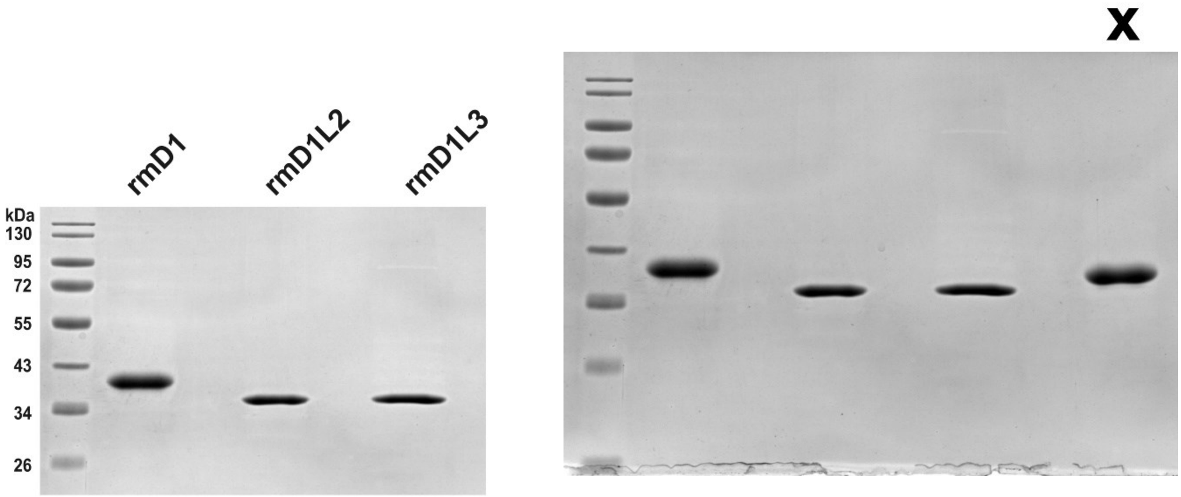

Marker: PageRuler Prestained Protein Ladder (ThermoFisher Scientific)

E

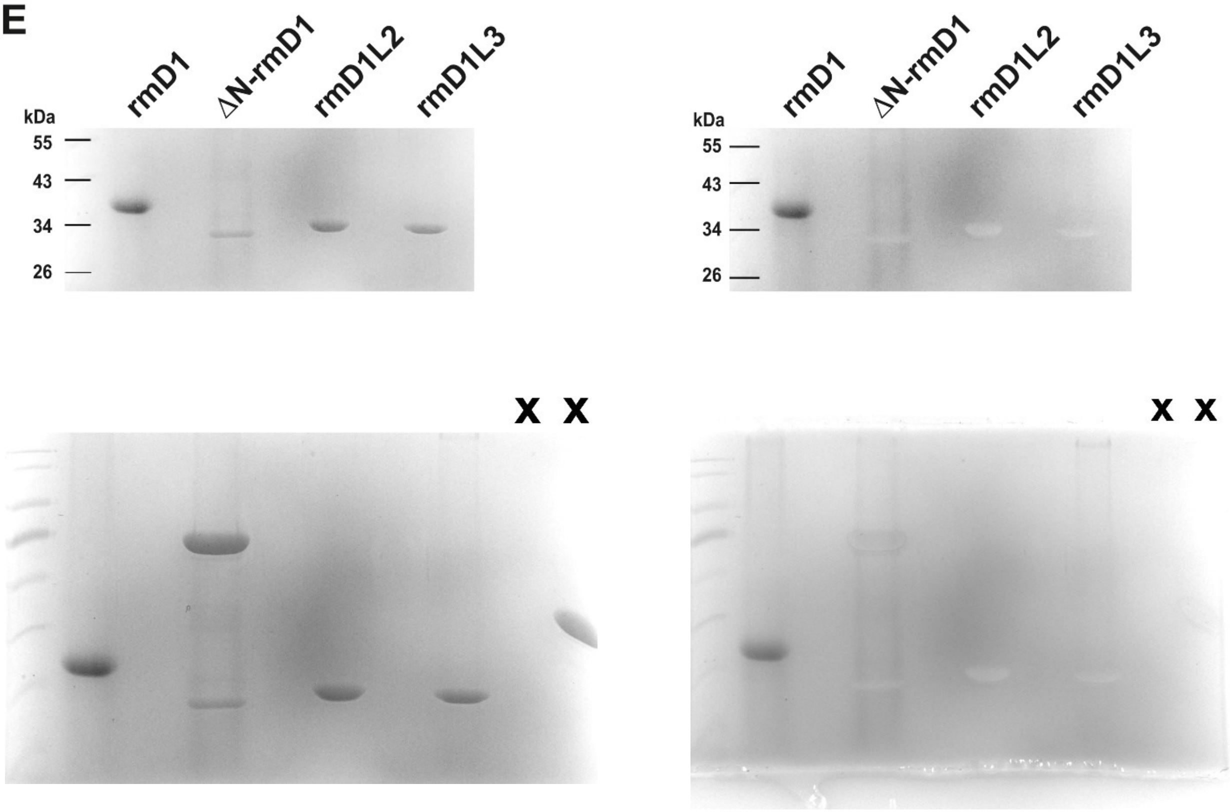

Marker: PageRuler Prestained Protein Ladder (ThermoFisher Scientific)

Figure 9

All pictures were taken with the ChemiDoc™ XRS+ System with the ImageLab 5.0 software (Bio-Rad Laboratories).

A

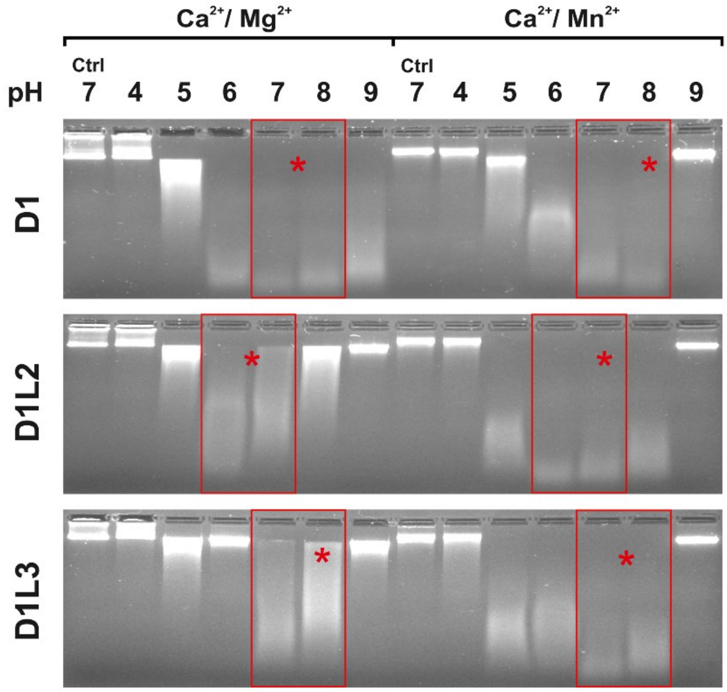

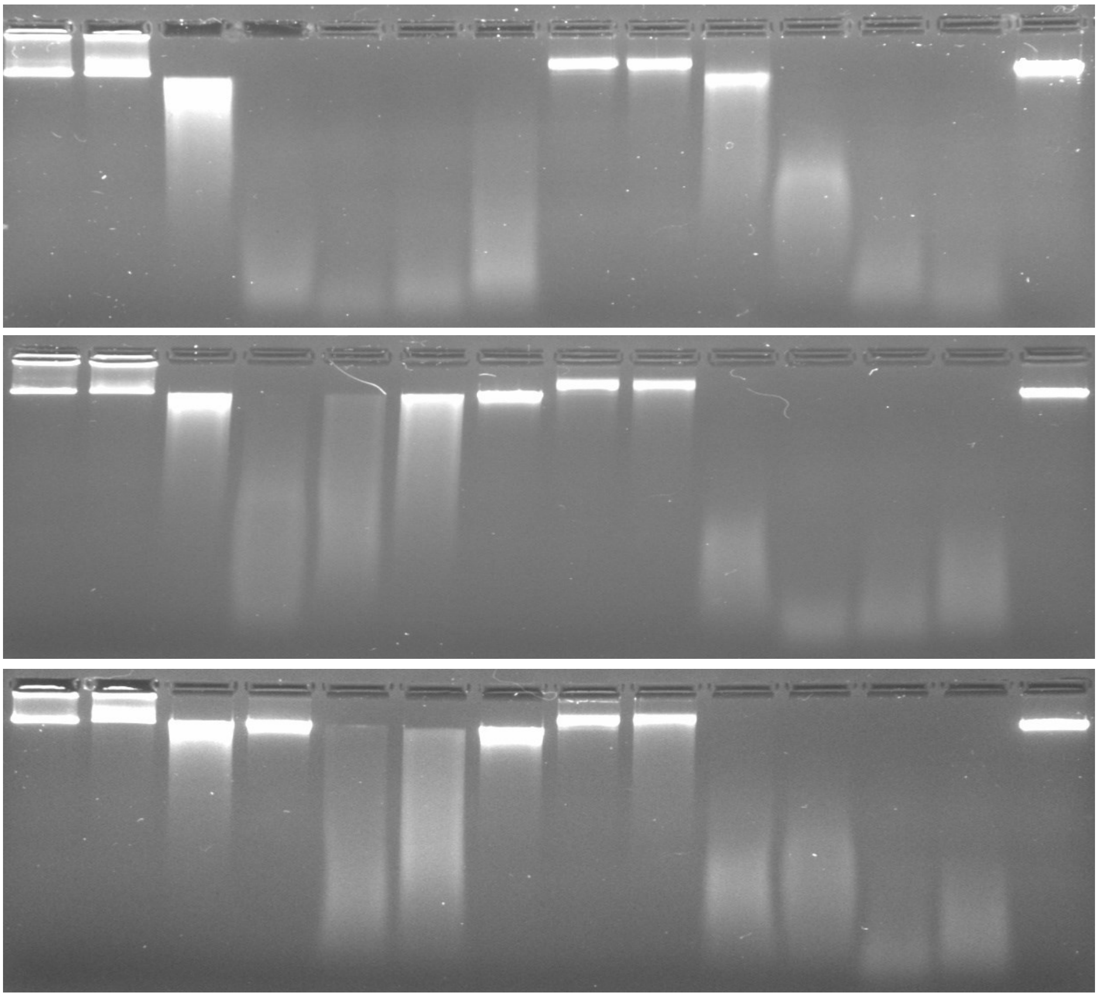

B

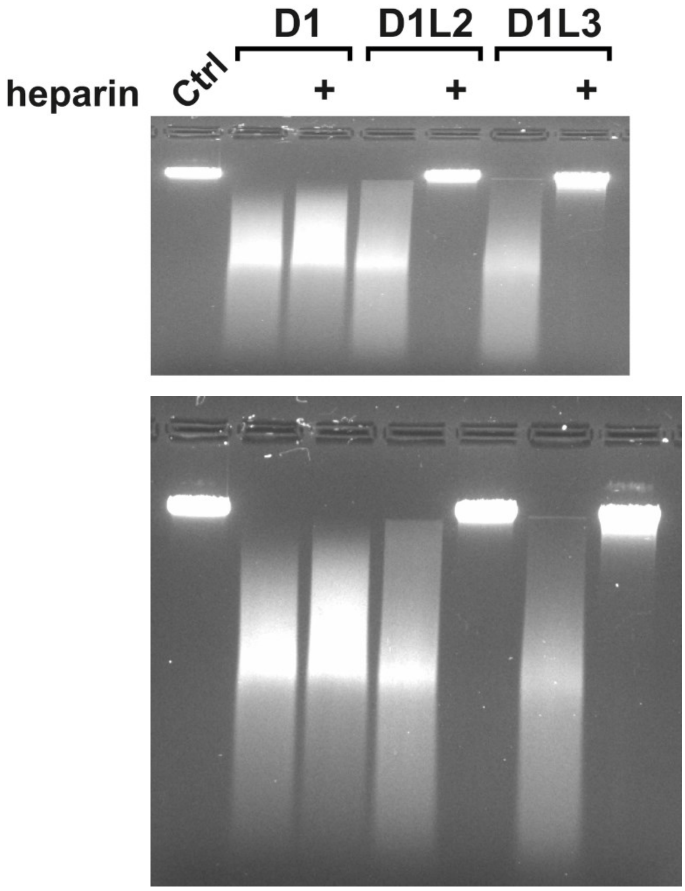
D1
D1L2
D1L3
heparin
Ctrl
+
+
+

C

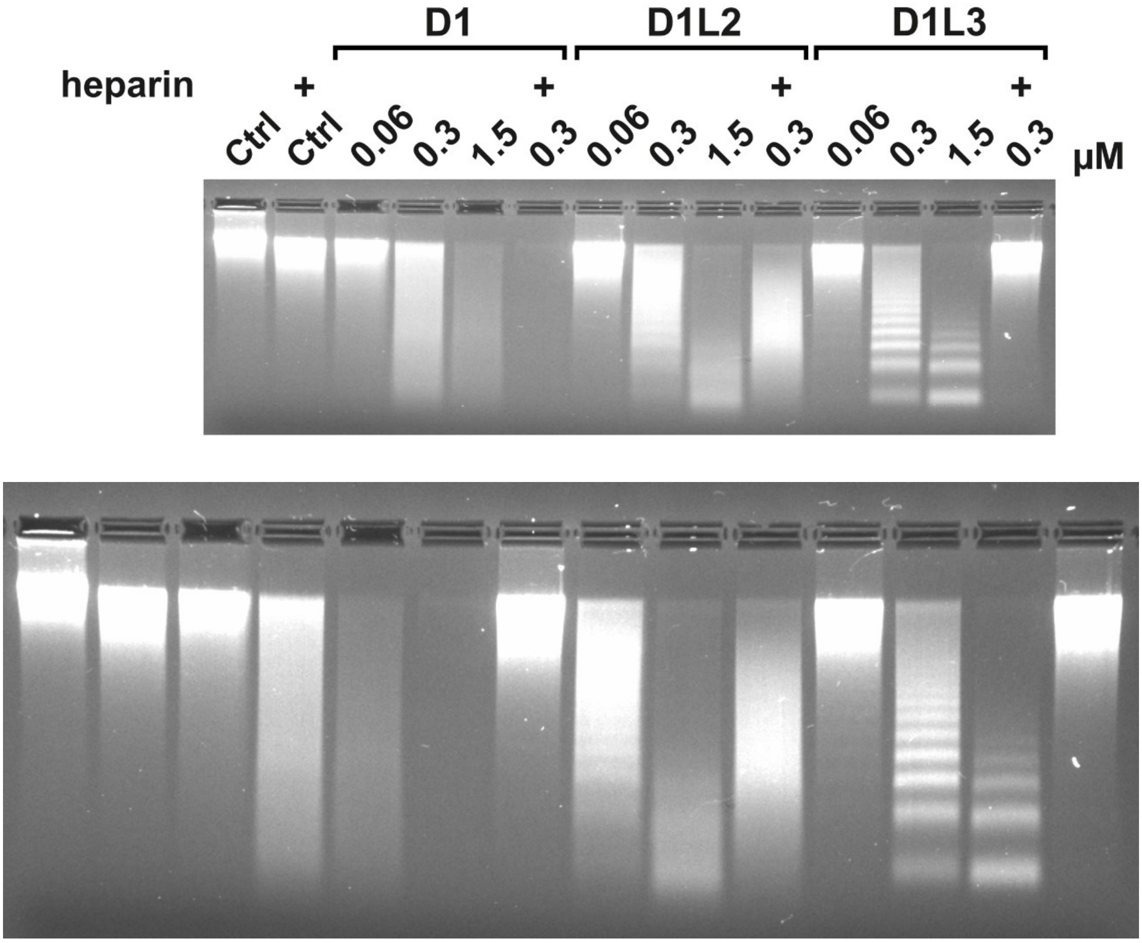
D1
D1L2
D1L3
heparin
+
+
+
+
Ctrl
Ctrl
0.06
0.3
1.5
0.3
0.06
0.3
1.5
0.3
0.06
0.3
1.5
0.3
μM

S1 Figure

All pictures were taken with the ChemiDoc™ XRS+ System with the ImageLab 5.0 software (Bio-Rad Laboratories).

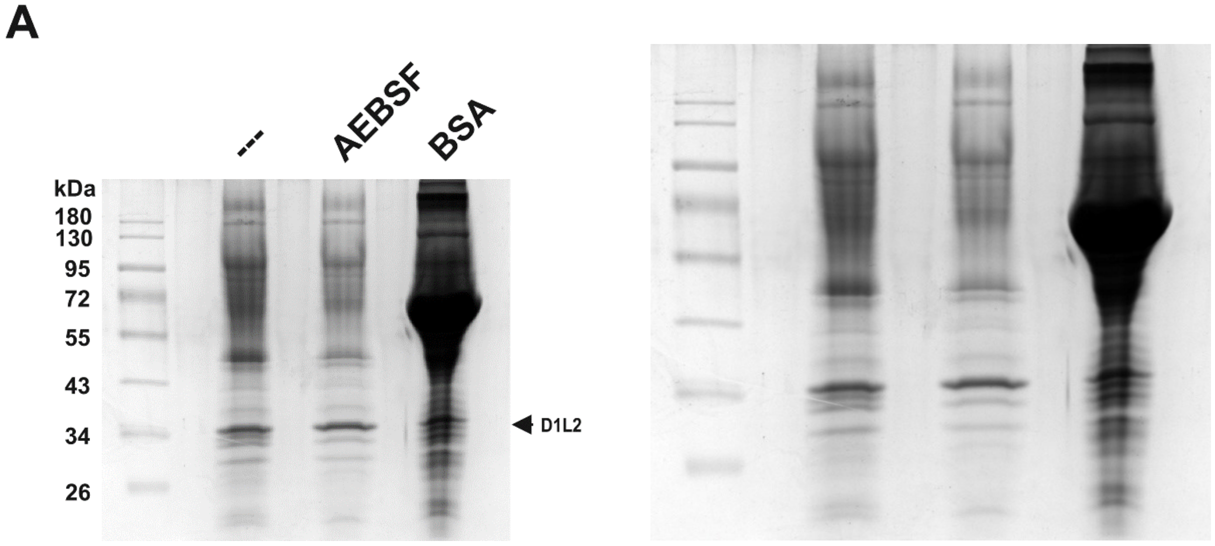

Marker: PageRuler Prestained Protein Ladder (ThermoFisher Scientific)

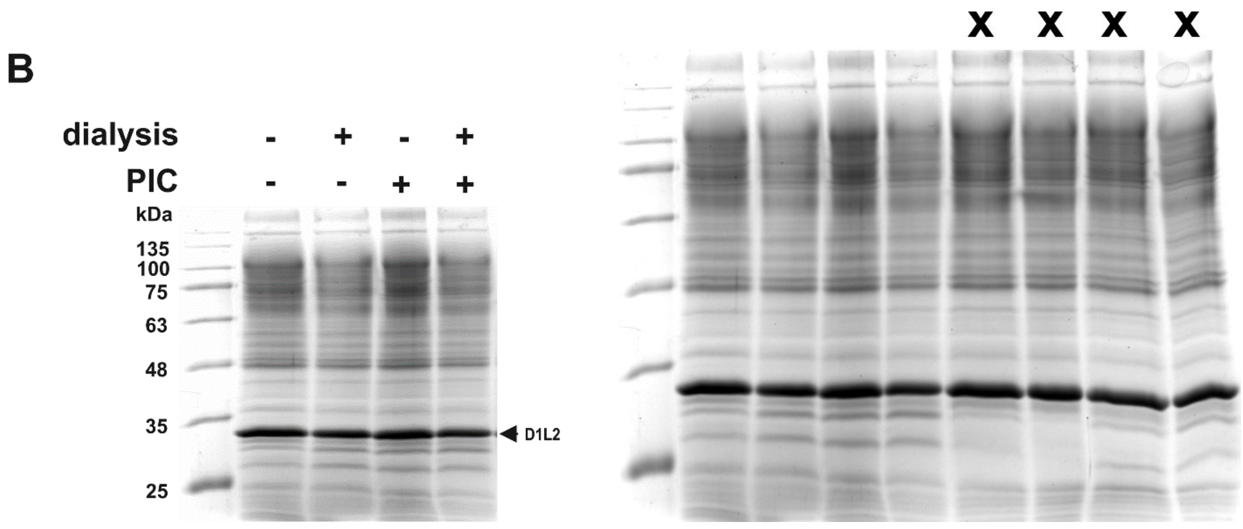

Marker: Cozy Prestained Protein Ladder (highQu)

S2 Figure

All pictures were taken with the ChemiDoc™ XRS+ System with the ImageLab 5.0 software (Bio-Rad Laboratories).

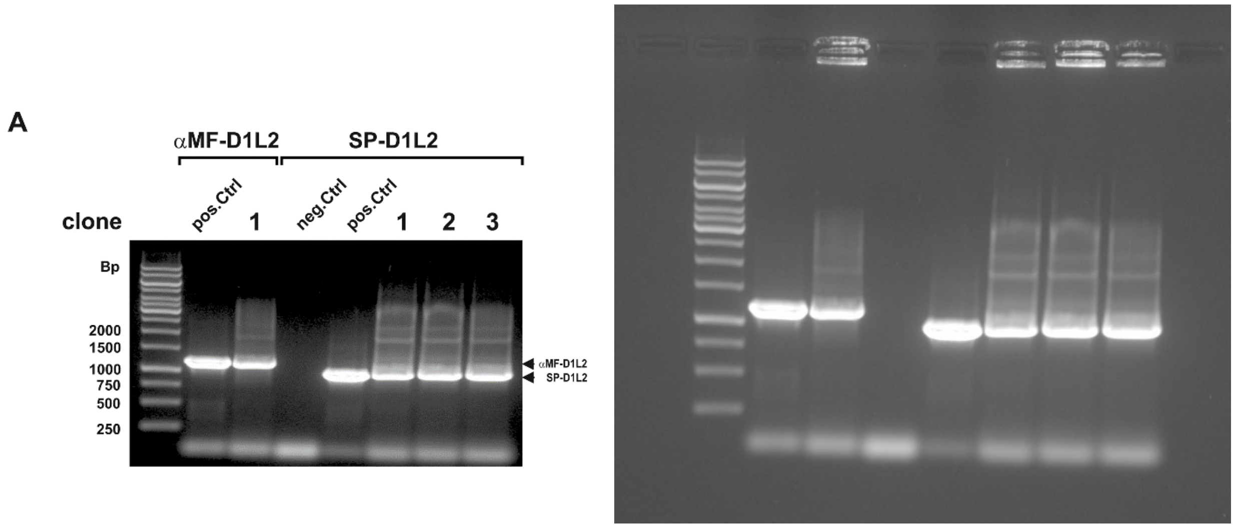

Marker: GeneRuler 1 kbp DNA Ladder (ThermoFisher Scientific)

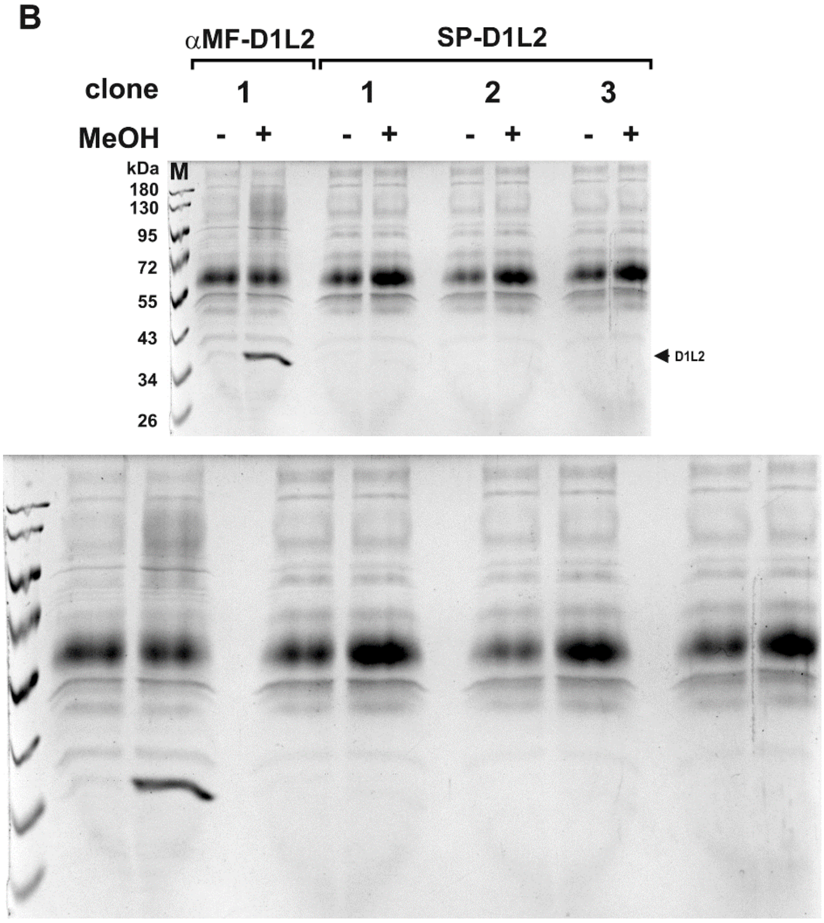

Marker: PageRuler Prestained Protein Ladder (ThermoFisher Scientific)

S3 Figure

All pictures were taken with the ChemiDoc™ XRS+ System with the ImageLab 5.0 software (Bio-Rad Laboratories).

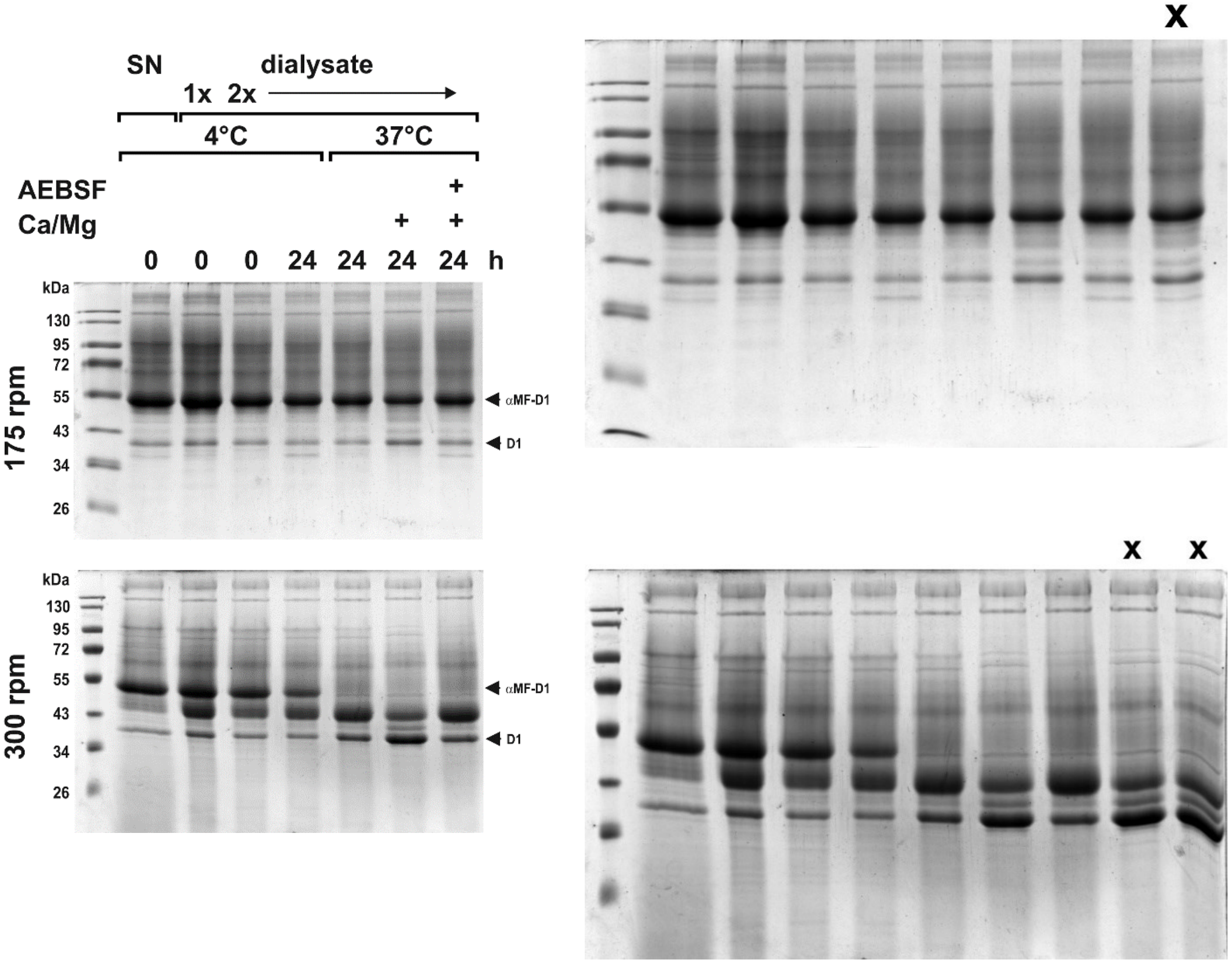

Marker: PageRuler Prestained Protein Ladder (ThermoFisher Scientific)
